# Supplementary material for: Clinical development of tacrolimus-resistant regulatory T cells to enable simultaneous immunosuppression and immune regulation
Source: Mol Ther Adv. 2026 Apr 9;34(2):201735. doi: 10.1016/j.omta.2026.201735 (PMC13175770; doi:10.1016/j.omta.2026.201735)
Supplement: Document S1. Figures S1–S6, Tables S1, S2, S3, and S6 [file mmc1.pdf]

OMTA, Volume *34*

Supplemental information

Clinical development of tacrolimus-resistant regulatory T cells to enable simultaneous immunosuppression and immune regulation

Ghazaleh Zarrinrad, Lisa-Marie Burkhardt, Claudia Beltran-Mestres, Silvina Romero-Suárez, Dimitrios Laurin Wagner, Stephan Schlickeiser, Maik Stein, Désirée Jacqueline Wendering, Iván Juky Carrera Diaz de la Cebosa, Lukas Ehlen, Frederik Hamm, Gavin L. Kurgan, Pawel Durek, Frederik Heinrich, Anamika Giri, Yaolin Pu, Kristy Ou, Henrike Hoffmann, Sandra Muench, Insa Stuewe, Sven Dolling, Oliver McCallion, Jaspal Kaeda, Jonas Kath, Sarah Schulenberg, Niklas Wiese, Abdolreza Nazari, Lena Peter, Samira Picht, Andrea Sánchez-Peña, Olalekan Usman, Christian Brommel, Rolf Turk, Garrett Rettig, Morgan Sturgeon, Thomas L. Osborne, Ashley Jacobi, Rebecca Friedrich, Masako Monika Kaufmann, Julia Klermund, Simon Fink, Markus F. Templin, Andy Roemhild, Daniel Kaiser, Oliver Klein, Toni Cathomen, Mir-Farzin Mashreghi, Fadi Issa, Julia K. Polánsky, Hans-Dieter Volk, Michael Schmueck-Henneresse, Petra Reinke, and Leila Amini

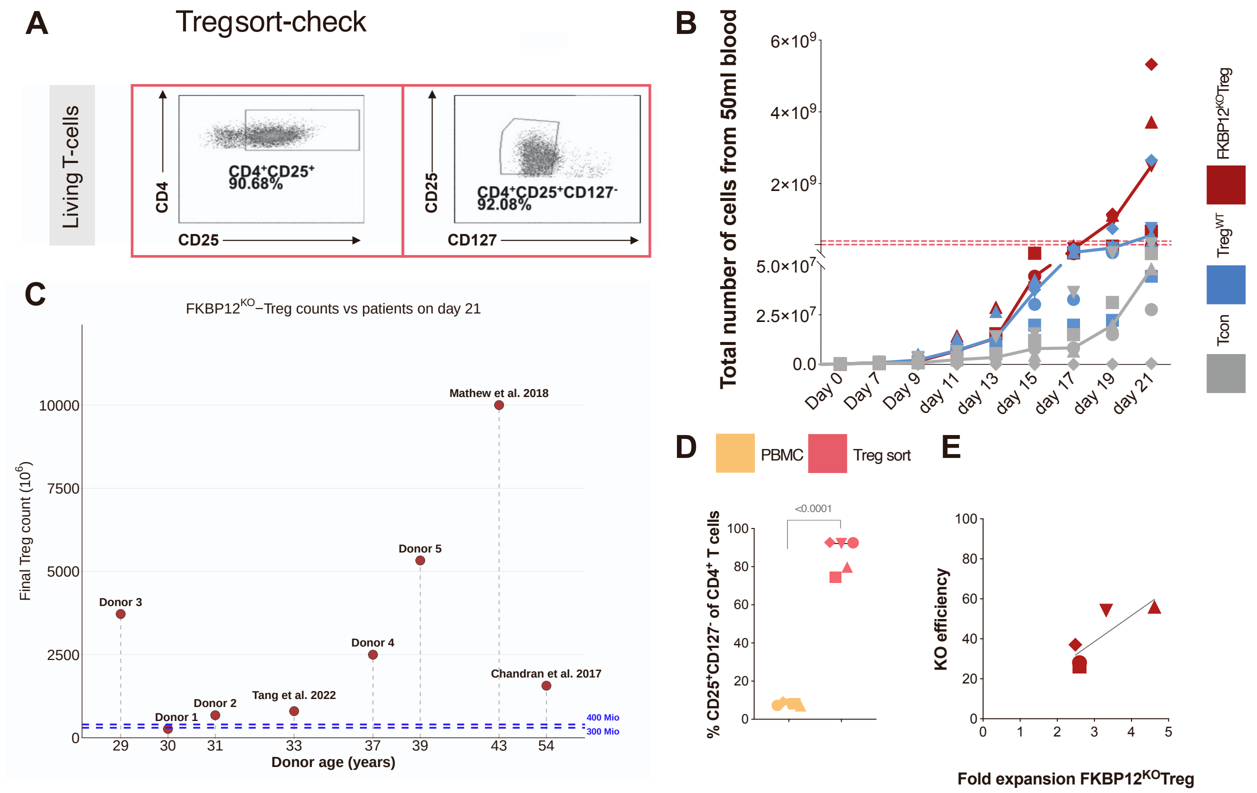

F

PMID	Cells	Disease	CD4+	CD4+CD25+	CD4+CD25+CD127-	Foxp3+	CD8+	Gene engineering rate
33666654	Treg	GVHD	-	>60%		60%	<10%	-
29743501	Treg	kidney transplantation	98-99%	>99%		>80%	<10%	-
28675676	Treg	kidney inflammation	>95%	-		>60%	<5%	-
32446407	Treg	kidney transplantation	-	-	92.4+/-3.2%	85.7+5.1%	-	-
33087345	Treg	kidney transplantation	96%	-	-	>70%	-	-
35240319	Treg	GVHD	-	-	-	90%	-	-
41083470	Treg	autoimmunity	-	-	-	-	-	67.4%
39905729	Treg	immunopathologies	-	-	90%	-	-	>80%
Zarrinrad et al.,	Treg	SOT	-	>85%	>85%	>85%	<0.002%	60-80%
TregTacRes	Treg	SOT, dialysis patients	x̄ 91.05%	-	-	x̄ 89.3%	x̄ 0.04%	x̄ 90.37%

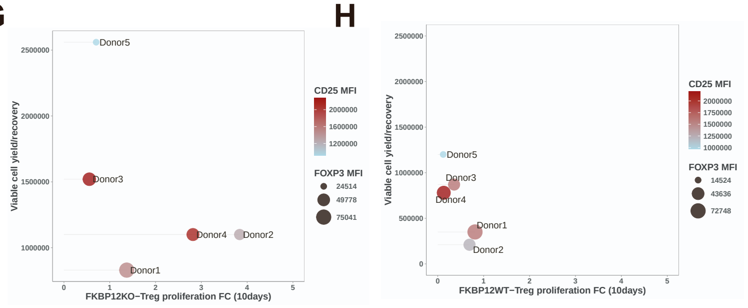

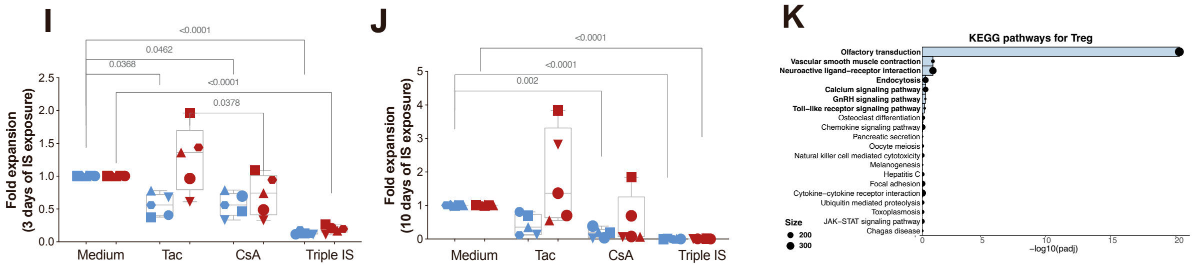

Figure S1: Tyto sort purity and Treg expansion

(A) Gating strategy for Tyto sort showing CD4, CD25 and CD127 gating. Remaining figures show data from individual donors by different symbols: (B) Visualization of the total number of expanding cells from day 0 to day 21. Tcon (grey), Treg^{WT} (blue), FKBP12^{KO}-Treg (teal), red line marks cut-off for Treg amount in clinical application. (C) Final number of expanded Treg on day 21 (10^6) per donor correlated to donor age (years) and plotted against the same parameters of 3 publications (Tang, Mathew and Chandran). (D) Sort purity shown as the percentage of $CD4^+CD25^+CD127^-$ T-cells pre- (yellow) and post sorting (light red). (E) The correlation between knock-out efficiency and fold expansion of FKBP12^{KO}-Treg (red). (F) Literature parameters on GMP compliant (top 6) and research grade Treg products (n=2) disease application, phenotype thresholds and gene-editing rates in comparison to Zarrinrad et al., and related TregTacRes trial (bottom 2). (G-H) Correlation of viable cell recovery and proliferation Fold Change (FC) with dot size representing FOXP3 MFI and color-code representing CD25 MFI. (I-J) Fold expansion of FKBP12KO-Treg and TregWT under 3 and 10 days of immunsuppressiva (IS) exposure respectively: data is shown for medium, tacrolimus (Tac), cyclosporine A (CsA) and triple immunosuppression (IS) combining tacrolimus, prednisolone and mycophenolic acid. Each donor is shown by a different symbol and paired t-tests and RM One-way Anova were used, n=5. (K) KEGG pathway analysis for differentially methylated positions between FKBP12^{KO}-Treg and Treg^{WT} and their biological context.

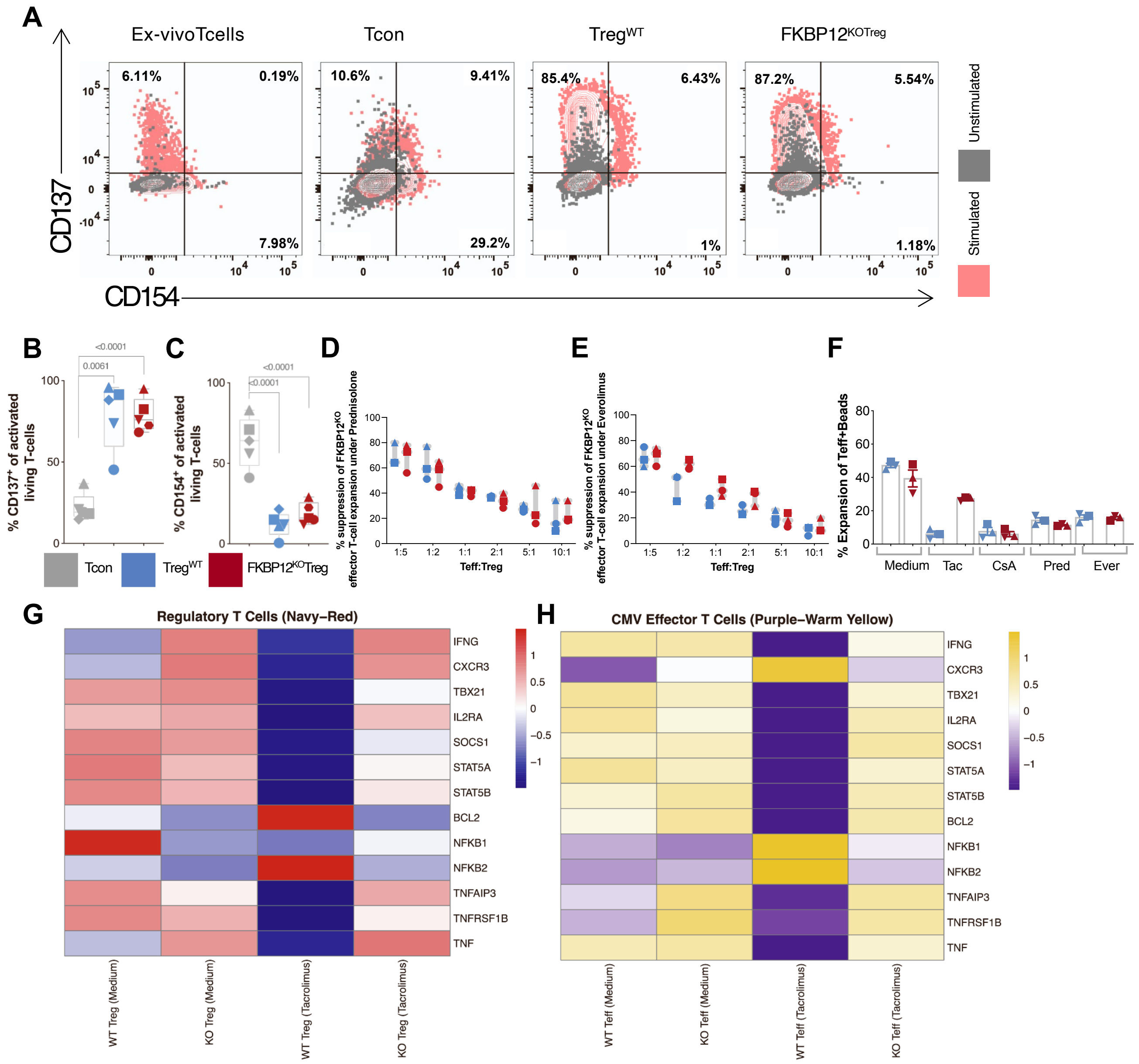

Figure S2: FKBP12^{KO}-Treg activation is comparable with Treg^{WT}

(A) Representative dot plots for CD137 and CD154 expression in ex-vivo T-cells, Tcon, Treg^{WT} and FKBP12^{KO}-Treg on day 21. Unstimulated controls (grey) and stimulated conditions (pink). Remaining figures show individual donors by different symbols. Tcon (grey), Treg^{WT} (blue), FKBP12^{KO}-Treg (red). (B) Percentage of CD137^{+} among the living CD4^{+} T-cells on day 21 in Tcon, Treg^{WT} and FKBP12^{KO}-Treg. (C) Percentage of CD154^{+} among the living T-cells on day 21 in Tcon, Treg^{WT} and FKBP12^{KO}-Treg. For (B) and (C) paired t-test and RM one-way Anova were used, n=5. (D-E) Frequency of suppression of FKBP12^{KO}-Teff in medium control, FKBP12^{KO}-Treg (red), Treg^{WT} (blue) is shown, with n=3 and for different Teff:Treg ratios under (D) prednisolone and (E) everolimus. (F) Proliferation potential of FKBP12^{KO}-Teff (red) and Teff^{WT} (blue) under different immunosuppressants without the influence of Treg. (G-H) Transcriptional profiling of key cytokine signaling cascades across engineered T cell populations. Teff are expanded, cryo-preserved CMV specific T-cells stimulated with CMV peptides IE1 and pp65 for 6h in comparison to Treg that were stimulated with PMA/Iono for 6h. Comparisons can only be made within one heat-map since scaling and experimental conditions differ. Combined heatmap depicting the averaged, scaled single-cell expression of highly curated gene panels associated with Interferon-gamma (IFN-γ), Interleukin-2 (IL-2), and Tumor Necrosis Factor-alpha (TNF-α) signaling pathways. Averaged expression is shown across Wild-Type (WT) and FKBP12-^{KO} Regulatory T Cells (Tregs) and corresponding CMV-Specific Effector T Cells (Teffs) in medium and tacrolimus conditions. Statistical & Visual Analysis: Raw single-cell RNA counts were aggregated and averaged per condition. To highlight relative shifts in pathway activation between treatments without signal washout between the distinct cell lineages, expression values were independently z-scored (scaled) across rows within each cell population prior to merging. The color gradient ranges from navy (downregulated/below mean) to white (mean expression) to firebrick red (upregulated/above mean). Genes exhibiting zero variance across conditions were algorithmically excluded to ensure matrix stability.

A

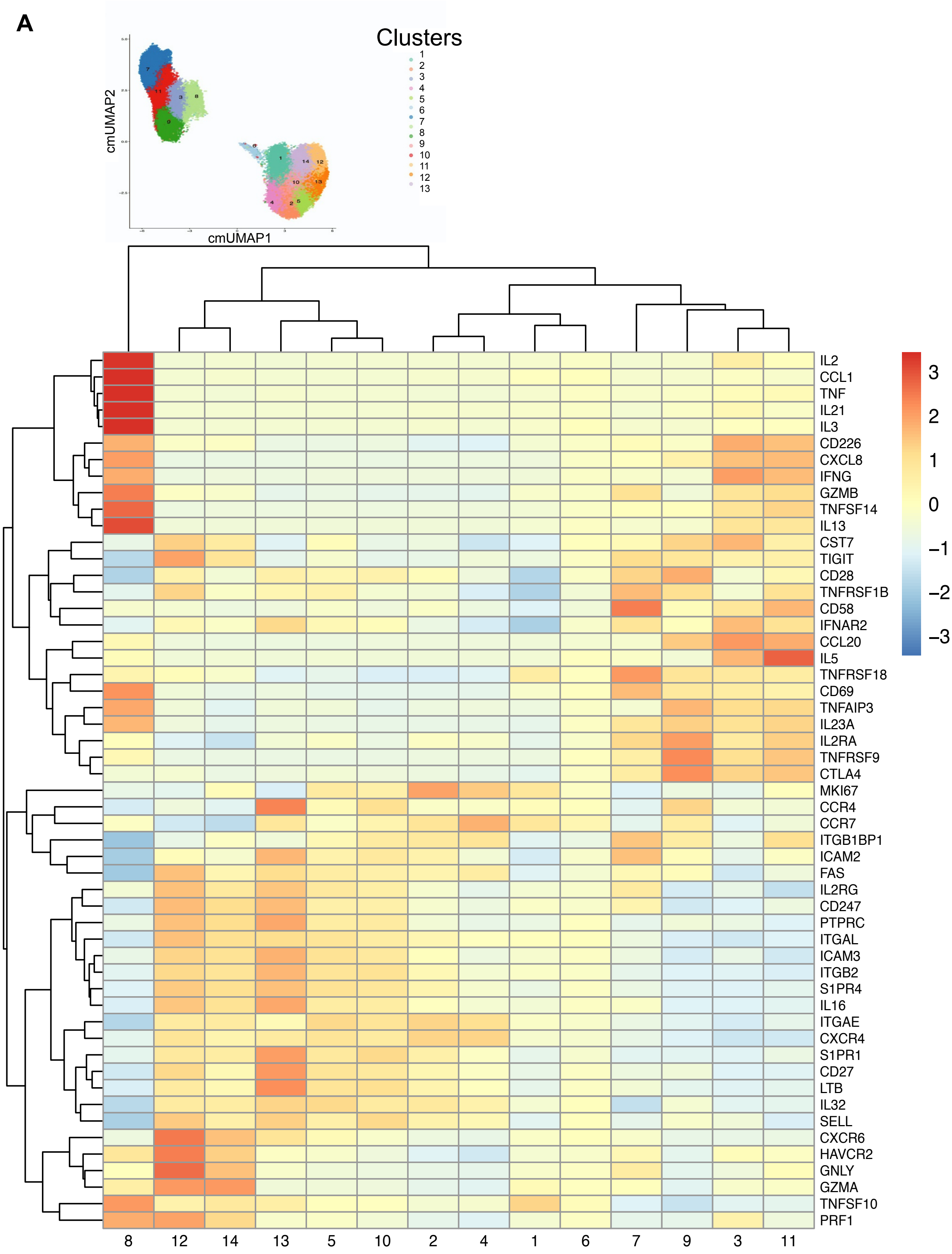

Figure S3: Cellular Indexing of Transcriptomes and Epitope sequencing

Detailed heatmap of RNA expression of T cell-associated genes within different clusters. Cluster distribution is shown for all 14 clusters and differential gene expression within each cluster (1-14) is highlighted within the heatmap.

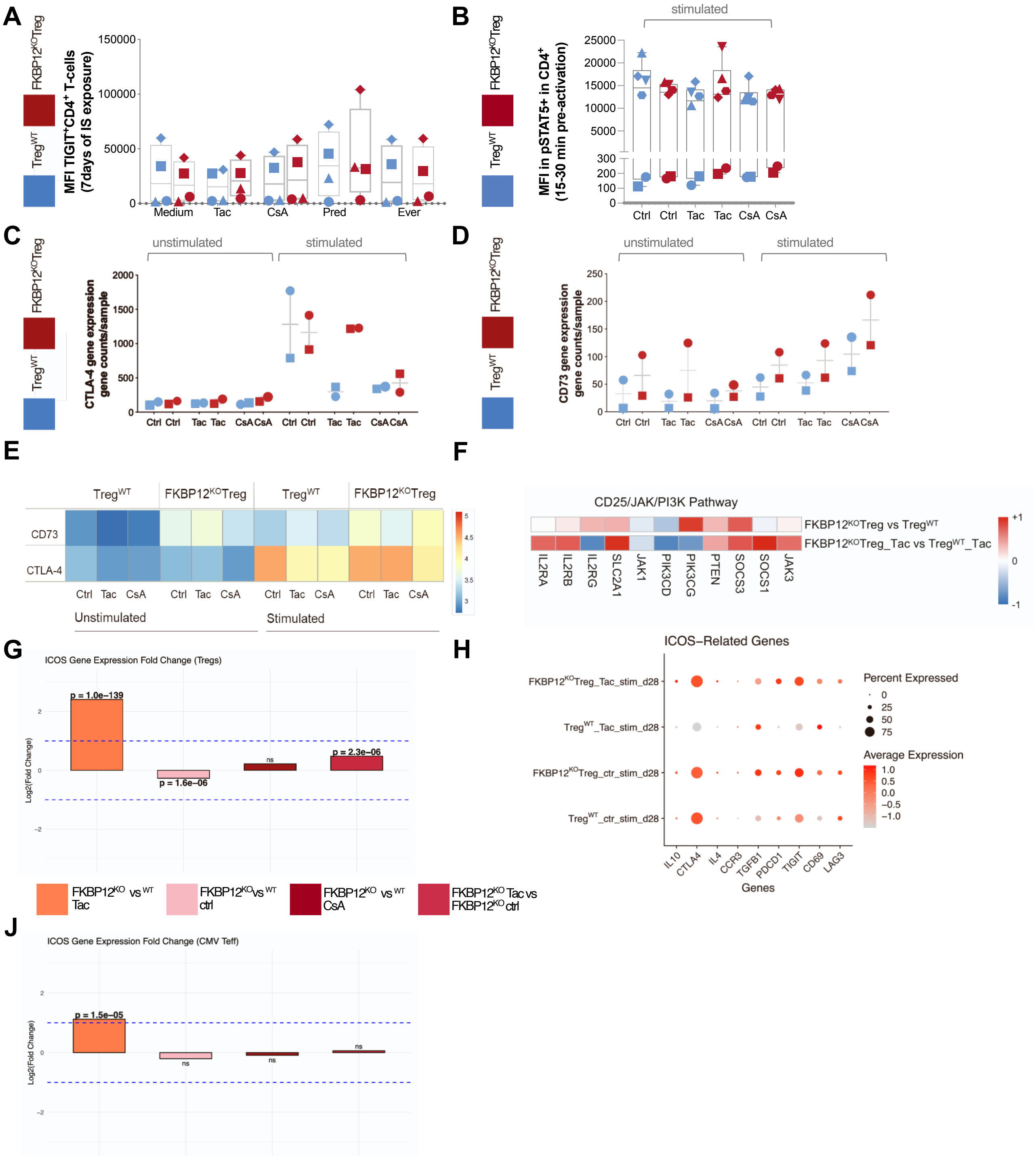

Figure S4: Beneficial functionality of FKBP12^{KO}-Treg in comparison to Treg^{WT}

Each symbol represents data from an individual donor. Tcon (grey), Treg^{WT} (blue), FKBP12^{KO}-Treg (red) n=2-6. For all figures conditions tacrolimus (Tac), cyclosporin A (CsA) and medium control (Ctrl) are shown. (A) Tigit expression in living CD4^{+} T-cells on day 21 in Treg^{WT} and FKBP12^{KO}-Treg under immunosuppressants including prednisolone (Pred) and everolimus (ever). (B) pSTAT5 MFI after 15-30 minutes stimulation by IL2 on day 21 in Treg^{WT} and FKBP12^{KO}-Treg substracted by unstimulated control. (C-D) CTLA-4 and CD73 gene expression levels are shown for Treg^{WT} and FKBP12^{KO}-Treg in unstimulated and stimulated states (6h PMA/Ionomycin activation) on day 28. Mean values of 2 technical replicates are displayed from 2 donors. (E) Heatmap of surface CD73 and CTLA-4 (CITE-seq antibody-derived tags) is shown for Treg^{WT} vs. FKBP12^{KO}-Treg in unstimulated and stimulated states (6h PMA/Ionomycin activation). (F) Heatmap visualizing differential expression for genes of JAK/STAT/PI3K pathways in FKBP12^{KO}-Tregs versus Treg^{WT} under tac and medium. Colour gradient = Log2 Fold Change (G) Bar graph shows Log2 Fold Change of ICOS expression in FKBP12^{KO} Tregs compared to Treg^{WT} under Tac, CsA and medium and a KO-only under Tac and medium. Statistics using non-parametric Wilcoxon rank-sum test. (H) Dot plot profiling an ICOS-related gene panel across FKBP12^{KO} and Treg^{WT} on day 28 for stimulated and unstimulated conditions. Size of dot = percentage of cells expressing target, colour intensity (light grey to red) = scaled average expression level. (I) Corresponding Log2 Fold Change of ICOS expression in FKBP12^{KO} CMV Teffs vs. WT across matching conditions. Counts aggregated into pseudo-bulk profiles and modelled using the edgeR quasi-likelihood (QL) framework with robust dispersion estimation. Horizontal lines indicate a 2-fold change (Log2FC = ±1). Exact adjusted p-values (ns = not significant; A = Bonferroni-adjusted; B = False Discovery Rate [FDR]).

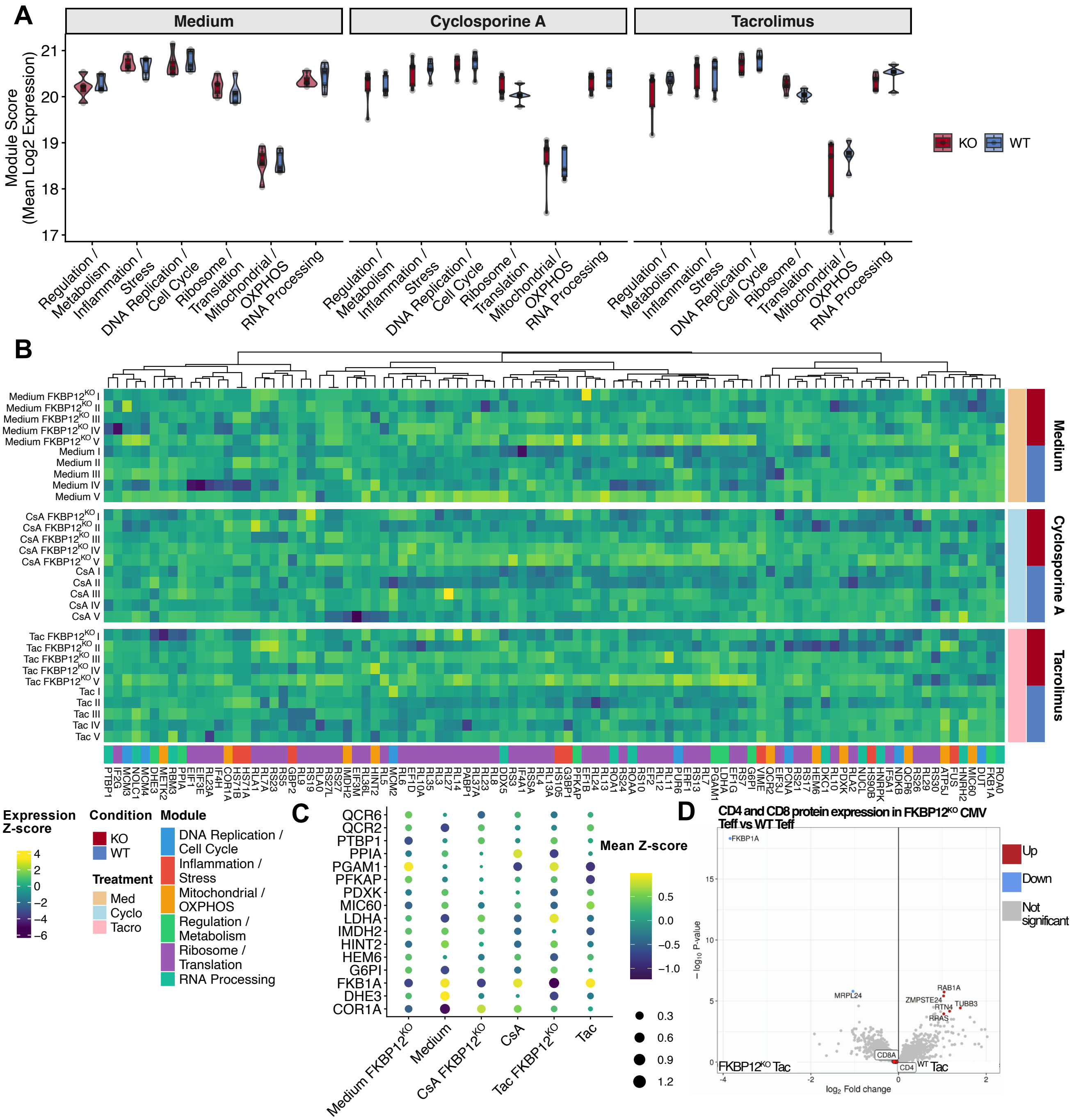

Figure S5: Comprehensive proteomic analysis reveals functional module differences between FKBP12^{KO}-Treg and Treg^{WT} under immunosuppressive treatment

(A) Violin plot showing comprehensive module analysis comparing FKBP12^{KO} (red) versus wild-type (blue) Tregs across three treatment conditions (Medium, cyclosporine A (CsA), and tacrolimus (Tac)). Six functional modules were analyzed: Regulation/Metabolism, Inflammation/Stress, DNA Replication/Cell Cycle, Ribosome/Translation, Mitochondrial/OXPHOS, and RNA Processing. Module scores represent the mean log2 expression values of constituent proteins. Each point = individual biological replicate (n=5 per group). Violin plots show the full distribution of data with overlaid boxplots and individual data points. (B) Heatmap displaying protein-level expression patterns across all experimental conditions. Rows represent individual proteins (n=98 unique proteins across all), coloured by functional module assignment (right annotation bar). Columns are organized by treatment condition Medium, CsA, Tac with wild-type (blue) and FKBP12^{KO} (red) samples grouped within each treatment (top annotation bars). Expression values are Z-score normalized across samples to highlight relative expression patterns. Proteins are hierarchically clustered using Euclidean distance and Ward's linkage method to group co-regulated proteins. The viridis color scale represents expression Z-scores from low (purple) to high (yellow). (C) Dotplot showing the mean expression Z-score of individual proteins within the Regulation/Metabolism module across all six experimental conditions. Each dot represents one protein averaged across n=5 biological replicates. Dot size encodes the absolute mean Z-score (|mean Z|), reflecting the magnitude of deviation from the protein's overall mean expression regardless of direction. Dot colour encodes the mean Z-score value, indicating both the direction and magnitude of expression change (viridis scale: purple = below average, yellow = above average). (D) volcano plot showing protein analysis of CD4 and CD8 protein in CMV Teffs is shown for FKBP12^{KO}-Teff (left) vs Teff^{WT} (right) with target genes labelled in light red, depicting Log2 Fold Change (FC) under tacrolimus treatment.

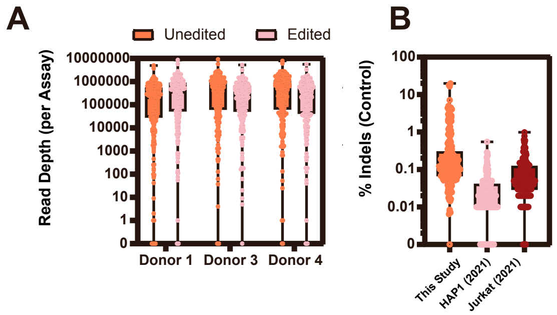

Figure S6: Off-target analysis of FKBP12^{KO}-Treg

Confirmatory sequencing information. (A) Following sequencing, the number of reads per off-target assay is shown for paired treatment and controls for each donor. (B) Indel frequencies of the unedited controls from all donors were quantified and compared to previously published frequencies in Kurgan et al. 2021 using the same bioinformatic analysis workflow.

Table S1: List of general antibodies
Summary of general Treg specific antibodies giving information on antibody, fluorescence label clone and concentration.

**Isolation and culture of Treg**	**Antibody**	**Fluorescence label**	**clone**	**concentration**
	anti-CD4	Vioblue	MT466	1 µg/mL
	anti-CD25	PE	3G10	1 µg/mL
	anti-CD127	APC	MB1518C9	1 µg/mL
**Phenotypic and functional assessment**	**Antibody**	**Fluorescence label**	**clone**	**concentration**
	anti-CD25	PE	MA251	1 µg/mL
	anti-CD127	APC	A019D5	2 µg/mL
	LIVE/DEAD™	UV	-	1:100 (manufacturer recommended)
	anti-CD3	BV650	OKT3	2 µg/mL
	anti-CD8	BV510	RPAT8	1 µg/mL
	anti-CD4	PerCP Cy5.5	SK3	1 µg/mL
	anti-FOXP3	AF488	259D	4 µg/mL
	anti-IL2	PE-Cy7	MQ117H12	4 µg/mL
	anti-TNFa	Alexa 700	MAb11	2 µg/mL
	anti-CD137	PE-Cy5	4B41	2 µg/mL
	anti-CD154	BV711	2431	2 µg/mL
	anti-IFNg	eFluor 450	4S.B3	2.5 µg/mL
	anti-CTLA4	PE-Cy5	BNI3	2 µg/mL
	anti-PD1	PE-Cy7	EH12.2H7	2 µg/mL

Table S2: List of materials and instruments
Summary of materials, supplements and instruments used giving information on material, concentration and company.

**Isolation and culture of Treg**	**Material/ Instrument**	**Company**	**Concentration**	**Additional information**
	EasySep™ Human CD4+ T-cell Enrichment Kit	Stemcell Technologies	-	
	CliniMACS Plus device	Miltenyi Biotec	-	
	anti-CD3/CD28-MACSiBeads	Miltenyi Biotec	-	
	X-Vivo-15	Lonza	-	
	fetal calf serum (FCS)	Biochrom	0,1ml/ml	
	penicillin/streptomycin	Biochrom	0,01ml/ml	
	rapamycin	Sigma Aldrich	1ul/ml	
	recombinant human IL-2	Miltenyi Biotec	1,250 IU/ml	
**FKBP12 knock-out in Treg**	**Material/ Instrument**	**Company**	**Concentration**	
	HiFi Cas9 Nuclease V3	IDT	10ug	
	2O'-methyl-3'phosphothioate-modified sgRNA	Synthego Corporation	15ug	Sequence: 5'-G*G*G*CGCACCUUCCCCAAGCG + mod-scaffold
	Amaxa-P3-primary-cell-4D-Nucleofector-X-Kit-L	ThermoFisher	-	
	Amaxa-Nucleofector-4D	Lonza	-	program CO-115
**Phenotypic and functional assessment**	**Material/ Instrument**	**Company**	**Concentration**	**Additional information**
	PMA		-	
	Ionomycin		-	
	FoxP3/Transcription Factor Staining Buffer kit	eBioscience	-	
	Fortessa	BD	-	
	Cytoflex LC	Beckman Coulter	-	
**Methylation profiling**	**Material/ Instrument**	**Company**	**Concentration**	

	Infinium-Methylation-EPIC-Kit	Illumina-EPIC-8-BeadChip	-	
	EZ-DNA-Methylation-Gold-Kit	Zymo Research	-	
	QIAquick-PCR-Purification-Kit	Qiagen	-	
**Cellular indexing of transcriptomes and epitopes sequencing (CITE-seq)**	**Material/ Instrument**	**Company**	**Concentration**	
	Single-Index-Kit-T-Set-A/Single-Index-Kit-N-Set A	10X Genomics	-	
	High-Sensitivity-DNA-Kit	Agilent	-	
	NextSeq500 device	Illumina	-	

Table S3: List of CITEseq antibodies
Summary of CITEseq antibodies giving information on reagent, clone, source and identifier.

REAGENT or RESOURCE	SOURCE	IDENTIFIER
Antibodies		
TotalSeq™-C0034 anti-human CD3 Antibody (UCHT1)	BioLegend	Cat# 300479
TotalSeq™-C0045 anti-human CD4 Antibody (SK3)	BioLegend	Cat# 344651
TotalSeq™-C0080 anti-human CD8a Antibody (RPA-T8)	BioLegend	Cat# 301071
TotalSeq™-C0185 anti-human CD11a (TS2/4)	BioLegend	Cat# 350617
TotalSeq™-C0085 anti-human CD25 (BC96)	BioLegend	Cat# 302649
TotalSeq™-C0386 anti-human CD28 (CD28.2)	BioLegend	Cat# 302963
TotalSeq™-C0176 anti-human CD39 (A1)	BioLegend	Cat# 328237
TotalSeq™-C0576 anti-human CD49d (9F10)	BioLegend	Cat# 304345
TotalSeq™-C0174 anti-human CD58 (TS2/9)	BioLegend	Cat# 330921
TotalSeq™-C0146 anti-human CD69 (FN50)	BioLegend	Cat# 310951
TotalSeq™-C0577 anti-human CD73 (AD2)	BioLegend	Cat# 344031
TotalSeq™-C0156 anti-human CD95 (DX2)	BioLegend	Cat# 305651
TotalSeq™-C0155 anti-human CD107a (H4A3)	BioLegend	Cat# 328649
TotalSeq™-C0246 anti-human CD122 (TU27)	BioLegend	Cat# 339021
TotalSeq™-C0390 anti-human CD127 (A019D5)	BioLegend	Cat# 351352
TotalSeq™-C0355 anti-human CD137 (4B4-1)	BioLegend	Cat# 309839
TotalSeq™-C0151 anti-human CD152 (BNI3)	BioLegend	Cat# 369621
TotalSeq™-C0032 anti-human CD154 (24-31)	BioLegend	Cat# 310849

TotalSeq™-C0140 anti-human CD183 (G025H7)	BioLegend	Cat# 353747
TotalSeq™-C0144 anti-human CD185 (J252D4)	BioLegend	Cat# 356939
TotalSeq™-C0071 anti-human CD194 (L291H4)	BioLegend	Cat# 359425
TotalSeq™-C0141 anti-human CD195 (J418F1)	BioLegend	Cat# 359137
TotalSeq™-C0143 anti-human CD196 (G034E3)	BioLegend	Cat# 353440
TotalSeq™-C0148 anti-human CD197 (G043H7)	BioLegend	Cat# 353251
TotalSeq™-C0152 anti-human CD223 (11C3C65)	BioLegend	Cat# 369335
TotalSeq™-C0171 anti-human CD278 (C398.4A)	BioLegend	Cat# 313553
TotalSeq™-C0088 anti-human CD279 (EH12.2H7)	BioLegend	Cat# 329963
TotalSeq™-C0169 anti-human CD366 (F38-2E2)	BioLegend	Cat# 345049
TotalSeq™-C0063 anti-human CD45RA (HI100)	BioLegend	Cat# 304163
TotalSeq™-C0087 anti-human CD45RO (UCHL1)	BioLegend	Cat# 304259
TotalSeq™-C0159 anti-human HLA-DR (L243)	BioLegend	Cat# 307663
TotalSeq™-C0089 anti-human TIGIT (A15153G)	BioLegend	Cat# 372729

Table S4: Database comparison of differentially expressed CITEseq genes in FKBP12^{KO}-Treg and Treg^{WT}
Summary of significantly regulated genes pre-selected from volcano plots comparing expression levels in FKBP12^{KO}-Treg and Treg^{WT}. Those genes further underwent DAVID database analysis and resulting gene dependent pathways are displayed.

Table S5: Off-target nomination sites
Summary of off-target nomination sites selected with the following criteria: 1) All sites that had overlap between one or more empirical method (abnoba-seq/guide-seq/UNCOVERseq); 2) All UNCOVERseq sites with Tiers 1 to 3 (our highest priorities); 3) All GUIDE-seq sites; 4) All Abnoba-seq sites; 5) All in silico sites with lev dist < 3 (reference or population >1%); 6) All in silico sites with lev dist <4 + an exonic region annotated (reference or population > 1%).

Table S6: Methods qualification of digital droplet PCR of FKBP12^{KO}-Treg

Qualification results for KO-efficacy read-outs for in total 4 assays indicating operator, date, assay, mean and standard deviation and reference Sanger-sequencing. Results qualification for KO-efficacy read-outs. Assays 1-4.

06.02. - 07.02.2025

Donor (D)																					
Operators	D1	D1	D2	D1																	
Date	07.02.25	07.02.25	06.02.25	06.02.25	A1	A1	A1	A2	A2	A2	A3	A3	A3	A4	A4	A4					
Assay	A1	A2	A3	A4	50	75	100	50	75	100	50	75	100	50	75	100					
1	90,45	90,34	91,14	90,55	47,66	73,99	99,03	49,58	72,43	97,67	50,99	75,37	100,00	49,05	74,09	99,88					
2	90,37	90,32	91,54	90,10	47,93	73,09	98,55	47,60	72,34	97,25	50,04	75,16	100,00	48,90	74,22	99,80					
3	90,27	89,64	91,61	90,03																	
4	90,02	89,80	90,99	89,77																	
5	90,27	90,42	91,53	89,79																	
6	90,57	90,05	90,89	90,28																	
7	90,41	89,78	91,05	90,14																	
8	89,60	90,08	91,34	89,06																	
Mean	90,24	90,05	91,26	89,96		73,54			72,39			75,27			74,15						

standard deviation	0,31	0,29	0,28	0,44																
coefficient of variation	0,34	0,33	0,30	0,49																
Reference Sanger-seq.	93,00	91,00	99,00	92,00																
	Intra-A	Inter-A I	Inter-O I	Ges.	Inter-O II	Inter-A II	A1	A1	A1	A2	A2	A2	A3	A3	A3	A4	A4	A4	A1-A4	Sanger Seq.
	A1, A2	A1, A4	A3, A4	A1, A2, A3, A4	A1, A3	A2, A3	50	75	100	50	75	100	50	75	100	50	75	100	75	
Mean	90,15	90,10	90,61	90,38	90,75	90,66	47,80	73,54	98,79	48,59	72,39	97,46	50,52	75,27	100,00	48,98	74,15	99,84	73,84	93,75
standard deviation	0,31	0,40	0,76	0,62	0,60	0,68														
coefficient of variation	0,34	0,44	0,84	0,68	0,66	0,75														
% of deviation Assay vs. Reference																				-3,59

Supplemental methods

Computational Analysis – CITE seq

Differential Expression and Variance Modeling

To ensure maximum statistical rigor, the differential expression models in this study were dynamically adapted to the biological structure of the data and the sample sizes of the specific comparisons being queried. All frameworks strictly corrected for the multiple testing problem, completely avoiding the use of raw, unadjusted p-values.

1. Global Population Dynamics (edgeR & FDR)

For analyses evaluating broad transcriptional shifts across entire experimental conditions—including the Cytomegalovirus-specific effector T cell (CMV Teff) populations ($N=4$ donors), JAK/STAT pathway mapping, global cytokine downregulation, and overall Treg phenotypic profiling—we utilized a pseudo-bulk edgeR framework. By aggregating counts per biological replicate, this gold-standard approach accurately models true donor-to-donor variance and eliminates single-cell pseudoreplication. For CMV Teffs, counts were aggregated into pseudo-bulk profiles and differential expression was modeled using the edgeR quasi-likelihood (QL) framework with robust dispersion estimation. Significance for these comparisons was defined using the False Discovery Rate (FDR, Benjamini-Hochberg procedure) to robustly identify true biological shifts across the population.

2. Intra-Population Slicing & Subsetting (Wilcoxon & Bonferroni)

For highly granular queries requiring threshold-gated subsetting within a condition (e.g., isolating 'ICOS-High' versus 'ICOS-Negative' cells), pseudo-bulk aggregation carries a

severe risk of mathematical instability due to ‘cell starvation’ (i.e., generating pseudo-bulk profiles from statistically insufficient cell numbers per donor). Similarly, for the Treg compartment, the limited availability of biological replicates ($N=2$ donors) precluded reliable pseudo-bulk dispersion estimation. To bypass these sparsity limitations and maintain statistical power, we leveraged single-cell resolution using the non-parametric Wilcoxon rank-sum test. To aggressively guard against the inherent pseudoreplication of single-cell tests, we applied the highly conservative Bonferroni correction.

Transcriptional Profiling and Visualization

- ICOS Modulation Analysis: ICOS expression was evaluated as a Log2 Fold Change in FKBP12-KO cells compared to Wild-Type (WT) across distinct culture contexts (Tacrolimus, Cyclosporine A [CsA], and medium).
- Dot Plots: The size of each dot represents the percentage of cells within the condition expressing the target gene, while the color intensity (ranging from light grey to red or deep blue) indicates the scaled average expression level.
- Volcano Plots: Differential expression was depicted with target genes highlighted against a background of all detected transcripts (grey). The y-axis represents the $-Log_{10}$ adjusted p-value. Vertical dashed blue lines denote a 2-fold expression shift ($Log_{2}FC = \pm1$), and the horizontal dashed blue line signifies statistical significance (Adj. $p < 0.05$). Label overlap was algorithmically disabled to ensure comprehensive identification of all target markers.
- Pathway Heatmaps: Raw single-cell RNA counts were aggregated and averaged per condition. To highlight relative shifts in pathway activation (IFN-γ, IL-2, and TNF-α) without signal washout between the distinct cell lineages, expression

values were independently z-scored (scaled) across rows within each cell population prior to merging. Columns were explicitly ordered to visualize the biological progression from untouched controls to KO/Tacrolimus contexts, and genes exhibiting zero variance were algorithmically excluded to ensure matrix stability.

- Bubble Plots: Visualized differential expression for key members of the JAK/STAT and PI3K pathways. The color gradient indicates Log_{2} Fold Change, while bubble size is proportional to statistical certainty ($-Log_{10}$ FDR or Bonferroni, depending on the model).

Defined Gene Panels and Functional Subsetting

ICOS-High Subset: ICOS-positivity was defined mathematically by calculating the median expression threshold of all baseline non-zero expressing cells. This pre-defined ICOS-related gene panel was then profiled across conditions.

Phenotypic Markers: Tregs were profiled using established "Good" markers (e.g., FOXP3, MKI67, CTLA4) and "Bad" markers (e.g., IL7R/CD127, KLRG1) to assess stability. Additionally, cell-cycle and proliferation genes (CCND1, PCNA, MKI67) and terminal exhaustion receptors (TOX, LAG3, PDCD1) were tracked to confirm robust proliferation without inducing terminal exhaustion.

Cytokine Filtering: Cytokine and chemokine transcripts (IL, IFN, TNF, CXCL, CCL families) were strictly pre-filtered for significance and negative fold change ($LogFC < 0$).

Computational Analysis – Confirmation

Analysis of the sequencing data to identify confirmed off-target editing at the nominated sites was performed using CRISPAltRations v1.2.1. For identifying indels, the window for

event quantification was centered on the canonical cut site and events quantified utilizing the default window size for Cas9 (8 bp). To determine whether indels found in the sequencing data could result from bona fide off-target cleavage, indels were grouped by location relative to the cut site (prioritizing minimum distance to cut site) followed by fitting counts of events to a negative binominal model with a Wald test for significance in each location bin per off-target using the DESeq2 package within IDT's OTEasy tool (Schmaljohn et al., Manuscript in Preparation). For classification of indel off-target editing, the tool requires: 1) sufficient read coverage for the site (>1,000x) in all replicates, 2) significant edits to occur at or adjacent to the cut site after optimal alignment, 3) the classified cumulative significant edits to exceed 0.01%, 4) the comparison of treatment/control samples at the site to have a significant adjusted p-value ($p < 0.05$), and 5) an average coverage frequency of at least 5x the ascribed cumulative frequency observed (e.g., for 0.1% editing, at least 5,000x coverage).

Computational Analysis – Translocations

To quantify translocations from editing, Primer Anchored Statistical Translocation Analysis (PASTA) was used (Kurgan et al., Manuscript in Preparation). This analysis is only performed on the amplicon sequencing pools containing the on-target edit (because multiplexed amplification is a requirement for event detection using the method), and reactions not containing the on-target edit are unlikely to have any significant translocation events. To quantify translocations, expected primers were identified in reads using fg-idprimer (https://github.com/fulcrumgenomics/fg-idprimer; -k=6, -K=8, -S=5, --max-mismatch-rate=0.07). Following this, treatment/control pairs had their counts paired and primer count frequencies subjected to a one-tailed hypergeometric test with Benjamini-Hochberg correction (statsmodel v0.15.0; default settings) to calculate an adjusted p-

value (p-adj). Unexpected primer pairs with padj < 0.01 with no flags were classified as a translocation and had the translocation frequency (P) calculated using the following equation S1:

$$P_t = \frac{n_t}{f_{total}} + \frac{n_t}{r_{total}}$$

Where 'n' is equal to the count of the unexpected primer pair of interest, 't' is the significant translocation being interpreted, 'f' is the total count of the shared forward primer events excluding the count participating in the 'n' translocation event, and 'r' is the total count of shared reverse primer events excluding the count participating in the 'n' translocation event. The translocation frequency is then adjusted by the background level frequency in the control by subtracting any translocation frequency observed in the control sample from the treatment frequency. Total translocation burden (B) was calculated using the following equation S2:

$$B = 1 - \prod_{t}^{t_n} (1 - P_t)$$

Where 't' is equal to a significant translocation, and 'tn' is equal to the last significant translocation of all translocations. All translocations for the purposes of this equation are assumed to be occurring independently. Using the method, translocations are quantified if: 1) the estimated frequency exceeds 0.1% of editing, 2) the translocation has a significant p-value ($p < 0.01$), and 3) the translocation is found to meet these criteria in all replicates.
