# Supplementary material for: Clinical development of tacrolimus-resistant regulatory T cells to enable simultaneous immunosuppression and immune regulation
Source: Mol Ther Adv. 2026 Apr 9;34(2):201735. doi: 10.1016/j.omta.2026.201735 (PMC13175770; doi:10.1016/j.omta.2026.201735)
Supplement: Document S2. Article plus supplemental information [file mmc4.pdf]

Molecular Therapy
Advances
Original Article

Clinical development of tacrolimus-resistant regulatory T cells to enable simultaneous immunosuppression and immune regulation

Ghazaleh Zarrinrad,[1,2,3,15] Lisa-Marie Burkhardt,[1,4,15] Claudia Beltran-Mestres,[1,4] Silvina Romero-Suárez,[1,3] Dimitrios Laurin Wagner,[3,4,6] Stephan Schlickeiser,[3] Maik Stein,[3] Désirée Jacqueline Wendering,[3] Iván Juky Carrera Diaz de la Cebosa,[4] Lukas Ehlen,[3,13] Frederik Hamm,[3] Gavin L. Kurgan,[5] Pawel Durek,[7] Frederik Heinrich,[7] Anamika Giri,[3] Yaolin Pu,[1,4] Kristy Ou,[3] Henrike Hoffmann,[4] Sandra Muench,[4] Insa Stuewe,[4] Sven Dolling,[4] Oliver McCallion,[8] Jaspal Kaeda,[1] Jonas Kath,[3,4] Sarah Schulenberg,[2,3] Niklas Wiese,[2,3] Abdolreza Nazari,[1,2] Lena Peter,[3] Samira Picht,[2,3] Andrea Sánchez-Peña,[5] Olalekan Usman,[5] Christian Brommel,[5] Rolf Turk,[5] Garrett Rettig,[5] Morgan Sturgeon,[5] Thomas L. Osborne,[5] Ashley Jacobi,[5] Rebecca Friedrich,[3] Masako Monika Kaufmann,[9,14] Julia Klermund,[9] Simon Fink,[11] Markus F. Templin,[11] Andy Roemhild,[4] Daniel Kaiser,[4] Oliver Klein,[12] Toni Cathomen,[9,10] Mir-Farzin Mashreghi,[7] Fadi Issa,[8] Julia K. Polánsky,[3,7] Hans-Dieter Volk,[1,6] Michael Schmueck-Henneresse,[3] Petra Reinke, MD,[1,6] and Leila Amini[3,4]

[1]Charité – Universitaetsmedizin Berlin, Corporate Member of Freie Universitaet Berlin and Humboldt-Universitaet zu Berlin, Institute of Medical Immunology, Augustenburger Platz 1, 13353 Berlin, Germany; [2]Charité – Universitaetsmedizin Berlin, Corporate Member of Freie Universitaet Berlin and Humboldt-Universitaet zu Berlin, Berlin School for Regenerative Therapies, Augustenburger Platz 1, 13353 Berlin, Germany; [3]Berlin Institute of Health (BIH) at Charité – Universitaetsmedizin Berlin, BIH-Center for Regenerative Therapies (BCRT), Charitéplatz 1, 13353 Berlin, Germany; [4]Charité – Universitaetsmedizin Berlin, Corporate Member of Freie Universitaet Berlin and Humboldt-Universitaet zu Berlin, Berlin Center for Advanced Therapies, Augustenburger Platz 1, 13353 Berlin, Germany; [5]Integrated DNA Technologies (IDT) Inc, Coralville, IA 52241, USA; [6]TCbalance Biopharmaceutics GmbH, 10117 Berlin, Germany; [7]German Rheumatism Research Center, Institute of Leibniz-Gemeinschaft, Charitéplatz 1, 10117 Berlin, Germany; [8]Transplantation Research Immunology Group, Nuffield Department of Surgical Sciences, University of Oxford, John Radcliffe Hospital, Oxford OX3 9DU, UK; [9]Institute for Transfusion Medicine and Gene Therapy, Medical Center – University of Freiburg, 79106 Freiburg, Germany; [10]Center for Chronic Immunodeficiency, Faculty of Medicine, University of Freiburg, 79106 Freiburg, Germany; [11]NMI Natural and Medical Sciences Institute at the University of Tübingen, 72770 Reutlingen, Germany; [12]Berlin Institute of Health (BIH) at Charité – Universitaetsmedizin Berlin, 13353 Berlin, Germany; [13]Charité - Universitaetsmedizin Berlin, Corporate Member of Freie Universitaet Berlin, Humboldt-Universitaet zu Berlin, Department of Anesthesiology and Intensive Care Medicine (CCM/CVK), 13353 Berlin, Germany; [14]Spemann Graduate School of Biology and Medicine (SGBM), University of Freiburg, 79104 Freiburg, Germany

Unwanted alloimmune responses are a central driver of solid organ transplant rejection and currently managed with life-long immunosuppression, which imposes substantial risks and burdens on patients. Adoptive transfer of regulatory T cells (Tregs) offers a strategy to restore immunological balance and reduce long-term adverse effects of generalized immunosuppression. However, although Tregs can potently inhibit incipient immune activation, they struggle to suppress established memory effector T cells, necessitating the continued use of immunosuppression. Calcineurin inhibitors such as Tacrolimus effectively control both, newly activated and pre-existing effector T cells, but unfortunately, they also impair Treg function. Therefore, we hypothesized that gene-editing of Tregs inducing tacrolimus resistance (FKBP12^{KO}) would enable combined therapy that curbs effector T cell responses without compromising Treg efficacy. Here, we developed FKBP12^{KO}-Tregs using a ribonucleoprotein-based CRISPR-Cas9 approach and characterized them extensively *in vitro*. FKBP12^{KO}-Tregs retained phenotype, high viability, and suppressive function comparable to unedited Treg^{WT}, and they remained functionally impervious to Tacrolimus, while preserving sensitivity to alternative CNIs. We additionally established a good-manufacturing practice process for FKBP12^{KO}-Tregs. Comprehensive *in vitro* phenotypic, functional, and molecular characterization, together with the established manufacturing, provide the rationale for a proof-of-concept clinical trial assessing the feasibility and safety

Received 12 December 2025; accepted 7 April 2026;
https://doi.org/10.1016/j.omta.2026.201735.

[15]These authors contributed equally

Correspondence: Leila Amini, Berlin Institute of Health BIH at Charité – Universitaetsmedizin Berlin, BIH Center for Regenerative Therapies BCRT and Berlin Center for Advanced Therapies BeCAT at Charité – Universitaetsmedizin Berlin, Augustenburger Platz 1, D-13353 Berlin, Germany.
E-mail: leila.amini@charite.de

© 2026 The Author(s).
Published by Elsevier Inc. on behalf of The American Society of Gene and Cell Therapy.
This is an open access article under the CC BY license (http://creativecommons.org/licenses/by/4.0/).

of co-administration of FKBP12^{KO}-Tregs with Tacrolimus in living-donor kidney transplant recipients.

INTRODUCTION

Allo-transplantation provides a clear starting point for undesired immune reactivity, leading to anti-allograft immune responses in extreme cases causing graft rejection. Currently, transplant patients are managed with a cocktail of immunosuppressants, including calcineurin inhibitors (CNIs), such as tacrolimus (FK506) or cyclosporine A (CsA), which are associated with significant adverse effects for both patients and society at large.[1–5] To overcome the challenges of chronic immunosuppression, it is envisioned that standard drug therapy could be supplanted or tapered with the help of adoptive regulatory T cell (Treg) therapy. Stable Tregs are characterized by constitutively high expression of the interleukin-2 (IL-2) receptor α-chain cluster of differentiation (CD)25, driven by the lineage-defining transcription factor Forkhead-Box-Protein P3,[6] along with low expression of the IL-7 receptor α-chain (CD127).[7] Stable immunomodulatory Tregs are also distinguished from activated conventional T cells (Tcon) and unstable induced Treg by a demethylated FOXP3 gene enhancer region known as the Treg-specific demethylation region (TSDR).[8–12]

Clinical proof of concept for autologous Treg therapy in transplantation has already been demonstrated. In a phase 1/2a study at our institution, adoptive transfer of autologous Tregs enabled reduction of triple-drug immunosuppression to low-dose tacrolimus monotherapy in the majority of living-donor kidney transplant recipients.[13,14] While this finding demonstrates Tregs' therapeutic potential, it also highlights a key limitation. CNIs not only affect preformed alloreactive effector T cells,[15] but also impair the function and proliferation of Tregs.[16] Therefore, a central question is how to integrate Treg therapy with necessary CNI treatment. We addressed this by developing a tacrolimus-resistant Treg product (FKBP12^{KO}-Treg)[17,18] through clustered regularly interspaced short palindromic repeats (CRISPR)-Cas9-mediated knockout of FKBP1A (which encodes FK506-binding protein 12 [FKBP12], the intracellular adaptor protein for tacrolimus). This next-generation cell therapy is designed to maintain Treg activity even in the presence of tacrolimus, thereby potentially allowing simultaneous pharmacologic immunosuppression and Treg-mediated regulation.

In this study, we characterized FKBP12^{KO}-Tregs in comparison to conventional unedited Tregs (hereafter Treg^{WT}) produced under identical conditions. Our assays demonstrated that FKBP12^{KO}-Tregs preserve a stable Treg phenotype and epigenetic identity, with functional properties closely matching those of Treg^{WT}. In-depth transcriptomic and proteomic profiling further revealed that FKBP12^{KO}-Tregs possess a favorable immunoregulatory profile, especially under tacrolimus exposure, compared to Treg^{WT}. Additional safety evaluations, including clinical trial application compatible off-target nomination and validation show no reproducible edits or aberrant features in the FKBP12^{KO}-Tregs. Moreover, we were granted manufacturing authorization for a good manufacturing practice (GMP) process to generate the FKBP12^{KO}-Treg product (termed "TregTacRes") from the Landesamt für Gesundheit und Soziales Berlin (State Office for Health and Social Affairs Berlin, LAGeSo) with a process achieving high cell viability and yield. Taken together, these results support the translational advancement of FKBP12^{KO}-Tregs. Based on the findings presented here, a first-in-human clinical trial is planned to evaluate the feasibility of administering FKBP12^{KO}-Tregs in combination with low-dose tacrolimus in transplant recipients.

RESULTS

FKBP12^{KO} is feasible in Treg products

To avoid gene editing of contaminating, potentially allo-reactive effector T cells, Treg isolation was optimized for high purity prior to genetic modification by a combination of CD4 enrichment during density gradient centrifugation succeeded by flow cytometry sorting (Figures 1A, 1B, and S1A).[14] Sorted Treg were subsequently stained for FOXP3, the master transcription factor of Treg (Figure 1B). Sorting reliably yielded cell fractions with a minimized contamination of <2% of CD3$^-$ (Figure 1C), <0.05% CD3$^+$CD8$^+$ cells (Figure 1D) and achieved >90% purity of the CD25$^+$FOXP3$^+$/CD127$^-$CD25$^+$ Tregs within CD4$^+$ cells (Figures 1E and S1D) with research grade Treg enrichment yielding approximately 3.75–7.5 × 10^7 CD4$^+$ T cells per 50 mL blood (Figure 1F). Satisfying yields of Treg were reached independently from donor age and comparable to other studies (Figures S1B and S1C). Gene editing enhanced the cell proliferation rate in the presence of IL-2, anti-CD3/CD28 microbeads and rapamycin (Figure 1G), yielding a greater number of FKBP12^{KO}-Tregs compared to non-edited Treg (Figures 1G and S1E–S1H) >400 × 10^8 cells, sufficient for clinical application if applying 3 × 10^6 cells/kg bodyweight and including material for controls[13,14] (Figures S1B and S1C). High-fidelity Cas9 editing achieved approximately 60% and standard Cas9 enzyme 80% efficiency in the final FKBP12^{KO}-Treg product expanded for 21 days in comparison to MOCK controls (Figure 1H) and Treg^{WT} (Figure S1F) with negligible editing frequencies.[19] Viable cell recovery and proliferation were enhanced in FKBP12^{KO}-Tregs compared to Treg^{WT}, while mean fluorescence intensity (MFI) of CD25 and FOXP3 was shown to be donor dependent (Figures S1G and S1H).

FKBP12^{KO}-Treg quality is comparable with Treg^{WT}

The FKBP12^{KO}-Treg, Treg^{WT}, and non-Treg CD4$^+$ T cells (negative sort fraction), were subjected to detailed analysis following expansion in identical conditions. The Treg markers; FOXP3 and CD25 were highly expressed by Treg^{WT}, FKBP12^{KO}-Treg, MOCK and Tcon (Figures 2A and 2B). However, the MFI of FOXP3 was higher in Treg *vs.* Tcon and comparable between FKBP12^{KO}-Treg and Treg^{WT} (Figure 2C). Interestingly, CD25 MFI was lower among the FKBP12^{KO}-Treg compared to MOCK/Treg^{WT}/Tcon (Figure 2D). After phorbol 12 myristate 13 acetate (PMA)/ionomycin stimulation, the effector cytokines IL-2 (Figures 2E and 2F), and tumor necrosis factor α (TNF-α) (Figure 2G) were produced at very low levels, medians of 1% and 2%, respectively, among Treg^{WT} and FKBP12^{KO}-Treg

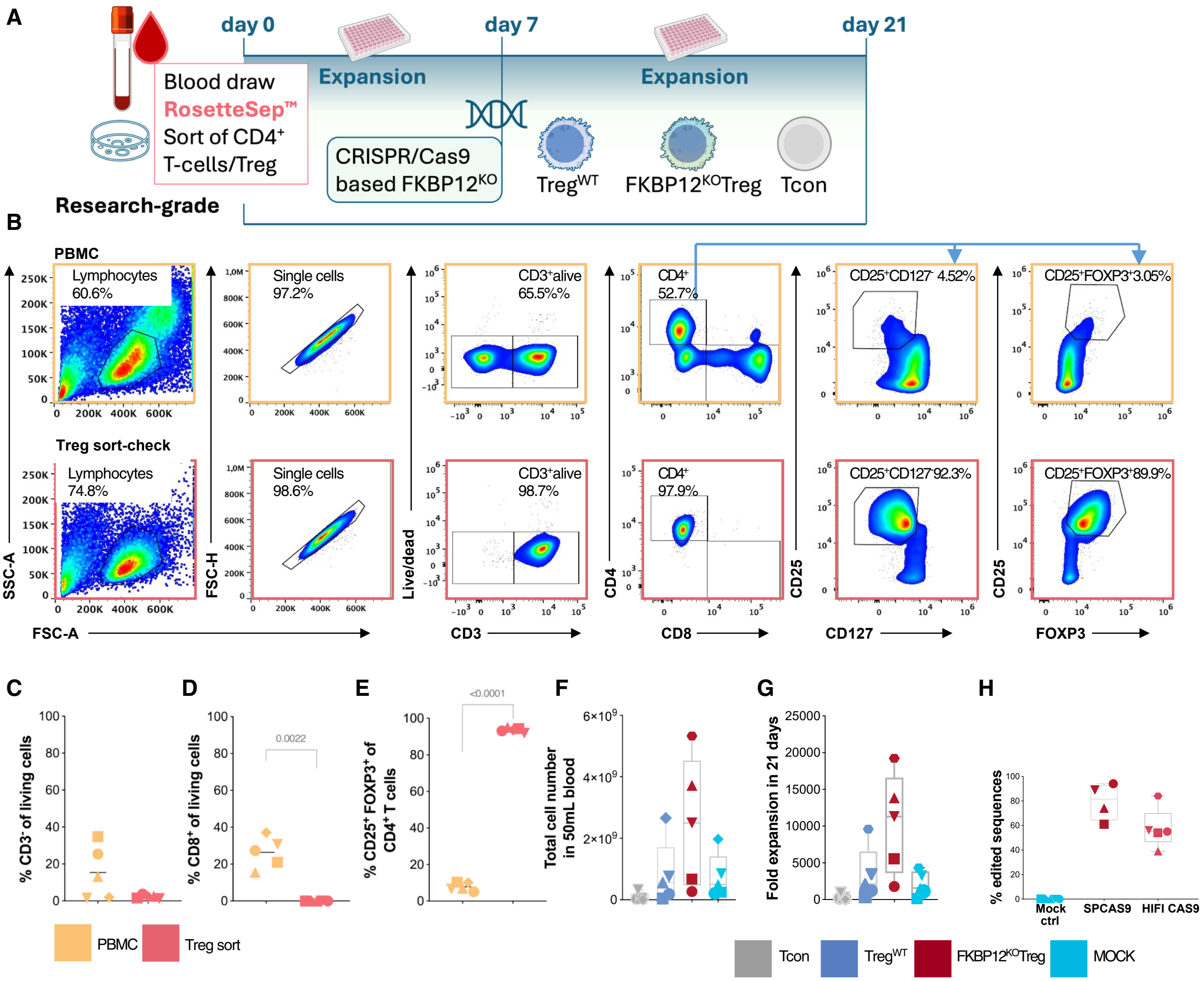

Figure 1. FKBP12^{KO}-Treg are pure and show improved expansion

(A) A description of the Treg product production process. NIH Bioart source was used. (B) Representative dot plots show the gating strategy and phenotype of PBMCs before and after Treg sorting. For the following subfigures, each symbol represents data from an individual donor. Pre-sort (yellow) post-sort (light red). Conventional T cells (Tcon) (gray), Treg^{WT} (blue), FKBP12^{KO}-Treg (red), and MOCK (turquoise) unless stated otherwise. Paired *t* test and RM one-way ANOVA were used, $n = 5$. (C) Percentage of CD3^{-} T cell subset before and after sorting. (D) Percentage of CD8^{+} T cells before and after sorting. (E) Percentage of CD4^{+}CD25^{+}FOXP3^{+} T cells before and after sorting. (F) The total number of cells obtained from 50 mL of blood is shown after 21 days of expansion. (G) Fold expansion from day 0–21. Boxplots with whiskers indicate extreme values, $n = 5$. (H) Analysis of the KO efficiency of Treg after a 3-weeks expansion period, performed using Sanger sequencing of FKBP12 PCR products. Sequencing data were analyzed using the Inference of CRISPR edits (ICE) tool (Synthego). Electroporation with Cas9 without sgRNA/MOCK electroporated (turquoise), with classical *Staphylococcus pyogenes* Cas9 (red) and with a high-fidelity *Staphylococcus pyogenes* Cas9 (light red).

compared with 15% and 40% among the CD4^{+} Tcon expanded in parallel. The median frequencies of producers of interferon (IFN)-γ were determined to be 10% for Tcon; while 3% vs. 2% for FKBP12^{KO}-Treg and Treg^{WT}, respectively (Figures 2E–2H). Gene expression levels of IFNγ determined by cellular indexing of transcriptomes and epitope sequencing (CITE-seq) were shown to be elevated in FKBP12^{KO}-Treg in comparison to Treg^{WT}, whereas no skewing toward increased TBX21 or NFKB1/2 could be shown under tacrolimus (Figure S2G). Clonality determined by T cell receptor β (TCR-β) sequencing was found to be similar for FKBP12^{KO}-Treg and Treg^{WT} (Figure 2I). Although frequencies of CD137 expressing cells were similar among FKBP12^{KO}-Treg and Treg^{WT}, they were less frequent among Tcon (Figures S2A and S2B). Frequencies of cells expressing CD40L (CD154) were higher among Tcon than both Treg populations (Figures S2A and S2C). The TSDR methylation of the Tcon was found to be 85%, while it was and <20% among the FKBP12^{KO}-Treg, MOCK, and Treg^{WT} (Figure 2J). Treg suppressed responder T cell proliferation (Tresp) in a dose-dependent manner (Figures 2K and 2L). Due

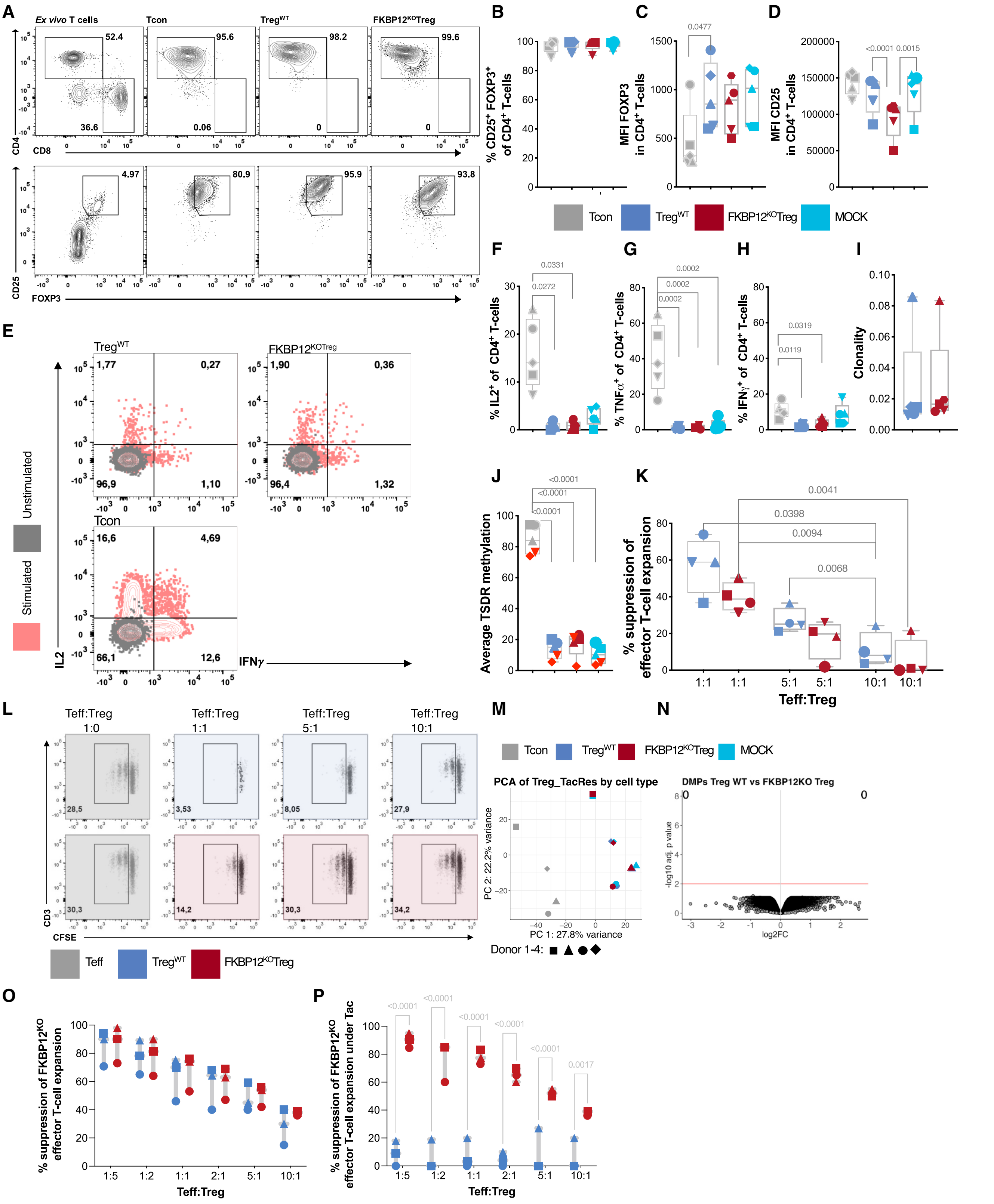

(legend on next page)

to the influence of immunosuppressants on Tresp proliferation, we tested FKBP12^{KO}-Treg suppression on FKBP12^{KO}-Tresp and could show significantly increased suppressive capacity under Tacrolimus treatment in comparison to Treg^{WT} (Figures 2O and 2P). Suppressive capacity on FKBP12^{KO}-Tresp by FKBP12^{KO}-Treg was increased under everolimus treatment but not under prednisolone treatment (Figures S2D and S2E). FKBP12^{KO}-Treg suppressive capacity under CsA treatment was not depicted since CsA largely inhibited FKBP12^{KO}-Teff expansion in general, whereas the inhibition was less prominent and allowed analysis for the other immunosuppressants (Figure S2F).

FKBP12^{KO}-Treg and Treg^{WT} have similar epigenetic profiles

FKBP12^{KO}-Treg, Treg^{WT}, and Tcon were subjected to an EPIC (850k array) and revealed distinct Treg and Tcon clusters in PC1 (Figure 2M). Furthermore, there was no significantly differentially methylated position when comparing FKBP12^{KO}-Treg with Treg^{WT} and differentially methylated positions were not of known relevance on Treg biology e.g., olfactory transduction (Figures 2N and S1K).

FKBP12^{KO}-Treg exhibit enhanced characteristics when exposed to tacrolimus

FKBP12^{KO} conferred proliferative advantage over Treg^{WT} when exposed to tacrolimus, which was not observed under CsA and under standard triple immunosuppression treatment using a cocktail of tacrolimus, prednisolone, and mycophenolic acid (Figures 3A, 3B, S1I, and S1J). However, Treg^{WT} proliferation was inhibited by all immunosuppressants including tacrolimus (Figures 3A, 3B, S1I, and S1J). Furthermore, the expression of cytotoxic T-lymphocyte-associated protein (CTLA)-4 was increased in FKBP12^{KO}-Treg vs. Treg^{WT} under CsA and tacrolimus (Figures 3C and 3E). Tacrolimus-treated FKBP12^{KO}-Treg exhibited higher programmed death (PD-)1 MFI in comparison to Treg^{WT} (Figures 3C and 3D). Additionally, Treg^{WT}'s CTLA-4 MFI was diminished relative to X-VIVO medium controls in presence of both CNIs but not in FKBP12^{KO}-Treg treated with tacrolimus (Figure 3E). Moreover, knocking out FKBP12 resulted in downregulation of the FKBP1A gene, underlining knockout efficiency and further affected regulation of a number of other proteins (Figures 3F–3M); (1) increased metabolic arginase-2 (FOXP3 stability), (2) decreased NIT2 levels (histidine metabolism) in comparison to Treg^{WT}, (3) significantly, upregulated Guanylate binding protein-2 (Treg-suppressive capacity), and (4) downregulated zyxin (stress fiber protein) when exposed to tacrolimus.[20] Pathway analysis identified differentially regulated proteins in FKBP12^{KO}−Treg and Treg^{WT} highlighting increased IL-2 and IL-10 signaling as well as regulation of TNFR signals. Furthermore, IFN-γ signaling was decreased in FKBP12^{KO}-Treg compared to Treg^{WT} (Figure 3N). Detailed protein characterization regarding proliferation related and metabolic signatures revealed no discernable differences between FKBP12^{KO}-Treg and Treg^{WT} under the immunosuppressive regiments tested; no signs of increased exhaustion, cellular stress, or dysfunction could be shown on protein level (Figure S5A). When investigating regulatory/metabolic protein signatures, it was apparent that FKBP12^{KO}-Treg under Tacrolimus tended to upregulate lactate dehydrogenase A (LDHA) and Phosphoglycerate Mutase (PGM)1 (Figures S5A–S5C).[21–24] Furthermore, FKBP12^{KO}-Treg in comparison to Treg^{WT} under tacrolimus tended to increase Glucose-6-phosphate isomerase (G6PI) and Coronin (COR)1A and decrease Ubiquinol-Cytochrome C Reductase Core Protein (QCR)2/6 and Peptidylprolyl isomerase A (PPIA), however none of the changes were statistically significant (Figure S5C).

FKBP12^{KO}-Treg can be produced under pre-GMP conditions

We adapted the production process for FKBP12^{KO}-Treg to pre-GMP conditions using CliniMACS Plus to isolate CD4^{+} cells from whole blood (Figure 4A), which resulted in a product with <0.002% CD8^{+} cell contamination Figures 4B and 4C). Afterward, it was possible to isolate CD25^{+}FOXP3^{+} cells with >85% purity among CD4^{+} cells (Figures 4D and 4E). Following expansion, pre-GMP FKBP12^{KO}-Treg and Treg^{WT} had comparable FOXP3 MFI, but FKBP12^{KO}-Treg showed significantly lower CD25 MFI than Tcon (Figures 4F and 4G) with negligible changes in effector cytokine expression between FKBP12^{KO}-Treg and Treg^{WT} (Figures 4H–4K). TSDR methylation levels were lower in both Treg products compared to Tcon (Figure 4L).

Pre-GMP FKBP12^{KO}-Treg mirror enhanced characteristics of research grade FKBP12^{KO}-Treg

To test the FKBP12^{KO}-Treg expansion in comparison to Treg^{WT}, we expanded both in X-VIVO culture media containing CNIs + IL-2 for 10 days. FKBP12^{KO}-Treg proliferation was superior in comparison to Treg^{WT} in the presence of tacrolimus, but both showed sensitivity to CsA (Figure 5A). Both products maintained FOXP3 expression in contrast to Tcon (Figure 5B). Significantly, the CD25 MFI variance

Figure 2. FKBP12^{KO}-Treg phenotype and function is comparable to Treg^{WT}

Each symbol represents data from an individual donor. Conventional T cells (Tcon) (gray), Treg^{WT} (blue), and FKBP12^{KO}-Treg (red). Paired *t* test and RM one-way ANOVA were used *n* = 5. (A) Representative dot plot showing the gating strategy for living T cells, CD4^{+}, CD8^{+}, and CD25^{+}FOXP3. (B) The percentage of CD4^{+}CD25^{+}FOXP3^{+} T cells on day 21. (C) MFI of FOXP3 at day 21. (D) MFI of CD25 at day 21. (E) Representative dot plots for the producers of IL-2 and IFN-γ, unstimulated (gray) and stimulated (pink). (F) Summary of the percentage of IL-2 producers (G) TNF-α producers (H) IFN-γ producers after stimulation. (I) Comparison of FKBP12^{KO}-Treg clonality with Treg^{WT} using TCRb sequencing. (J) TSDR demethylation at the promoter of FOXP3 of DNA collected on day 21: Female donors highlighted in light red, due to availability of two X chromosomes. (K) The percentage of Treg suppressive capacity on CFSE labeled autologous fresh CD3^{+} T cells stimulated with CD3/CD28 beads. (L) Representative dot plots and gating of proliferated responder T cells (Teff) co-cultured with Treg at different Teff:Treg ratios and stimulated 1:1 with anti-CD3/CD28-coated microbeads. (M) Principal-componant analysis (PCA) of data determined in EPIC arrays showing clustering for Tcon (gray) vs. all Treg populations, but no clustering for Treg^{WT} (blue), MOCK (turquoise), and FKBP12^{KO}-Treg (red). (N) Volcano plot displaying differentially methylated positions (DMPs) within Treg^{WT} vs. FKBP12^{KO}-treg. There were no significant DMPs at a significance level of FDR = 0.01 (red line). (O) Frequency of suppression of FKBP12^{KO}-Teff in medium control, FKBP12^{KO}-Treg (red), Treg^{WT} (blue), *n* = 3 for different ratios Teff:Treg ratios. (P) Showing percentage of suppression under tacrolimus (Tac) with same color coding, *n* = 3.

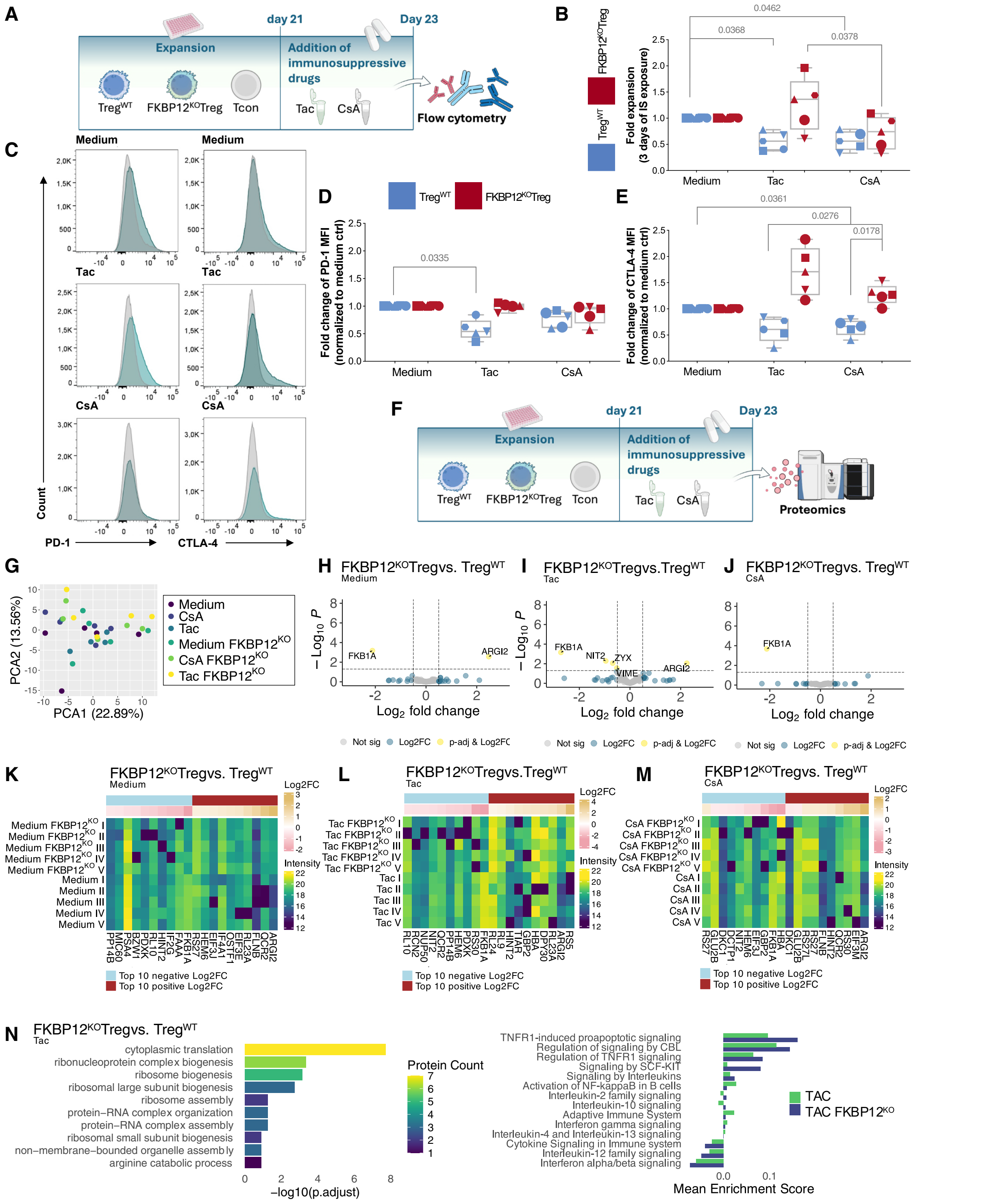

Figure 3. FKBP12^{KO}-Treg show preferable characteristics under tacrolimus

Each symbol represents data from an individual donor. Tacrolimus was used at 6 ng/mL and cyclosporine (CsA) at 120 ng/mL with Treg^{WT} (blue), FKBP12^{KO}-Treg (teal). Paired *t* test and RM one-way ANOVA were used, $n = 5$. (A) Schematic overview of Treg expansion in the presence of indicated immunosuppression. NIH Bioart source was used. (B) Treg products were exposed to the indicated immunosuppression for a total of 3 days after 3-weeks of manufacturing. Data normalization using Remove baseline and column math' function of GraphPad Prism. (C) Histograms of CTLA-4 and PD-1 staining of Treg^{WT} (gray) and FKBP12^{KO}-Treg products (green) exposed to indicated

(legend continued on next page)

between FKBP12^{KO}-Treg and Treg^{WT} was not observed after 10 days exposure to tacrolimus (Figure 5C). DigiWest-based proteome analysis confirmed upregulation of the proteins identified in mass spectrometry analysis in FKBP12^{KO}-Treg vs. Treg^{WT} (Figures 5D, 5I, 5K, and 5M).[25] Reactome pathway analysis showed enrichment of ribosomal processes correlating with single upregulated proteins in FKBP12^{KO}-Treg vs. Treg^{WT} (Figure 3N). Furthermore, FKBP12^{KO}-Treg showed upregulation of IL-2, tumor necrosis factor receptor (TNFR-)1, and IL-10 family signaling as well as increased downregulation of pro-inflammatory cytokines, especially IFN-γ, IL-12/IL-13/IL-4, and IFN-α/β family signaling under tacrolimus treatment (Figure 3N). Interestingly, CD4 was upregulated and CD8a was downregulated in tacrolimus-treated FKBP12^{KO}-Treg vs. Treg^{WT} (Figure 5D). To get insights on CD8/CD4 changes in FKBP12^{KO}-Teff, a dataset of cytomegalovirus (CMV)-antigen specific T cells was incorporated from a distinct proteomics run showing that CD4 and CD8 were not differentially regulated in FKBP12^{KO}-Teff counterparts (Figure S5D). *CTLA4* gene expression in FKBP12^{KO}-Treg increased under tacrolimus (Figures S4C and S4E). Moreover, we observed increased CD25 (IL2Rα) and phospho-extracellular signal-regulated kinase (ERK) (Erk1/2/Thr202/Tyr204) expression in tacrolimus-treated FKBP12^{KO}-Treg vs. Treg^{WT} (Figure 5D). These findings align with increased levels of activation markers, e.g., Glucocorticoid-induced TNFR-related protein (GITR) and lymphocyte-activation gene (LAG-)3.[26,27] Signal Transducers and Activators of Transcription (STAT-)5 (Figure 5D) and pSTAT5 (Figure S4B) protein were not differentially regulated in FKBP12^{KO}-Treg vs. Treg^{WT} upon exposure to tacrolimus.

Cellular indexing of transcriptomes and epitope sequencing confirms pre-GMP FKBP12^{KO}-Treg's functionality

Using Cellular Indexing of transcriptomes and epitope sequencing (CITE-seq) data, 14 cell clusters were annotated, which were shared between FKBP12^{KO}-Treg and Treg^{WT} but distinct from the Tcon following (re)stimulation (Figures 6A–6C and S3A).[18] The cluster composition of both FKBP12^{KO}-Treg and Treg^{WT} changed in the presence of CNIs mainly regarding cluster 11 and 14 (Figure S3A). However, FKBP12^{KO}-Treg was not affected by the CNI tacrolimus (Figure 6C).

We compared FKBP12^{KO} and Treg^{WT} with respect to expression of key genes (Figure 6D). Compared with Treg^{WT}, FKBP12^{KO} showed an increase in T helper 2 (TH2) signature e.g., *IL-5* upregulation in control medium and tacrolimus conditions, while under CsA treatment TH2 signature was reduced (Figures 6D and 6E). Kyoto Encyclopedia of Genes and Genomes (KEGG) and Gene Ontology (GO)-term highlighted TH2 signaling, regulation of *IL-10* and B cell activation pathways ($p > 0.005$, Table S4). Furthermore, *LAG3*, *STAT5*, *IL10*, and Tumor Necrosis Factor Receptor Superfamily (*TNFRSF*)*-1B* gene and protein expression levels were upregulated in FKBP12^{KO}-Treg vs. Treg^{WT} especially when exposed to tacrolimus (Figures 5D, 6E, 6F, and 6I). *IL2RA* gene expression was slightly reduced in PMA-ionomycin stimulated FKBP12^{KO}-Treg compared to Treg^{WT}. To get insights on *IL2RA* changes in FKBP12^{KO}-Teff, a dataset of CMV peptide-loaded lymphoblastic cell line stimulated CMV-antigen specific T cells was incorporated from a distinct single cell run and *IL2RA* changes were shown to be even more pronounced in antigen-specifically stimulated FKBP12^{KO}-Teff vs. Teff^{WT}, under medium conditions, which was reversed under tacrolimus (Figures S2G and S2H). Unfortunately, a head-to-head comparison of FKBP12^{KO}-Treg and -Teff datasets was not possible due to differences in data scaling and experimental conditions. In general, relevant immune check-point markers, e.g., *TNFRSF1B* and *PDCD1* (PD-1), were increased in tacrolimus treated FKBP12^{KO}-Treg. In addition, tacrolimus treated FKBP12^{KO}-Treg showed higher *PDCD1* levels and higher *TIGIT* levels on gene level but not on surface level shown by T cell immunoreceptor with immunoglobulin and immunoreceptor tyrosine-based inhibitory motif domains (TIGIT) MFI compared to Treg^{WT} under tacrolimus and everolimus (Figures 6D and S4A). *CTLA4* and *CD73* gene and surface protein expression levels were upregulated and preserved under tacrolimus (Figures S4C–S4E). To examine potential exhaustive features of FKBP12^{KO}-Treg in comparison to Treg^{WT} that could potentially be induced by high proliferation rates, we showed that negatively described markers for Treg longevity, such as Killer Cell Lectin-Like Receptor Subfamily G Member (*KLRG*)-1, Thymocyte Selection-Associated High Mobility Group Box (*TOX*), and B cell lymphoma (*BCL*)-2 were reduced in both FKBP12^{KO}-Treg and Treg^{WT} on gene level (Figures 6G and 6H). FKBP12^{KO}-Treg showed a stable gene expression of *IL2RA* and FOXP3 as well as balanced proliferation marker gene expression e.g., for *STAT5A/B*, *MYC*, Solute carrier family (*SLC*)-2A, Heterogeneous nuclear ribonucleoprotein K-like protein *(HEK)* 2, and cyclin-D (*CCND*)-2 under tacrolimus treatment (Figure 6G). Interestingly, IL-2 dependency of FKBP12^{KO}-Treg was shown to be more focused on *IL2RA/B* but not *IL2RG* with increased levels of Janus kinase (*JAK*)*3* and

immunosuppression. (D) Flow cytometry analysis showing the fold change of MFI of PD-1 in Treg^{WT} and FKBP12^{KO}-Treg and (E) CTLA-4 after exposure to the indicated immunosuppression, data normalization using Remove baseline and column math' function of GraphPad Prism. (F) Schematic overview of proteomics approach. NIH Bioart source was used. (G) PCA of Treg^{WT} and FKBP12^{KO} samples under medium control conditions or following treatment with CsA or Tac. (H–J) Differential expression analysis comparing FKBP12^{KO}-Treg versus Treg^{WT} under medium control conditions and following Tac or CsA treatment. Volcano plots display -log10 adjusted *p* values (*y* axis) versus log2 fold change (Log2FC, *x* axis). Proteins with Log2FC > 0.5 are highlighted in blue; proteins with Log2FC > 0.5 and adjusted *p* value <0.05 are highlighted in yellow. (K–M) Heatmaps displaying intensity levels of the top 10 proteins with highest positive Log2FC and top 10 proteins with highest negative Log2FC comparing FKBP12^{KO}-Treg versus Treg^{WT} under medium control conditions and following Tac or CsA treatment. (N) Left: Gene Ontology (GO) pathway enrichment analysis showing the top 10 enriched pathways comparing FKBP12^{KO}-Treg versus Treg^{WT} following tacrolimus treatment. The -log10 adjusted *p* value is displayed, with the number of enriched proteins in each pathway indicated by color coding. Right: single-sample Gene Set Enrichment Analysis (ssGSEA) of FKBP12^{KO}-Treg and Treg^{WT} following Tac treatment using the Reactome database. Mean enrichment score of selected pathways is shown.

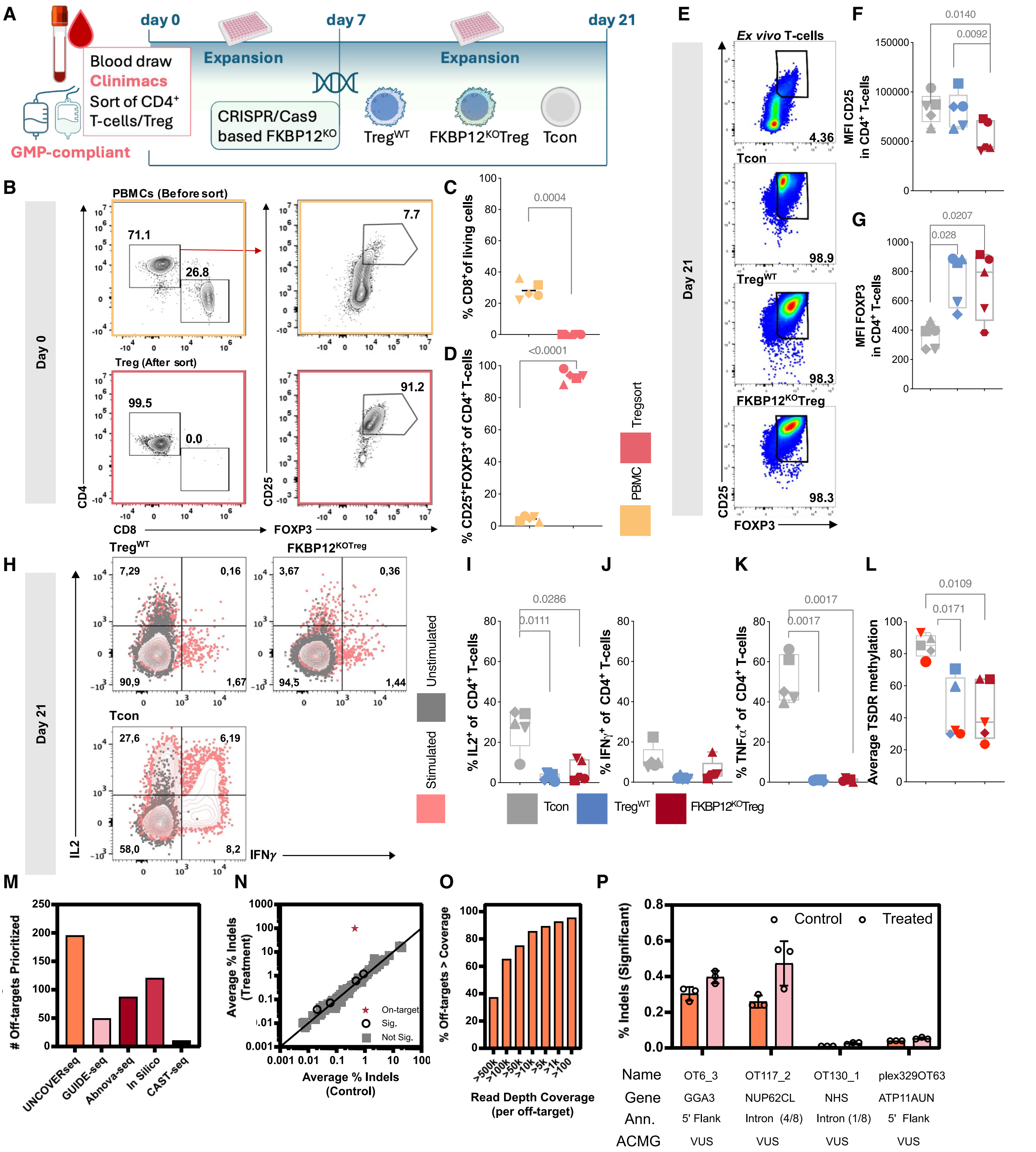

Figure 4. FKBP12^{KO}-Treg can be manufactured in a GMP compatible manner ("pre-GMP")

Each symbol represents data from an individual donor. Pre-sort (yellow) post-sort (light red). Conventional T cells Tcon) (gray), Treg^{WT} (blue), FKBP12^{KO}-Treg (red), paired *t* test, and RM one-way ANOVA were used, *n* = 5. (A) A description of the production process. NIH Bioart source was used. (B) Representative dot plots and gating for CD4^{+}, CD8^{+}, and CD25^{+}FOXP3^{+} of living T cells before and after sorting. (C) Percentage of CD8^{+} of living T cells before and after sorting. (D) Percentage of CD25^{+}FOXP3^{+} CD4^{+} T cells before and after sorting. (E) A representative dot plot showing percentages of CD25^{+}FOXP3^{+} T cells in *ex vivo* T cells, Tcon, Treg^{WT}, and FKBP12^{KO}-Treg on day 21.

(legend continued on next page)

Phosphatase and Tensin homolog (*PTEN*) gene expression and less reliance on Phosphoinositide 3-kinase (*PI3K*) dependent gene expression under tacrolimus treatment (Figure S4F). In addition, inducible T-cell co-Stimulator (*ICOS*) gene expression and related genes were increased in FKBP12^{KO}-Treg under tacrolimus treatment, while in FKBP12^{KO}-Teff this increase was less prominent (Figure S4G, S4H, and S4J).

GMP process validation

Based on the research grade and pre-GMP data, we decided to develop GMP manufacturing using semi-closed bioreactors for expansion and the newly established isolation combining CliniMACS with Tyto sorting and gene-editing processes described previously. Indeed, we succeeded in producing stable “TregTacRes” products with >94% viable cells, being >95% $CD4^+CD25^+FoxP3^+$, with contaminations of less than 0.3% $CD8^+$ T cells, <6% IL-2, and IFN-γ producers, including >79% of FKBP12^{KO}-Treg (Table 1). After isolation, we yielded a mean of 0.65 × 10^6 Treg, ending after d18-23 with a 21.305-fold expansion rate. Validation of the in house digital droplet polymerase chain reaction (ddPCR) established to determine KO-efficacy in FKBP12^{KO}-Treg was implemented as qualified method for GMP manufacturing showing validated protocol results in comparison to non-validated results and Sanger sequencing (Figures 7A and 7B, and Tables 1 and S6). Based on these and other data, e.g., sterility testing according to the European Pharmacopoeia, the local authority granted us manufacturing authorization for the “TregTacRes” manufacturing process (Figure 7C) on May 16th, 2024.

Off-target nomination

We performed chromosomal aberrations analysis by single targeted ligation- mediated PCR-Sequencing (CAST-Seq), Abnoba-seq, CRISPR Off-target Sites with Mismatches, Insertions, and Deletions (COSMID) off-target search, *in silico* population-based nominations using a tool developed by Integrated DNA Technologies (IDT) and Unbiased Nomination of CRISPR Off-target Variants using Enhanced RNAse-H dependent PCR Sequencing (UNCOVERseq) to nominate potential off-targets hit by the sgRNA (Table S5 and Figure 4M). Nomination of off-targets using UNCOVERseq in a promiscuous nomination system (HEK293-Cas9) discovered 1 on-target and 2,568 off-targets significantly enriched (p_{adj} < 0.05) in comparison to unedited samples. Of these off-targets, 182 met tier 1 to tier 3 criteria for prioritization, and 133 of these were reproduced between 2 or more biological replicates (Table S5). Reproducibly nominated off-targets across donors occurred in the following genomic regions: 9.7% exonic (13/133), 45.8% intronic (61/133) and the remaining sites in intergenic or atypical genomic context (Table S5). Sites were prioritized from each method based on annotation risk, reproducibility (intra- and inter-method), frequency of nomination, and similarity to the gRNA (Levenshtein distance). This led to prioritization of 195 UNCOVERseq sites, 10 CAST-seq sites, 70 COSMID and 25 Abnoba nominated sites (both combined in Figure 4M), and 120 *in silico* nominated sites (from both reference and population genomes) and *in silico* nomination identified an additional 103 unique sites (Figure 4M). After deduplication of sites nominated by multiple methods, this led to a total of 255 putative off-target sites being prioritized for off-target confirmation (Table S5).

Off-target confirmation

Interrogation of the 255 prioritized off-targets was performed using deep-sequencing via an amplicon sequencing approach. Three donors (donor 1, donor 3, and donor 4) were transfected in biological singleton and compared as replicates to confirm significant donor independent editing. Following sequencing, a median read depth of 201,000×–445,000× per off-target was achieved (Figure S6A). 85.5% of off-targets were quantified as having greater than 10,000× coverage, and 95.3% having greater than 100× coverage (Figure 4O). Upon quantification of control samples, a variable background indel rate was observed with a median of 0.12% and an interquartile range of 0.07%–0.29% indels (Figure S6B). We compared this to previously published frequencies from 263 unique gRNAs and found it was elevated in comparison to what has been previously reported in control samples. We hypothesize this may be due to homopolymer repeats in the on/off-target sequences of the FKBP12 gRNA, which is a known motif for heightened error rates from Illumina sequencing.[28]

Interrogation of on-target editing confirmed high frequencies of on-target editing ranging from 97%–99% (Figure 4N). Off-target editing analysis identified four sites that were found to have significant editing (Figures 4N and 4P). One site was identified in two overlapping annotations of the 5′ flanking region of the *GGA3* gene and a non-coding region (intron 1 of 4) of the Mitochondrial Ribosomal Protein S (*MRPS*)-7 gene with a significant indel frequency ranging from 0.36%–0.43% (Figure 4P). Two edited sites were in intronic regions of the Nucleoporin 62 C-Terminal Like (*NUP62CL*) and Nance-Horan syndrome (*NHS*) genes with significant indel frequencies ranging from 0.33%–0.54% and 0.02%–0.03%, respectively (Figure 4P). The final significant off-target was identified in non-coding 5′ flanking region of the ATPase phospholipid transporting

(F) MFI of CD25 on day 21. (G) MFI of FOXP3 on day 21. (H) (I–K) Representative dot plots of producers of IL-2 and IFN-γ following 6 h stimulation by PMA/ionomycin and intracellular capture of cytokines by addition of brefeldin A after 45 min. Unstimulated (gray), stimulated (pink) with (I) corresponding summary of the percentage of IL-2 producers (J)Summary of the percentage of TNF-α, and (K) IFN-γ producers. (L) Methylation of the TSDR at the promoter of FOXP3 of DNA collected on day 21. Female donors are highlighted in light red. Off-target safety assessment (M–P) with (M) showing off-target nomination from multiple different empirical and in silico methods and the # of prioritized sites per method. (N) Editing quantification is shown for assays with >1,000× coverage. Each dot represents the average raw frequency of indels of the control (*x* axis) and treatment (*y* axis) plotted. Red dots indicate sites with statistically significant differences (p adj<0.05), gray indicates not significant sites, and a red star indicates the on-target site. (O) Prioritized off-targets were sequenced and the # of targets meeting variable coverage thresholds quantified. (P) Significant events have individual donor frequencies shown for enriched indel events and genomic annotations in addition to ACMG classification of -1 bp deletion 5′ the annotated cut site.

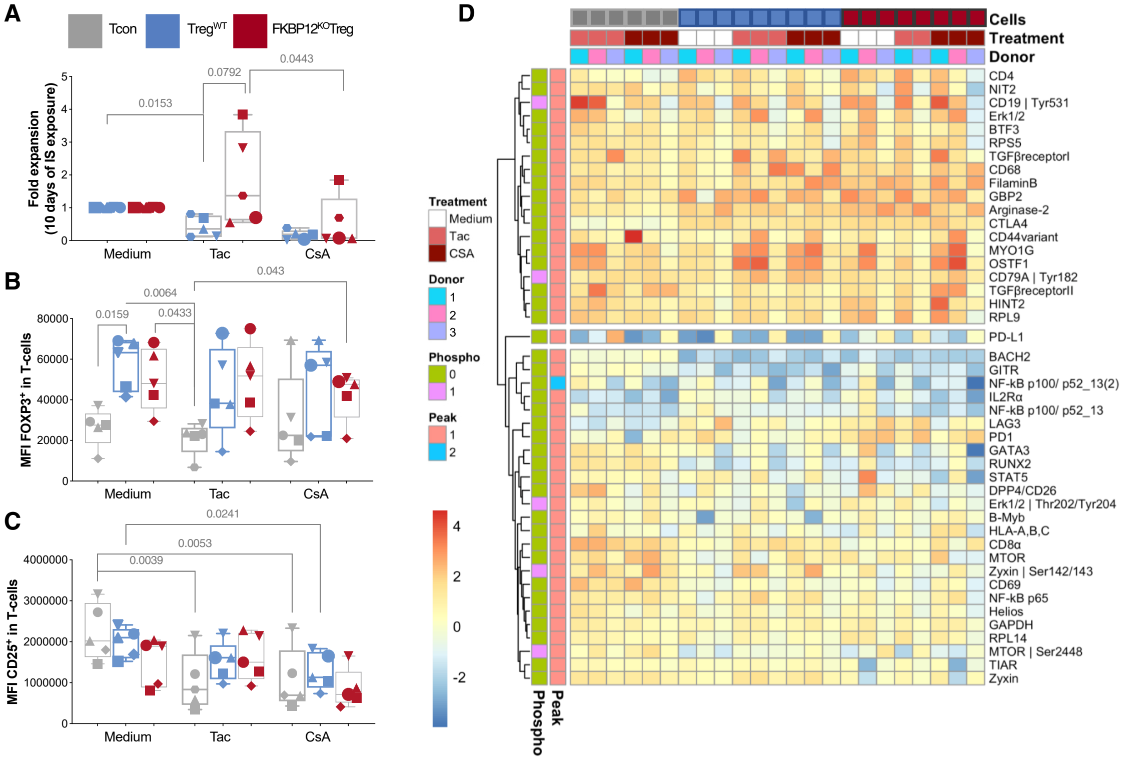

Figure 5. Characterization of Treg response to immunosuppression: impact of "pre-GMP" FKBP12^{KO}-Treg

Each symbol represents data from an individual donor. Treg products were exposed to 6 ng/mL tacrolimus or 120 ng/mL CsA for 10 days, immunosuppressants were added and re-stimulation was performed every 2nd day. Conventional T cells (Tcon, gray), Treg^{WT} (blue), and FKBP12^{KO}-Treg (red). Paired *t* test and RM one-way ANOVA were used, *n* = 5. (A) Expansion of Treg products in presence of immunosuppressants, data normalization was performed using remove baseline and column math' function of GraphPad Prism. (B) MFI of FOXP3 of living T cells and (C) MFI of CD25 is shown for all conditions. (D) The heatmap depicts the differentially expressed proteins of expanded Tcon, Treg^{WT}, and FKBP12^{KO}-Treg exposed to Tacrolimus or CsA medium control and measured by DigiWest technology.

(*ATP*)11A gene with significant indel frequencies ranging from 0.05%–0.06% (Figure 4P). Variant annotation of a 1 bp deletion at the cut site results in variants of unknown significance (VUS) via American College of Medical Genetics and Genomics (ACMG) classification criteria for all identified off-targets (Figure 4P).

DISCUSSION

Drugs like tacrolimus revolutionized allogeneic transplantation of organs regulating immune responses artificially.[29] In contrast, Treg are "living drugs" that enable endogenous, regenerative immunosuppression without toxicities.[30] A major limitation of adoptive Treg therapy in transplantation is its incompatibility with CNI-based immunosuppression, while tacrolimus is needed to effectively suppress Teff. Using CRISPR-Cas9-mediated disruption of FKBP1A, we generated tacrolimus-resistant FKBP12^{KO}-Tregs and subjected them to extensive phenotypic, functional, molecular, and safety characterization. Across research-grade, pre-GMP, and GMP conditions, FKBP12^{KO}-Tregs consistently retained defining features of stable human Tregs, including high FOXP3 expression and a demethylated TSDR, comparable to unedited TregWT and clearly distinct from activated conventional T cells. Importantly, FKBP1A deletion did not induce aberrant activation, loss of regulatory identity, or clonal skewing, indicating that resistance to tacrolimus can be achieved without destabilizing the Treg lineage. Off-target analysis revealed 4 significant off-targets; however those were shown to be of no biological relevance to Tregs. We suggest FKBP12^{KO}-Tregs will significantly enhance *in vivo* functionality, efficacy, and longevity in patients prescribed with tacrolimus.

Manufacturing and performance

From a translational perspective, it was critical to demonstrate that these biological properties could be maintained under clinically relevant manufacturing conditions. We were able to generate a pure FKBP12^{KO}-Treg product from 50 mL of whole blood with sufficient cell numbers for clinical trials applying e.g., up to 3×10^6 Treg/kg.[14,31,32] In context to published literature, achieved cell yields comparable to the ones successfully used in kidney transplant patients, across different age groups and under immunosuppression.[33–35] It is to be highlighted, that the publications cited used various approaches of either leukapheresis or at least 10 times more blood volume to generate sufficient numbers of TregTacRes for clinical application. Up to now, manufacturing Treg from 40 to

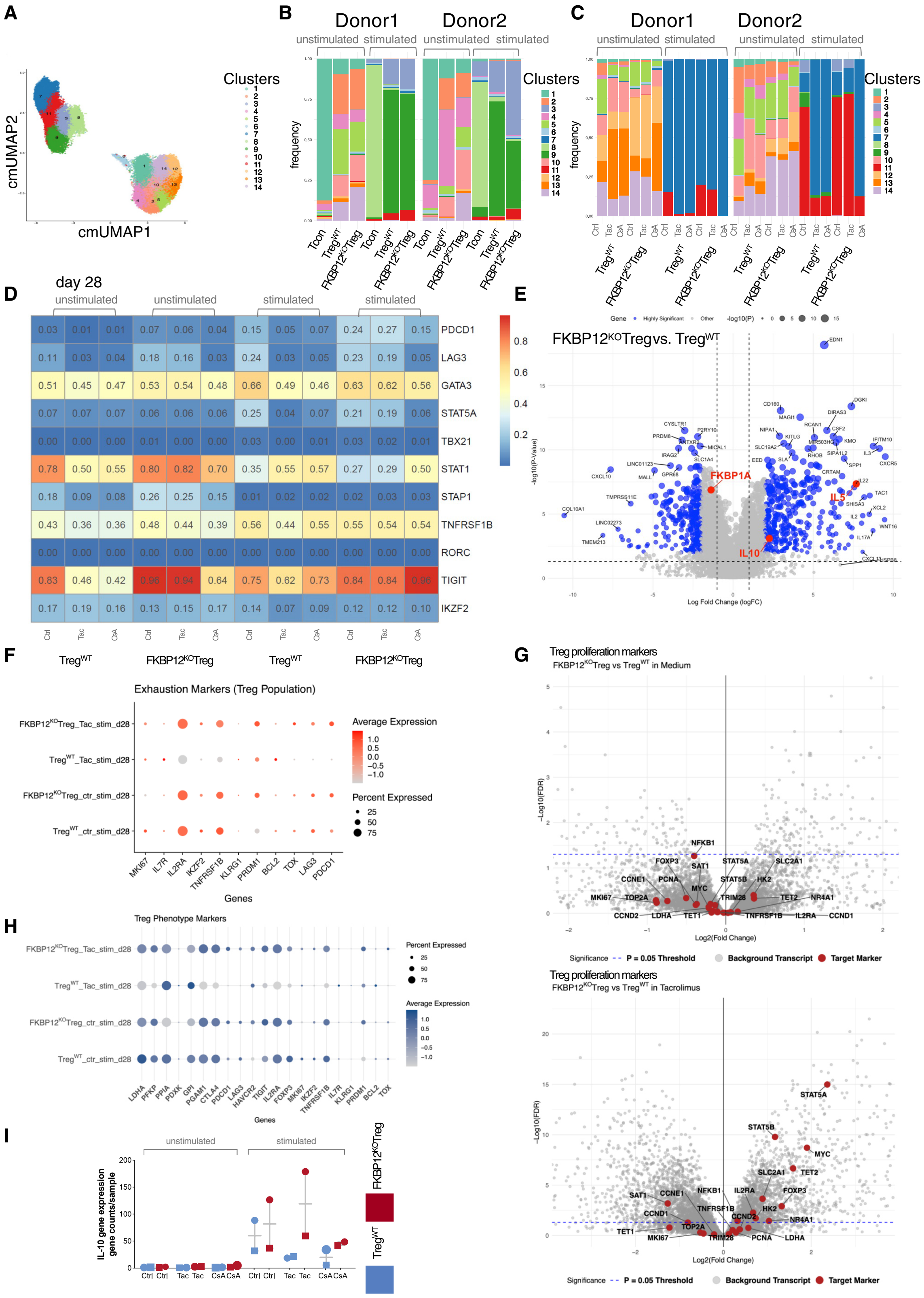

(legend on next page)

50 mL of whole blood reaching sufficient numbers was only managed at our center and within collaborative efforts under the umbrella of the ONE Study.[13,14,36] Functionally, manufactured FKBP12^{KO}-Tregs displayed the key intended phenotype: preserved proliferation, signaling, and regulatory marker expression in the presence of tacrolimus, in contrast to Treg^{WT}. At the same time, FKBP12^{KO}-Tregs remained sensitive to CsA, providing an important clinical safeguard should immunosuppression need to be adjusted.[32,37] The observed proliferative advantage of FKBP12^{KO}-Tregs under IL-2 and rapamycin exposure is consistent with the known dependence of rapamycin-mediated growth inhibition *via* FKBP12[38–41] and did not translate into loss of regulatory phenotype or function. Excluding any potential gene-edited Tcon impurities with proliferative advantage,[42–44] we observed reduced CD8a protein levels in FKBP12^{KO}-Treg vs. Treg^{WT}. Consistent with published data on general FOXP3 upregulation in Treg as well as activated CD4^{+} T cells, we show that FOXP3 was upregulated in activated Tcon, however the MFI was diminished in comparison to Treg.[19,45,46] The TSDR methylation levels were similar in Treg^{WT} and FKBP12^{KO}-Treg and no significantly different demethylated positions were shown, while Tcon showed increased methylation, thus confirming Treg identity and stability according to published scientific guidelines.[47,48] Tacrolimus-treated FKBP12^{KO}-Treg and untreated Treg^{WT} cluster distributions indentified in CITEseq experiments were similar, confirming FKBP12^{KO} did not alter the Treg product composition.[18] Furthermore, common phenotypic markers were similarly expressed by FKBP12^{KO}-Treg and Treg^{WT}. Tacrolimus is known to stabilize CD25 and induce loss of FOXP3 upon adoptive Treg transfer *in vivo*.[49] Our *in vitro* data shows stable CD25 protein expression in presence of tacrolimus in both FKBP12^{KO}-Treg and Treg^{WT}, as well as stable FOXP3 expression and demethylation. Although FKBP12^{KO}-Tregs exhibited modestly reduced CD25 expression under tacrolimus-free conditions, this did not result in impaired IL-2 signaling, as STAT5 phosphorylation remained intact. Proliferative advantage in tacrolimus-treated FKBP12^{KO}-Treg was shown by enriched gene sets e.g., *Myc*, *SLC2A1* (GLUT1), and *STATA/B*. The reduced suppressive activity observed in classical *in vitro* suppression assays under these conditions is therefore most plausibly explained by altered IL-2 competition rather than a loss of intrinsic regulatory capacity.[50–52] Interpretation of suppression assays under tacrolimus exposure is inherently limited by the dominant antiproliferative effects of the drug on Tresp, and thus FKBP12^{KO}-Teff suppression was shown instead. Importantly, FKBP12^{KO}-Treg showed increased suppressive capacity under tacrolimus and everolimus treatment proving the concept of superiority upon exposure to immunosuppressants binding to FKBP12, which was the underlying hypothesis of this study. The increased suppressive capacity of FKBP12^{KO}-Treg under everolimus treatment lies in the preserved mTORC1 signaling as everolimus is also depending on FKBP12 as adaptor protein. In reliance to that, proteomic analysis of FKBP12^{KO}-Treg showed increased LDHA and Phosphoglycerate Mutase (PGAM)-1, two proteins necessary for IL-2 dependent PI3K/mTORC1 signaling and support of FOXP3 expression as well as Treg homeostasis.[21–24] Prednisolone impact was preserved in FKBP12^{KO-}Treg, due to the loss of FKBP12 binding to the steroid-glucocorticoid receptor complex.[53] Of note, Treg suppress by a myriad of additional mechanisms,[52] but there is currently no standardized assay to evaluate all modes of action. Consistent with preserved regulatory activity, FKBP12^{KO}-Tregs showed increased expression of several molecules associated with immune regulation, including *IL-10*, *CTLA4*, and *CD73*, and *ICOS* particularly in the presence of tacrolimus.[52,54–57] Single-cell transcriptomic and proteomic analyses further demonstrated that tacrolimus-treated FKBP12^{KO}-Tregs closely resembled untreated Treg^{WT} in their overall state composition, indicating that resistance to tacrolimus does not fundamentally alter Treg identity or heterogeneity.[18] IL-2 protein signaling was increased in FKBP12^{KO}-Treg in part compensating for CD25 reduction in presence of tacrolimus, hereby highlighting alternative signaling pathways such as heterodimeric CD25-chemokine receptor pathway.[58] In addition, this is underlined by the increased gene expression of *IL2RA* and *IL2RB* instead of *IL2RG* in FKBP12^{KO}-Treg only under tacrolimus.[59] Low frequencies of IFN-γ-producing cells, absence of T-box expressed in T cells (T-bet) and nuclear factor kappa-light-chain-enhancer of activated B cells (NFKB1/2) induction, as well as stable FOXP3 expression argue against acquisition of inflammatory or destabilized phenotypes. Subtle shifts toward Guanine-Adenine-Thymine-Adenine binding protein (GATA)-3-associated programs remained within

Figure 6. Tacrolimus resistance and CsA safety switch are visualized by CITE-seq of FKBP12^{KO}-Treg, immune check-points, and proliferation markers are regulated under tacrolimus

Where indicated, Treg products were exposed to 6 ng/mL tacrolimus or 120 ng/mL CsA. For all dot plots, the size of each dot represents the percentage of cells within the condition expressing the target gene, while the color intensity (ranging from light gray to dark-colored) indicates the scaled average expression level. CITE-seq was performed at day 21 and day 28, the latter after a 7-days exposure to tacrolimus or CsA or medium as a control from $n = 2$ donors. (A) UMAP clustering of all analyzed cells showing 14 different clusters. The assigned clusters are marked based on the color code indicated. (B) Cluster distribution is shown for an unstimulated state and after 6 h stimulation with PMA/ionomycin on day 21. (C) Cluster distribution is shown after exposure to the indicated immunosuppressant for 7 additional days. (D) A comparison of gene expression data between FKBP12^{KO}-Tregs to Treg^{WT} in unstimulated and stimulated conditions. Log2 fold change (Log2FC) is displayed after 28 days of exposure to ctrl medium, Tac, and CsA. (E) Volcano plot displaying differential gene expression within FKBP12^{KO}-Treg and Treg^{WT} on day 21. (F) Dotplot displaying exhaustion markers in Treg comparing FKBP12^{KO}-Treg and Treg^{WT} in stimulated and unstimulated state on day 28. (G) Volcano plots showing proliferation markers (red) in FKBP12^{KO}-Treg vs. Treg^{WT} under medium and tacrolimus exposure, differential statistics were mapped from the global paired edgeR model. Vertical dashed blue lines denote a 2-fold expression shift, and the horizontal dashed blue line signifies statistical significance (FDR <0.05). (H) Dotplot showing Treg phenotype markers on day 28 for FKBP12^{KO}-Treg vs. Treg^{WT} in stimulated and unstimulated condition. (I) Differential gene expression of *IL-10* is plotted for unstimulated and stimulated FKBP12^{KO}-treg in comparison to Treg^{WT} and under supplementation of ctrl medi-um, Tac and CsA for 7 additional days.

Table 1. Assessment and validation of KO-efficacy in FKBP12^{KO}-Treg

	Validation cell products		
Assessment	Run 1	Run 2	Run 3
Viability (%)	95	94	95
CD4+CD25+FOXP3+ (%)	95	95	97
CD8+ (%)	0.2	0.1	0.2
IL-2+ producers (%)	1	1	0
IFN-γ+ producers (%)	5	0	0
KO-efficiency (%)	79.3	99.9	98.2

Data from GMP compliant validation runs is shown containing parameters such as % of viability, Treg, CD8 contamination, IL2/IFNγ/TNFα producers and final KO-efficacy.

the expected spectrum of Treg states and do not indicate lineage deviation.[60] Although, only very minor proportions of IFN-γ producers were detected in flow cytometry, this protein needs to be further investigated in FKBP12^{KO}-Treg. However IFN-γ secreting Treg are described to act superior compared to non-IFN-γ secreting Treg in e.g., the context of TH1 inflammation and IFN-γ is of functional importance in alloantigen-reactive Treg.[61] Taken together, the presented data support the superior functionality of FKBP12^{KO}-Treg by its preserved function upon co-administration with tacrolimus as well as the robustness and reproducibility of the validated manufacturing process.

Safety

Safety considerations are central for gene-edited cellular therapies. To minimize risk, we employed a vector-free CRISPR-Cas9 ribonucleoprotein approach using a high-fidelity Cas9 enzyme, thereby avoiding persistent nuclease expression and reducing off-target potential. When comparing Treg phenotype characteristics to published Treg marker assessment across clinical trials in transplantation and GVHD as well as to Treg^{WT} expanded in parallel under the same conditions, no discernable phenotype limitations of “TregTacRes” could be shown. Two research grade studies on Treg editing for autoimmunity and immunopathologies were included depicting gene-editing rates in Treg that could be correlated and matched to our own editing rates ranging between 60% and 80%.

For the regulatory Chemistry, Manufacturing, and Controls (CMC) package, we investigated off- and on-target gene editing frequencies, clonal expansion as well as in depth product characteristics including impurities and functional performance in suppression assays. Off-target sites were nominated using a multitude of orthogonal *in silico* and empirical approaches and deep sequenced. We observed a variable background indel rate and compared this to previously published frequencies from 263 unique gRNAs and found it was elevated in comparison to what has been previously reported in control samples. We hypothesize this may be due to homopolymer repeats in the on/off-target sequences of the FKBP12 gRNA, which is a known motif for heightened error rates from Illumina sequencing.[28] While four off-targets were identified with low frequencies of significantly enriched events (0.01%–0.5%), no off-targets were directly in exonic coding regions with known direct impacts on Treg biology e.g., Golgi-localized, -ear-containing, ARF-binding proteins (*GGA3*) (vesicular trafficking), *NUP62CL* (nuclear pore related gene), *ATP11AUN* (pseudogene), or *NHS* (rare developmental disease gene), and indels at these off-target sites only led to variants of unknown significance via ACMG classification standards.[57,58] This assessment is further supported by the finding that, to date, our data show no evidence of clonal dominance, malignant transformation, or loss of repertoire diversity in FKBP12^{KO}-Tregs. Further safety is granted by the process design yielding a pure starting population after the isolation procedure to avoid any possible editing of contaminating Teff. Even in case of FKBP12^{KO} in Teff, we showed that FKBP12^{KO}-Treg can effectively control these in suppression assays.

For safety of clinical products, we will monitor the quality regarding FKBP12^{KO}-editing frequencies by ddPCR, purity and impurities regarding CD4, CD8, FOXP3, CD25, IFN-γ, and IL-2 producers post-PMA/ionomycin stimulation, TSDR demethylation, sterility testing according to Pharmacopoeia, and absence of mycoplasma and endotoxin and TCR sequences. In addition, we will store retention samples for the possibility of further testing in case of adverse events. Moreover, we plan a detailed therapy response monitoring of the gene-edited Treg product after infusion into patients, e.g., with follow-up ddPCRs and TCR sequencing from patient blood as well as biopsies and urine where available, extensive leukocyte profiling by multiparameter flow cytometry and check for inflammatory events such as cytokine storm after infusion into the patient as well as over-immunosuppression in form of reactivations of chronic viruses, HLA-DR expression on monocytes or tumor formation.

Conclusions

In summary, this study demonstrates that FKBP12^{KO}-Tregs retain stable regulatory identity and function while remaining operational under tacrolimus exposure, addressing a fundamental barrier to integrating Treg therapy with standard immunosuppression. The successful establishment of a GMP manufacturing process provides the foundation for future first-in-human evaluation to determine whether co-administration of FKBP12^{KO}-Tregs with tacrolimus can improve immune control and reduce long-term dependence on systemic immunosuppression in transplant recipients.

MATERIALS AND METHODS

All information on antibodies is attached in Table S1 and information on materials in Table S2.

Blood sampling and ethics

We recruited a total of 15 healthy volunteers who gave a written declaration of consent. This study was approved by the Ethics Committee of Charité-Universitaetsmedizin Berlin as EA4/091/19 and EA1/052/22.

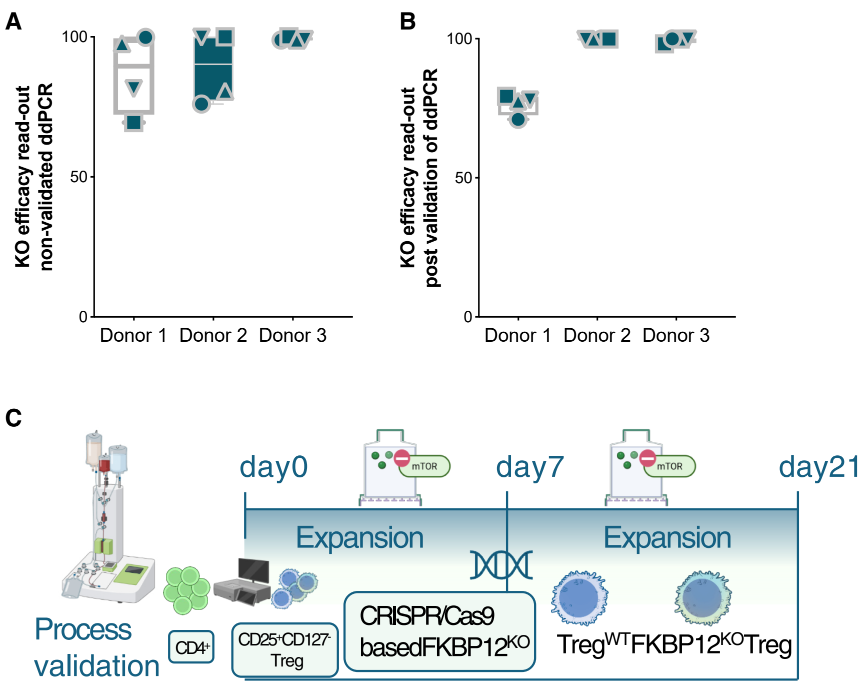

Figure 7. Validation results of KO-efficacy in FKBP12^{KO}-Treg

KO-efficacy results are shown in (A) for non-validated method and in (B) for the validated approach. Three different donors were analyzed for their FKBP12 KO-efficacy while using 4 independent replicate samples each. (C) Schematic description of the validation process for GMP compliance. BioRender used.

Isolation and culture of Treg

CD4$^+$ T cells were isolated from peripheral whole blood either by a research grade or a GMP protocol; i.e., EasySep Human CD4$^+$ T cell Enrichment Kit (STEMCELL Technologies) and CliniMACS Plus device (Miltenyi Biotec), respectively. CD4$^+$ cells subsequently underwent fluorescently activated cell sorting (FACS) using MACSQuant-Tyto (Table S1, Miltenyi Biotec). Tregs were thereafter cultured with X-VIVO-15 medium (Lonza) with 1% penicillin/streptomycin and 10% fetal calf serum (FCS) supplemented with rapamycin at a concentration of 100 nM/mL and recombinant human IL-2 at a concentration of 1,250 IU/mL (Table S2). Tregs were subjected to expansion over the first 7 days with anti-CD3/CD28-MACSiBeads at 4:1 ratio and following 14 days at 1:1 ratio replenished every other day when cultivated in 24 well plates (research grade, pre-GMP). For GMP-grade expansion, Tregs were cultured in G-Rex bioreactors (G-Rex 10 and 100, Wilson Wolf Manufacturing). An aliquot of the negative fraction of the FACSort, i.e., non-Tregs (Tcon) served as control, and was expanded using identical conditions. In addition, expanded and cyro-preserved CMV-antigen-specific FKBP12^{KO}-Teff was included as control samples for the proteomics and CITE-seq analysis.

FKBP12 knockout in Treg

On day 7, the Tregs were electroporated with ribonucleoprotein (RNP) complexes using the Amaxa-P3-primary-cell-4D-Nucleofector-X-Kit-L and the Amaxa-Nucleofector-4D (Lonza); after pre-complexing 10 μg Alt-R S.p. (HiFi) Cas9 Nuclease V3[62] (Integrated DNA Technologies) with 15 μg 2O′-methyl-3′phosphothioate-modified sgRNA (Synthego).[17,18] MOCK controls were treated with Cas9 nuclease only. The same protocol was applied to the control CMV-antigen specific FKBP12^{KO}-Teff.

Phenotypic and functional cytokine profiling

Phenotypic and functional characterization was performed by flow cytometry. After resting overnight in 10% FCS containing RPMI, 1×10^6 cells were stimulated with phorbol-12-myristate-13-acetate (PMA) at 10 ng/mL and ionomycin at 2.5μg/mL at 37°C for 6 h. Dimethyl sulfoxide was added to the unstimulated control. Afterward, cells were labeled with antibodies (Table S1). For intracellular staining the cells were fixed and permeabilized with the FoxP3/Transcription Factor Staining Buffer kit and stained for activation markers (Table S1). Cells were measured using flow cytometers (i.e., LSR II Fortessa/Cytoflex LX) and analyzed using FlowJo (v.10). Following 21 days of expansion, the pSTAT5 levels were assessed, pre-stained cells were seeded at range of concentrations (0.5 × 1060.4 × 10 × 60.5 × 106 cells per well), stimulated at varying IL-2 concentrations (0, 1, 10, 100, 1,000 U/mL) for 30 min at 37°C.

T cell proliferation suppression assay

After resting overnight in 10% FCS-containing RPMI, Treg suppressive capacity was analyzed on freshly isolated, autologous CD3$^+$ T-cells (Human T cell enrichment cocktail, STEMCELL Technologies), namely Tresp that were either unedited Tresp^{WT} or edited FKBP12^{KO}-Tresp respectively. Tresp were stained with 10 μM carboxyfluorescein diacetate succinimidyl ester (CFSE; Sigma-Aldrich) for 3 min at room temperature before stopping the reaction with 10 mL of FCS and 40 mL of RPMI. Following that, autologous Treg^{WT} and FKBP12^{KO}Treg were co-cultured at various Treg/Tresp ratios under stimulation with anti-CD3/CD28 microbeads (Treg Suppression Inspector, Miltenyi).[63] Tresp WT and FKBP12^{KO}-Tresp proliferation was investigated under different immunosuppressants. The following immunosuppressants were implemented: 6 ng/mL tacrolimus, 8 ng/mL everolimus as well as 0.57 μg/mL prednisolone. The percentage of Tresp/T cell proliferation, detected by the level of Carboxyfluorescein succinimidyl ester (CFSE) dilution, was calculated in the presence and absence of Treg. Extracellular flow cytometric staining including viability assessment as well as CD3, CD4, and CD8 was

implemented, measured using the LSR II Fortessa flow cytometer and analyzed using FlowJo.

FKBP12-knockout efficiency analysis and validation using digital droplet PCR

Editing efficiency was determined, as previously described, using the Inference-of-CRISPR-Edits (ICE) algorithm (Synthego Corporation).[17,18,64]

During GMP process development, the following ddPCR assay was established. On day 0.5 × 10^6 cells of the negative fraction of the Tyto-sort were cryo-stored to elaborate on KO-efficacy. In addition, 5 × 10^6 cells of the FKBP12^{KO}-Treg were taken for DNA isolation of both samples using the QIAmp DNA kit (QIAGEN) according to the manufacturer's protocol on day 17. Following that, DNA concentration is measured using the Qubit Assay system (Thermo Fisher Scientific) and samples undergo ddPCR for KO-efficacy detection. KO-efficacy data from biological samples is correlated to a standard curve generated by KO controls with 50%, 75%, and 100% KO rate (deviation of ±10% allowed). The following sequences for ddPCR probes were used: RTP28 (5'-/5HEX/CAC CTT CCC CAA GC + G CGG/3IABkFQ/-3′) and RT-P22 (5'/56-FAM/AGC CGC CGC/ZEN/GCG CCA CTA CT/3IABkFQ/-3′) in combination with the following primers: Primer RT-25 (5′-ATG GGA GTG CAG GTG GAA ACC ATC -3′) and primer RT-26 (5′-CGC TGG GCC CCC GAC TCA -3′).

Methylation profiling

The TSDR methylation status was assessed using the Infinium-Methylation-EPIC-Kit. 200–250 ng of DNA was subjected to bisulfite conversion using EZ-DNA-Methylation-Gold-Kit. Bead Chips were imaged on Illumina's Microarray Scanner iScan. Prior to sequencing by Genewiz/Azenta, amplicons were purified with QIAquick-PCR-Purification-Kit and normalized to 20 ng/μL. Methods applied as per manufacturer's instruction.

The raw intensity data files (IDAT) were processed with the minfi package (v.1.42.0) using quantile normalization. Probes were removed if they (1) failed the detection-*p* value filter ($p < 0.01$), (2) were flagged as cross-reactive, (3) were listed in the updated Illumina manifest (Infinium MethylationEPIC v.1.0, 13-Mar-2020), or (4) overlapped known single nucleotide polimorphisms (SNPs), the latter being excluded with the minfi function dropLociWithSnps. Differentially methylated positions (DMPs) were identified with the limma package (v.3.52.2). Gene-set enrichment was performed on the full set of DMPs with the methylglm function from the methylGSA package (v.1.14.0). The analysis was adjusted for the number of CpGs per gene and employed pathway definitions from the KEGG database. This method accounts for gene length and incorporates the *p* values of all CpGs associated with each gene.

T cell receptor sequencing

DNA was extracted using All-Prep-DNA/RNA-Kit (QIAGEN) and subjected to TCR-β sequencing (Adaptive Biotechnologies) and analyzed with the ImmunoSEQAnalyzer 3.0 software.

Proteomic characterization by mass spectrometry

1 × 10^6 Tregs were pelleted, snap-frozen, and stored at −80°C before subjecting them to mass spectrometry-based proteome analysis as described previously.[65] Peak lists were searched and compared against the human Swiss-Prot database using PEAKS-studio-proteomics v.10.6 (Bioinformatics Solutions). Peptide identification was performed using PEAKS-DB combined with PEAKS-de-novo sequencing. Label-free quantification with PEAKS-Q was used, with a false discovery rate set to 0.01.

DigiWest

T cell lysates were subject to SDS-PAGE. Eluted proteins were incubated with protein specific antibodies as described previously.[25] Antibody specific signals were evaluated using DigiWest analysis tool and subjected to non-parametric statistical analysis and hierarchical clustering.

Cellular indexing of transcriptomes and epitopes sequencing

Treg^{WT}, FKBP12^{KO}-Treg, and Tcon were pooled post-labeling with TotalSeqC anti-human antibodies for surface characterization (Table S3). Single-cell suspensions were loaded onto Next-GEM-Chip-G. Tagged antibody, transcriptome, and TCR libraries were prepared using the Chromium Single Cell 5′ Library & Gel Bead Kit as well as the Single Cell 5′ Feature Barcode Library Kit. Libraries for gene expression and CITE-seq were prepared using Single-Index-Kit-T-Set-A/Single-Index-Kit-N-Set A and quantified using Qubit-HS-DNA-assay kit. Further, fragment sizes were determined using 2100-Bioanalyzer with the High-Sensitivity-DNA-Kit. Sequencing was performed on a NextSeq500 device using High-Output-v.2-Kits. Additional information on CITE-seq computational analysis can be found in the supplemental information section.

Expansion of Treg products in the presence of IS

Following 21 days of expansion, the Tregs were cultured in 24-well plates for 3 or 10 days with or without tacrolimus (stock solution solved in ethanol 96%, reconstituted in PBS) and CsA (reconstituted in Ampuwa) at concentrations of 6 ng/mL and 120 ng/mL, respectively. Furthermore, classical triple immunosuppression was implemented by combining 6 ng/mL tacrolimus with prednisolone at a concentration of 0.57 μg/mL and Mycophenolate-mofetil at a concentration of 2.7 μg/mL.

Off-target nomination using UNCOVERseq

To nominate off-targets of the FKBP12 sgRNA, UNCOVERseq was performed as previously described.[66] Briefly, HEK293-Cas9 cells HEK293-Cas9 (ATCC) cells were cultured in Eagle's Minimum Essential Medium (EMEM; ATCC) supplemented with 10% FBS at 37°C with 5% CO2. For each transfection, 8.0 × 10^5 cells were washed with 1X phosphate-buffered saline, and resuspended in 20 μL of solution SF (Lonza). Following this, 5 μM gRNA and 0.5 μM dsODN were added to the SF solution. This mixture was transferred into 1 well of a 96-well Nucleocuvette plate (Lonza) and electroporated using program DS-150 with biological triplicate treatment/control samples. Following electroporation, cells were

transferred to a 6-well plate preheated with EMEM and were incubated at 37°C with 5% CO2 for 72 h. After incubation, genomic DNA (gDNA) was extracted using the MonarchTM Spin gDNA Extraction Kit (New England Biolabs) according to the manufacturer's instructions, eluted in low-EDTA TE buffer (IDT, 11-05-01-05), and quantified using a NanoDrop 8000 UV-Vis Spectrophotometer (ND-8000-GL). 500 ng of purified gDNA was enzymatically fragmented and adapter-ligated using the xGen DNA Library Prep EZ UNI kit along with the xGen Deceleration Module (IDT, xGen DNA Library Prep EZ UNI 96 rxn, 10009822; xGen Deceleration Module 96 rxn, 10009823) according to the manufacturer's instructions and cleaned with AMPure XP beads (Beckman).

Off-target nomination using CAST-seq

CAST-seq analyses were performed as previously described, with some adjustments to the workflow.[67,68] In brief, the average fragmentation size of the genomic DNA was aimed at a length of 500 bp, and the libraries were sequenced on a NovaSeq 6000 using 2 × 150 bp paired-end sequencing (GENEWIZ, Azenta Life Sciences). Sites under investigation were categorized as off-target mediated translocation (OMT) if the *p*-value met the cut-off of 0.005.

Off-target nomination using in silico models

To nominate sites *in silico,* CALITAS[69] was used to nominate off-target sites both in the reference genome (GRCh38) and with consideration of known mutations from haplotype resolved variants from the Human Genome Diversity Project (HGDP) and the Genome Aggregation Database (gnomAD) occurring at >1% in any of the annotated super-populations. Super populations evaluated included African (AFR), Admixed American (AMR), East Asian (EAS), Non-Finnish Eurpoean (NFE), and South Asian (SAS) populations.

Computational analysis for UNCOVERseq

Following NGS (2 × 150), analysis of UNCOVERseq data was performed as previously described.[66] Briefly, Illumina adapters and UMIs were identified and annotated using Picard MarkIlluminaAdapters. Tag sequences were identified and trimmed using Cutadapt v.4.248. Sequencing reads were aligned to hg38 (GRCh38) reference genome using BWA mem v.0.7.15 and UMI consensus reads were generated based on consensus from a single-strand (minimum UMI consensus size = 1) using fgbio v.0.7.0 (https://github.com/fulcrumgenomics/fgbio). Nomination of candidate off-target sites began by using mapped UMI consensus reads to create a flanked search space (±40 bp) to perform alignment between the guide and empirical target region using a glocal implementation of the Needleman-Wunsch alignment. After a candidate match to the gRNA spacer region was identified in the sequencing data, nominated off-target sites were identified using a hypergeometric test with multiple testing correction (Benjamini-Hochberg; FDR<0.05) by comparing individual treatment samples and pooled control samples for significant differences in representation between the two. We used the following criteria to nominate off-target sites from this analysis for verification: (1) at least one sample nominated a given site with NGS evidence on both sides of the cut site, (2) Levenshtein distance <7 as determined post-alignment, and (3) significant adjusted *p* value when comparing the frequency of the event to the pooled control(s). Nominated on/off-target sites had additional meta-data added based on alignment/genomic context and were placed into described tiers based on this meta-data (Table S5).

Off-target prioritization

Candidate off-target sites nominated by different methods are associated with method-specific unique enrichment scores, supporting information, and annotations. To prioritize off-targets for confirmation a ruleset was used to determine the appropriate off-target sites for interrogation. Off-targets were prioritized if they met one or more of following criteria: (1) they were tier 1–tier 3 UNCOVERseq nominations, (2) they were derived Abnoba-seq, (3) they were reproducibly derived from CAST-seq, (4) they were an *in silico* derived site with a Levenshtein distance <3 from the intended gRNA spacer, (or 5) they were an *in silico*-derived site positioned within an annotated exonic region. All sites were designed for multiplexed amplicon sequencing using the IDT rhAmpSeq Design Tool (https://www.idtdna.com/pages/tools/rhampseq-design-tool).

Library Preparation—Confirmation

Genomic DNA was extracted from control and genome-edited cells as described previously. Libraries for amplicon NGS were prepared using a previously described rhAmpSeq amplification-based method (IDT) using 100 ng of gDNA input. Briefly, the first round of PCR was performed using target-specific primers. A second round of PCR was used to incorporate P5 and P7 Illumina adapters to the ends of the amplicons for universal amplification. Libraries were purified using Agencourt AMPure XP system (Beckman Coulter) and quantified by Qubit 1X dsDNA HS Assay kit (Invitrogen) before sequencing on the Illumina NextSeq platform (150-bp paired end reads). Read demultiplexing was performed on the resulting BCL files using Picard v.2.18.9 (https://github.com/broadinstitute/picard) IlluminaBasecallsToFastq. Computational analysis of off-target confirmation and off-target translocations was implemented according to published literature and additional information on the computational approach can be found in the supplemental information section.[70,71]

Statistics

All data points represent biological replicates. Analysis was performed using GraphPad PRISM (v.8.3) and R (2024.12.0 + 467). Data are shown as mean ± SEM unless otherwise stated.

DATA AND CODE AVAILABILITY

Results will be made accessible on repositories and according to the FAIR data principles upon successful publication as well as they will be shared upon reasonable request.

ACKNOWLEDGMENTS

We thank Anne Schulze for technical assistance in DNA methylation analysis and Geoffroy Andrieux for bioinformatic CAST-seq analyses. Furthermore, we thank Grit Nebrich for sample preparation (BIH-Core Unit Imaging Mass Spectrometry). In addition, we thank Dr. Harald Stachelscheid and Dr. Valeria Vallone Fernandez from the Core Unit for Stem Cells and Organoids (CUSCO) at the Berlin Institute

of Health for access to their devices and technical support. This work was funded by the ReSHAPE project/European Union's Horizon 2020 research and innovation program under grant agreement no. 825392. In addition, data generated regarding effector T cells were funded by the BMFTR under grant agreement no. 01EK2104A. Further funding was granted by the Einstein Center for Regenerative Therapies (PhD grant), Charité – Universitaetsmedizin Berlin and by Berlin Institute of Health (Crossfield grant Treg, Ad Hoc Grant from the Clinical Incubator for UNCOVERseq). T.C. received funding from the European Union under grant agreement no. 101057438 (geneTIGA).

AUTHOR CONTRIBUTIONS

Conceptualization, L.A., M.S.-H, P.R., and H.-D.V.; methodology: P.R., F.I., A.R., D.K.,J.K.P., T.C. and D.L.W., single cell sequencing: M.-F.M., proteomics: O.K., epic arrays: K.O., DigiWest: M.F.T., S.F.; Software and formal analysis, single cell/proteomics and epic array: S.S., A.G., P.D., F.H., O.K., K.O., L.E.; Investigations: G.Z., C.B.-M, S.R.-S., Y.P., D.J.W., M. Stein, H.H., I.S., S.D., J. Kath, L.P., S.P., R.F., L.A., D.L.W., S.M., M. Sturgeon, A.J., R.T., G.L.K., A.S.-P., O.U., C.B., G.R., J.K., O.M., M.M.K., P.D., M.-F.M., N.W., S. Schulenberg; Data Curation, S. Schlickeiser, A.G., P.D., F. Heinrich, F. Hamm, I.J.C.D.d.l.C.; Writing, L.A., G.Z., L.-M.B., J. Kaeda; Review: all co-authors; Visualization, L.A., M.S.-H, L.-M.B, M.M.K., G.Z.; Supervision, L.A., M.S.-H., P.R.; Project Administration, L.A., M.S.-H., P.R.; Funding Acquisition, P.R., M.S.-H., L.A.

DECLARATION OF INTERESTS

The authors declare that the research was conducted as part of a collaboration agreement between Charité Universitaetsmedizin Berlin and Integrated DNA Technologies (IDT). IDT provided certain reagents and performed experiments. R.T., M.Sturgeon, O.U., C.B., G.R., T.L.O., G.L.K., and A.J. are employees of IDT, which offer reagents for sale similar to some of the compounds described in the manuscript. Products and tools supplied by IDT are for research use only. Purchaser and/or user are solely responsible for all decisions regarding the use of these products and any associated regulatory or legal obligations. P.R., H.-D.V., D.L.W., M.S.-H. and L.A. hold a patent for immunosuppressant-resistant T-cells for adoptive immunotherapy (PCT/EP2021/072651), T.C. is an inventor of CAST-Seq (patent US11319580B2). P.R., H.-D.V. and D.L.W. founded the startup company TCbalance, which licensed the Treg part of PCT/EP2021/072651 from Charité-Universitaetsmedizin Berlin.

SUPPLEMENTAL INFORMATION

Supplemental information can be found online at https://doi.org/10.1016/j.omta.2026.201735.

REFERENCES

1. He, X., Smeets, R.L., Koenen, H.J.P.M., Vink, P.M., Wagenaars, J., Boots, A.M.H., and Joosten, I. (2011). Mycophenolic acid-mediated suppression of human CD4+ T cells: more than mere guanine nucleotide deprivation. Am. J. Transplant. *11*, 439–449.
2. Allison, A.C., and Eugui, E.M. (2000). Mycophenolate mofetil and its mechanisms of action. Immunopharmacology *47*, 85–118.
3. Tsuda, K., Yamanaka, K., Kitagawa, H., Akeda, T., Naka, M., Niwa, K., Nakanishi, T., Kakeda, M., Gabazza, E.C., and Mizutani, H. (2012). Calcineurin inhibitors suppress cytokine production from memory T cells and differentiation of naive T cells into cytokine-producing mature T cells. PLoS One *7*, e31465.
4. Brinkmann, V., and Kristofic, C. (1995). Regulation by corticosteroids of Th1 and Th2 cytokine production in human CD4+ effector T cells generated from CD45RO- and CD45RO+ subsets. J. Immunol. *155*, 3322–3328.
5. Herold, M.J., McPherson, K.G., and Reichardt, H.M. (2006). Glucocorticoids in T cell apoptosis and function. Cell. Mol. Life Sci. *63*, 60–72.
6. Takahata, Y., Nomura, A., Takada, H., Ohga, S., Furuno, K., Hikino, S., Nakayama, H., Sakaguchi, S., and Hara, T. (2004). $CD25^{+}CD4^{+}$ T cells in human cord blood: an immunoregulatory subset with naive phenotype and specific expression of forkhead box p3 (Foxp3) gene. Exp. Hematol. *32*, 622–629.
7. Yu, N., Li, X., Song, W., Li, D., Yu, D., Zeng, X., Li, M., Leng, X., and Li, X. (2012). $CD4^{+}CD25^{+}CD127$ (low/-) T cells: a more specific Treg population in human peripheral blood. Inflammation *35*, 1773–1780.
8. Toker, A., Engelbert, D., Garg, G., Polansky, J.K., Floess, S., Miyao, T., Baron, U., Düber, S., Geffers, R., Giehr, P., et al. (2013). Active demethylation of the Foxp3 locus leads to the generation of stable regulatory T cells within the thymus. J. Immunol. *190*, 3180–3188.
9. Kmieciak, M., Gowda, M., Graham, L., Godder, K., Bear, H.D., Marincola, F.M., and Manjili, M.H. (2009). Human T cells express CD25 and Foxp3 upon activation and exhibit effector/memory phenotypes without any regulatory/suppressor function. J. Transl. Med. *7*, 89.
10. Schreiber, L., Pietzsch, B., Floess, S., Farah, C., Jänsch, L., Schmitz, I., and Huehn, J. (2014). The Treg-specific demethylated region stabilizes Foxp3 expression independently of NF-kappaB signaling. PLoS One *9*, e88318.
11. Baron, U., Floess, S., Wieczorek, G., Baumann, K., Grützkau, A., Dong, J., Thiel, A., Boeld, T.J., Hoffmann, P., Edinger, M., et al. (2007). DNA demethylation in the human FOXP3 locus discriminates regulatory T cells from activated FOXP3(+) conventional T cells. Eur. J. Immunol. *37*, 2378–2389.
12. Polansky, J.K., Kretschmer, K., Freyer, J., Floess, S., Garbe, A., Baron, U., Olek, S., Hamann, A., von Boehmer, H., and Huehn, J. (2008). DNA methylation controls Foxp3 gene expression. Eur. J. Immunol. *38*, 1654–1663.
13. Sawitzki, B., Harden, P.N., Reinke, P., Moreau, A., Hutchinson, J.A., Game, D.S., Tang, Q., Guinan, E.C., Battaglia, M., Burlingham, W.J., et al. (2020). Regulatory cell therapy in kidney transplantation (The ONE Study): a harmonised design and analysis of seven non-randomised, single-arm, phase 1/2A trials. Lancet *395*, 1627–1639.
14. Roemhild, A., Otto, N.M., Moll, G., Abou-El-Enein, M., Kaiser, D., Bold, G., Schachtner, T., Choi, M., Oellinger, R., Landwehr-Kenzel, S., et al. (2020). Regulatory T cells for minimising immune suppression in kidney transplantation: phase I/IIa clinical trial. Br. Med. J. *371*, m3734.
15. Siepert, A., Ahrlich, S., Vogt, K., Appelt, C., Stanko, K., Kühl, A., van den Brandt, J., Reichardt, H.M., Nizze, H., Lehmann, M., et al. (2012). Permanent CNI treatment for prevention of renal allograft rejection in sensitized hosts can be replaced by regulatory T cells. Am. J. Transplant. *12*, 2384–2394.
16. San Segundo, D., Ruiz, J.C., Fernández-Fresnedo, G., Izquierdo, M., Gómez-Alamillo, C., Cacho, E., Benito, M.J., Rodrigo, E., Palomar, R., López-Hoyos, M., and Arias, M. (2006). Calcineurin inhibitors affect circulating regulatory T cells in stable renal transplant recipients. Transplant. Proc. *38*, 2391–2393.
17. Amini, L., Wagner, D.L., Rossler, U., Zarrinrad, G., Wagner, L.F., Vollmer, T., Wendering, D.J., Kornak, U., Volk, H.D., Reinke, P., et al. (2020). CRISPR-Cas9-Edited Tacrolimus-Resistant Antiviral T Cells for Advanced Adoptive Immunotherapy in Transplant Recipients. Mol. Ther. *29*, 32–46.
18. Peter, L., Wendering, D.J., Schlickeiser, S., Hoffmann, H., Noster, R., Wagner, D.L., Zarrinrad, G., Münch, S., Picht, S., Schulenberg, S., et al. (2022). Tacrolimus-resistant SARS-CoV-2-specific T cell products to prevent and treat severe COVID-19 in immunosuppressed patients. Mol. Ther. Methods Clin. Dev. *25*, 52–73.
19. Allan, S.E., Crome, S.Q., Crellin, N.K., Passerini, L., Steiner, T.S., Bacchetta, R., Roncarolo, M.G., and Levings, M.K. (2007). Activation-induced FOXP3 in human T effector cells does not suppress proliferation or cytokine production. Int. Immunol. *19*, 345–354.
20. Pfoertner, S., Jeron, A., Probst-Kepper, M., Guzman, C.A., Hansen, W., Westendorf, A.M., Toepfer, T., Schrader, A.J., Franzke, A., Buer, J., and Geffers, R. (2006). Signatures of human regulatory T cells: an encounter with old friends and new players. Genome Biol. *7*, R54.
21. Zhang, Y.T., Xing, M.L., Fang, H.H., Li, W.D., Wu, L., and Chen, Z.P. (2023). Effects of lactate on metabolism and differentiation of CD4(+)T cells. Mol. Immunol. *154*, 96–107.
22. Fan, M.Y., and Turka, L.A. (2018). Immunometabolism and PI(3)K Signaling As a Link between IL-2, Foxp3 Expression, and Suppressor Function in Regulatory T Cells. Front. Immunol. *9*, 69.
23. Bulygin, A.S., Khantakova, J.N., Shkaruba, N.S., Shiku, H., and Sennikov, S.S. (2022). The role of metabolism on regulatory T cell development and its impact in tumor and transplantation immunity. Front. Immunol. *13*, 1016670.
24. Toriyama, K., Kuwahara, M., Kondoh, H., Mikawa, T., Takemori, N., Konishi, A., Yorozuya, T., Yamada, T., Soga, T., Shiraishi, A., and Yamashita, M. (2020).

T cell-specific deletion of Pgam1 reveals a critical role for glycolysis in T cell responses. Commun. Biol. *3*, 394.

25. Treindl, F., Ruprecht, B., Beiter, Y., Schultz, S., Döttinger, A., Staebler, A., Joos, T.O., Kling, S., Poetz, O., Fehm, T., et al. (2016). A bead-based western for high-throughput cellular signal transduction analyses. Nat. Commun. *7*, 12852.

26. Pedroza-Gonzalez, A., Zhou, G., Singh, S.P., Boor, P.P., Pan, Q., Grunhagen, D., de Jonge, J., Tran, T.K., Verhoef, C., IJzermans, J.N., et al. (2015). GITR engagement in combination with CTLA-4 blockade completely abrogates immunosuppression mediated by human liver tumor-derived regulatory T cells ex vivo. OncoImmunology *4*, e1051297.

27. Camisaschi, C., Casati, C., Rini, F., Perego, M., De Filippo, A., Triebel, F., Parmiani, G., Belli, F., Rivoltini, L., and Castelli, C. (2010). LAG-3 expression defines a subset of CD4(+)CD25(high)Foxp3(+) regulatory T cells that are expanded at tumor sites. J. Immunol. *184*, 6545–6551.

28. Chen, H., Wang, B., Cai, L., Zhang, Y., Shu, Y., Liu, W., Leng, X., Zhai, J., Niu, B., Zhou, Q., and Cao, S. (2024). The performance of homopolymer detection using dichromatic and tetrachromatic fluorogenic next-generation sequencing platforms. BMC Genom. *25*, 542.

29. Veitch, M., Beaumont, K., Pouwer, R., Chew, H.Y., Frazer, I.H., Soyer, H.P., Campbell, S., Dymock, B.W., Harvey, A., Cock, T.A., and Wells, J.W. (2023). Local blockade of tacrolimus promotes T-cell-mediated tumor regression in systemically immunosuppressed hosts. J. Immunother. Cancer *11*, e006783.

30. McCallion, O., Bilici, M., Hester, J., and Issa, F. (2023). Regulatory T-cell therapy approaches. Clin. Exp. Immunol. *211*, 96–107.

31. Dong, S., Hiam-Galvez, K.J., Mowery, C.T., Herold, K.C., Gitelman, S.E., Esensten, J.H., Liu, W., Lares, A.P., Leinbach, A.S., Lee, M., et al. (2021). The effect of low-dose IL-2 and Treg adoptive cell therapy in patients with type 1 diabetes. JCI Insight *6*, e147474.

32. Landwehr-Kenzel, S., Mueller-Jensen, L., Kuehl, J.S., Abou-El-Enein, M., Hoffmann, H., Muench, S., Kaiser, D., Roemhild, A., von Bernuth, H., Voeller, M., et al. (2022). Adoptive transfer of ex vivo expanded regulatory T-cells improves immune cell engraftment and therapy-refractory chronic GvHD. Mol. Ther. *30*, 2298–2314.

33. Mathew, J.M., H.-Voss, J., LeFever, A., Konieczna, I., Stratton, C., He, J., Huang, X., Gallon, L., Skaro, A., Ansari, M.J., et al. (2018). A Phase I Clinical Trial with Ex Vivo Expanded Recipient Regulatory T cells in Living Donor Kidney Transplants. Sci. Rep. *8*, 7428.

34. Chandran, S., Tang, Q., Sarwal, M., Laszik, Z.G., Putnam, A.L., Lee, K., Leung, J., Nguyen, V., Sigdel, T., Tavares, E.C., et al. (2017). Polyclonal Regulatory T Cell Therapy for Control of Inflammation in Kidney Transplants. Am. J. Transplant. *17*, 2945–2954.

35. Tang, Q., Leung, J., Peng, Y., Sanchez-Fueyo, A., Lozano, J.J., Lam, A., Lee, K., Fitch, M., Greenland, J.R., Hellerstein, M., et al. (2022). Selective decrease of donor-reactive T(regs) after liver transplantation limits T(reg) therapy for promoting allograft tolerance in humans. Sci. Transl. Med. *14*, eabo2628.

36. Amini, L., Kaeda, J., Fritsche, E., Roemhild, A., Kaiser, D., and Reinke, P. (2022). Clinical adoptive regulatory T Cell therapy: State of the art, challenges, and prospective. Front. Cell Dev. Biol. *10*, 1081644.

37. Barbarino, J.M., Staatz, C.E., Venkataramanan, R., Klein, T.E., and Altman, R.B. (2013). PharmGKB summary: cyclosporine and tacrolimus pathways. Pharmacogenet. Genom. *23*, 563–585.

38. Dubois, S., Shou, W., Haneline, L.S., Fleischer, S., Waldmann, T.A., and Müller, J.R. (2003). Distinct pathways involving the FK506-binding proteins 12 and 12.6 underlie IL-2-versus IL-15-mediated proliferation of T cells. Proc. Natl. Acad. Sci. USA *100*, 14169–14174.

39. Rivera, A., and Heitman, J. (2023). Natural product ligands of FKBP12: Immunosuppressive antifungal agents FK506, rapamycin, and beyond. PLoS Pathog. *19*, e1011056.

40. Dumont, F.J., Staruch, M.J., Koprak, S.L., Melino, M.R., and Sigal, N.H. (1990). Distinct mechanisms of suppression of murine T cell activation by the related macrolides FK-506 and rapamycin. J. Immunol. *144*, 251–258.

41. McMahon, L.P., Choi, K.M., Lin, T.A., Abraham, R.T., and Lawrence, J.C., Jr. (2002). The rapamycin-binding domain governs substrate selectivity by the mammalian target of rapamycin. Mol. Cell Biol. *22*, 7428–7438.

42. Fraser, H., Safinia, N., Grageda, N., Thirkell, S., Lowe, K., Fry, L.J., Scottá, C., Hope, A., Fisher, C., Hilton, R., et al. (2018). A Rapamycin-Based GMP-Compatible Process for the Isolation and Expansion of Regulatory T Cells for Clinical Trials. Mol. Ther., Methods Clin. Dev. *8*, 198–209.

43. Zeiser, R., Leveson-Gower, D.B., Zambricki, E.A., Kambham, N., Beilhack, A., Loh, J., Hou, J.Z., and Negrin, R.S. (2008). Differential impact of mammalian target of rapamycin inhibition on CD4+CD25+Foxp3+ regulatory T cells compared with conventional CD4+ T cells. Blood *111*, 453–462.

44. Strauss, L., Czystowska, M., Szajnik, M., Mandapathil, M., and Whiteside, T.L. (2009). Differential responses of human regulatory T cells (Treg) and effector T cells to rapamycin. PLoS One *4*, e5994.

45. Zhuo, C., Li, Z., Xu, Y., Wang, Y., Li, Q., Peng, J., Zheng, H., Wu, P., Li, B., and Cai, S. (2014). Higher FOXP3-TSDR demethylation rates in adjacent normal tissues in patients with colon cancer were associated with worse survival. Mol. Cancer *13*, 153.

46. Wang, J., Ioan-Facsinay, A., van der Voort, E. ., Huizinga, T. ., and Toes, R. . (2007). Transient expression of FOXP3 in human activated nonregulatory CD4+ T cells. Eur. J. Immunol. *37*, 129–138.

47. Helmin, K.A., Morales-Nebreda, L., Torres Acosta, M.A., Anekalla, K.R., Chen, S.Y., Abdala-Valencia, H., Politanska, Y., Cheresh, P., Akbarpour, M., Steinert, E.M., et al. (2020). Maintenance DNA methylation is essential for regulatory T cell development and stability of suppressive function. J. Clin. Investig. *130*, 6571–6587.

48. Ou, K., Hamo, D., Schulze, A., Roemhild, A., Kaiser, D., Gasparoni, G., Salhab, A., Zarrinrad, G., Amini, L., Schlickeiser, S., et al. (2021). Strong Expansion of Human Regulatory T Cells for Adoptive Cell Therapy Results in Epigenetic Changes Which May Impact Their Survival and Function. Front. Cell Dev. Biol. *9*, 751590.

49. Singh, K., Stempora, L., Harvey, R.D., Kirk, A.D., Larsen, C.P., Blazar, B.R., and Kean, L.S. (2014). Superiority of rapamycin over tacrolimus in preserving nonhuman primate Treg half-life and phenotype after adoptive transfer. Am. J. Transplant. *14*, 2691–2703.

50. Wendering, D.J., Amini, L., Schlickeiser, S., Reinke, P., Volk, H.-D., and Schmueck-Henneresse, M. (2019). The Value of a Rapid Test of Human Regulatory T Cell Function Needs to be Revised. Front. Immunol. *10*, 150.

51. Miyara, M., and Sakaguchi, S. (2007). Natural regulatory T cells: mechanisms of suppression. Trends Mol. Med. *13*, 108–116.

52. Wagner, D.L., Peter, L., and Schmueck-Henneresse, M. (2021). Cas9-directed immune tolerance in humans-a model to evaluate regulatory T cells in gene therapy? Gene Ther. *28*, 549–559.

53. Janyst, M., Kaleta, B., Janyst, K., Zagożdżon, R., Kozlowska, E., and Lasek, W. (2020). Comparative Study of Immunomodulatory Agents to Induce Human T Regulatory (Treg) Cells: Preferential Treg-Stimulatory Effect of Prednisolone and Rapamycin. Arch. Immunol. Ther. Exp. *68*, 20.

54. Luo, X., Du, G., Chen, B., Yan, G., Zhu, L., Cui, P., Dai, H., Qi, Z., and Lan, T. (2022). Novel immunosuppressive effect of FK506 by upregulation of PD-L1 via FKBP51 in heart transplantation. Scand. J. Immunol. *96*, e13203.

55. Gu, J., Ni, X., Pan, X., Lu, H., Lu, Y., Zhao, J., Guo Zheng, S., Hippen, K.L., Wang, X., and Lu, L. (2017). Human CD39(hi) regulatory T cells present stronger stability and function under inflammatory conditions. Cell. Mol. Immunol. *14*, 521–528.

56. Qureshi, O.S., Zheng, Y., Nakamura, K., Attridge, K., Manzotti, C., Schmidt, E.M., Baker, J., Jeffery, L.E., Kaur, S., Briggs, Z., et al. (2011). Trans-endocytosis of CD80 and CD86: a molecular basis for the cell-extrinsic function of CTLA-4. Science *332*, 600–603.

57. Egen, J.G., and Allison, J.P. (2002). Cytotoxic T lymphocyte antigen-4 accumulation in the immunological synapse is regulated by TCR signal strength. Immunity *16*, 23–35.

58. Sun, H., Lee, H.S., Kim, S.H.J., Fernandes de Lima, M., Gingras, A.R., Du, Q., McLaughlin, W., Ablack, J., Lopez-Ramirez, M.A., Lagarrigue, F., et al. (2023).

IL-2 can signal via chemokine receptors to promote regulatory T cells' suppressive function. Cell Rep. *42*, 112996.

59. Harris, F., Berdugo, Y.A., and Tree, T. (2023). IL-2-based approaches to Treg enhancement. Clin. Exp. Immunol. *211*, 149–163.

60. Wang, L., Liang, Y., Zhao, C., Ma, P., Zeng, S., Ju, D., Zhao, M., Yu, M., and Shi, Y. (2025). Regulatory T cells in homeostasis and disease: molecular mechanisms and therapeutic potential. Signal Transduct. Targeted Ther. *10*, 345.

61. Sawitzki, B., Kingsley, C.I., Oliveira, V., Karim, M., Herber, M., and Wood, K.J. (2005). IFN-gamma production by alloantigen-reactive regulatory T cells is important for their regulatory function in vivo. J. Exp. Med. *201*, 1925–1935.

62. Vakulskas, C.A., Dever, D.P., Rettig, G.R., Turk, R., Jacobi, A.M., Collingwood, M.A., Bode, N.M., McNeill, M.S., Yan, S., Camarena, J., et al. (2018). A high-fidelity Cas9 mutant delivered as a ribonucleoprotein complex enables efficient gene editing in human hematopoietic stem and progenitor cells. Nat. Med. *24*, 1216–1224.

63. Wendering, D.J., Amini, L., Schlickeiser, S., Farrera-Sal, M., Schulenberg, S., Peter, L., Mai, M., Vollmer, T., Du, W., Stein, M., et al. (2024). Effector memory-type regulatory T cells display phenotypic and functional instability. Sci. Adv. *10*, eadn3470.

64. Conant, D., Hsiau, T., Rossi, N., Oki, J., Maures, T., Waite, K., Yang, J., Joshi, S., Kelso, R., Holden, K., et al. (2022). Inference of CRISPR Edits from Sanger Trace Data. CRISPR J. *5*, 123–130.

65. Klein, O., Rohwer, N., de Molina, K.F., Mergler, S., Wessendorf, P., Herrmann, M., Klose, J., and Cramer, T. (2013). Application of two-dimensional gel-based mass spectrometry to functionally dissect resistance to targeted cancer therapy. Proteonomics Clin. Appl. *7*, 813–824. https://doi.org/10.1002/prca.201300056.

66. Kinney, K.J., Jia, K., Zhang, H., Schmaljohn, E., Osborne, T., Thommandru, B., Murugan, K., Sánchez-Peña, A., West, S., Chen, S., et al. (2025). UNCOVERseq Enables Sensitive and Controlled Gene Editing Off-Target Nomination Across CRISPR-Cas Modalities and Systems. Preprint at bioRxiv. https://doi.org/10.1101/2025.05.09.653165.

67. Turchiano, G., Andrieux, G., Klermund, J., Blattner, G., Pennucci, V., El Gaz, M., Monaco, G., Poddar, S., Mussolino, C., Cornu, T.I., et al. (2021). Quantitative evaluation of chromosomal rearrangements in gene-edited human stem cells by CAST-Seq. Cell Stem Cell *28*, 1136–1147.e5.

68. Rhiel, M., Geiger, K., Andrieux, G., Rositzka, J., Boerries, M., Cathomen, T., and Cornu, T.I. (2023). T-CAST: An optimized CAST-Seq pipeline for TALEN confirms superior safety and efficacy of obligate-heterodimeric scaffolds. Front. Genome Ed. *5*, 1130736.

69. Fennell, T., Zhang, D., Isik, M., Wang, T., Gotta, G., Wilson, C.J., and Marco, E. (2021). CALITAS: A CRISPR-Cas-aware ALigner for In silico off-TArget Search. CRISPR J. *4*, 264–274.

70. Kurgan, G., Turk, R., Li, H., Roberts, N., Rettig, G.R., Jacobi, A.M., Tso, L., Sturgeon, M., Mertens, M., Noten, R., et al. (2021). CRISPAltRations: a validated cloud-based approach for interrogation of double-strand break repair mediated by CRISPR genome editing. Mol. Ther., Methods Clin. Dev. *21*, 478–491.

71. Love, M.I., Huber, W., and Anders, S. (2014). Moderated estimation of fold change and dispersion for RNA-seq data with DESeq2. Genome Biol. *15*, 550.

OMTA, Volume *34*

Supplemental information

Clinical development of tacrolimus-resistant regulatory T cells to enable simultaneous immunosuppression and immune regulation

Ghazaleh Zarrinrad, Lisa-Marie Burkhardt, Claudia Beltran-Mestres, Silvina Romero-Suárez, Dimitrios Laurin Wagner, Stephan Schlickeiser, Maik Stein, Désirée Jacqueline Wendering, Iván Juky Carrera Diaz de la Cebosa, Lukas Ehlen, Frederik Hamm, Gavin L. Kurgan, Pawel Durek, Frederik Heinrich, Anamika Giri, Yaolin Pu, Kristy Ou, Henrike Hoffmann, Sandra Muench, Insa Stuewe, Sven Dolling, Oliver McCallion, Jaspal Kaeda, Jonas Kath, Sarah Schulenberg, Niklas Wiese, Abdolreza Nazari, Lena Peter, Samira Picht, Andrea Sánchez-Peña, Olalekan Usman, Christian Brommel, Rolf Turk, Garrett Rettig, Morgan Sturgeon, Thomas L. Osborne, Ashley Jacobi, Rebecca Friedrich, Masako Monika Kaufmann, Julia Klermund, Simon Fink, Markus F. Templin, Andy Roemhild, Daniel Kaiser, Oliver Klein, Toni Cathomen, Mir-Farzin Mashreghi, Fadi Issa, Julia K. Polánsky, Hans-Dieter Volk, Michael Schmueck-Henneresse, Petra Reinke, and Leila Amini

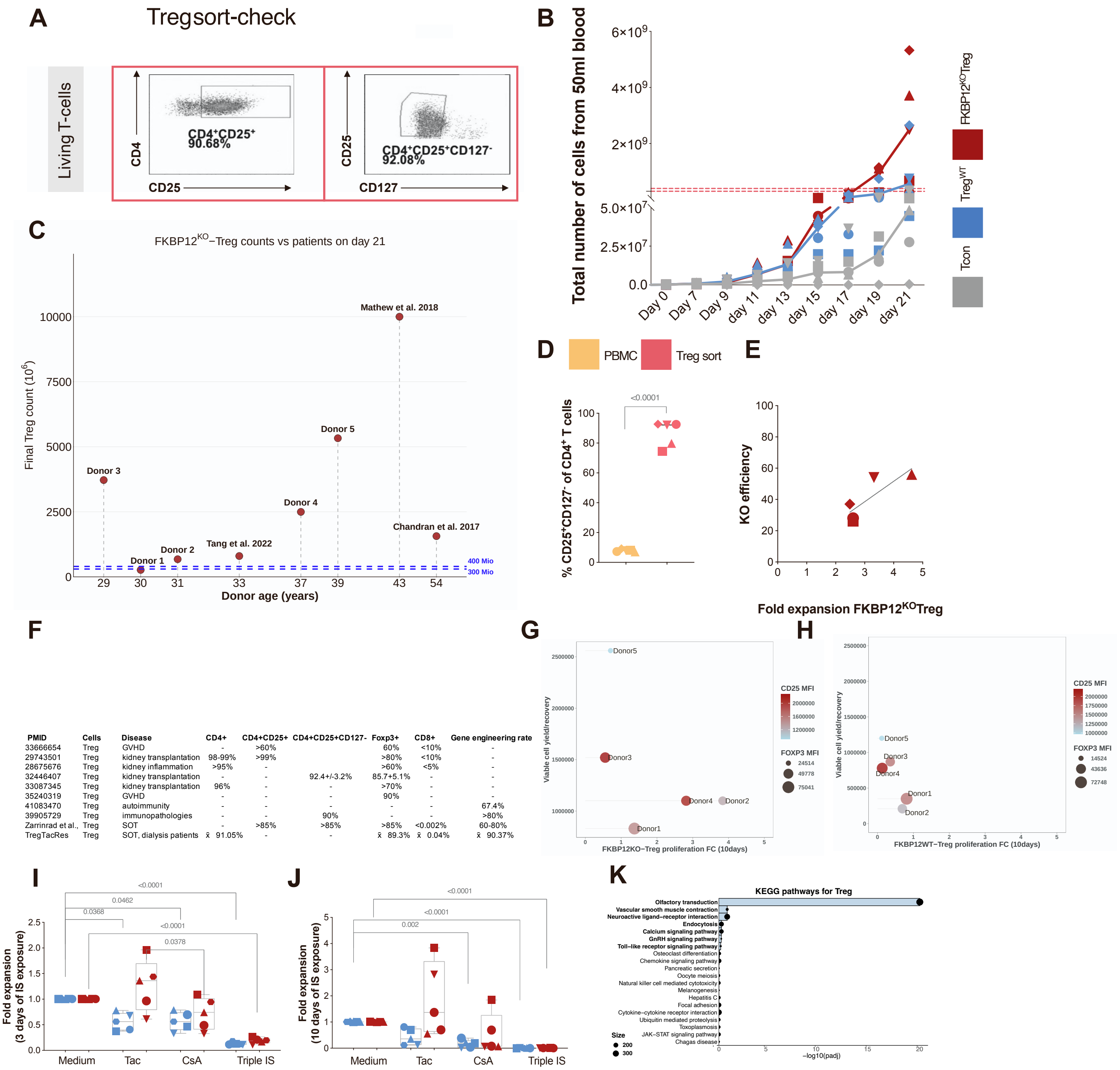

PMID	Cells	Disease	CD4+	CD4+CD25+	CD4+CD25+CD127-	Foxp3+	CD8+	Gene engineering rate
33666654	Treg	GVHD	-	>60%		60%	<10%	-
29743501	Treg	kidney transplantation	98-99%	>99%		>80%	<10%	-
28675676	Treg	kidney inflammation	>95%	-		>60%	<5%	-
32446407	Treg	kidney transplantation	-	-	92.4+/-3.2%	85.7+5.1%	-	-
33087345	Treg	kidney transplantation	96%	-	-	>70%	-	-
35240319	Treg	GVHD	-	-	-	90%	-	-
41083470	Treg	autoimmunity	-	-	-	-	-	67.4%
39905729	Treg	immunopathologies	-	-	90%	-	-	>80%
Zarrinrad et al.,	Treg	SOT	-	>85%	>85%	>85%	<0.002%	60-80%
TregTacRes	Treg	SOT, dialysis patients	x̄ 91.05%	-	-	x̄ 89.3%	x̄ 0.04%	x̄ 90.37%

Figure S1: Tyto sort purity and Treg expansion

(A) Gating strategy for Tyto sort showing CD4, CD25 and CD127 gating. Remaining figures show data from individual donors by different symbols: (B) Visualization of the total number of expanding cells from day 0 to day 21. Tcon (grey), Treg^{WT} (blue), FKBP12^{KO}-Treg (teal), red line marks cut-off for Treg amount in clinical application. (C) Final number of expanded Treg on day 21 (10^6) per donor correlated to donor age (years) and plotted against the same parameters of 3 publications (Tang, Mathew and Chandran). (D) Sort purity shown as the percentage of CD4$^+$CD25$^+$CD127$^-$ T-cells pre- (yellow) and post sorting (light red). (E) The correlation between knock-out efficiency and fold expansion of FKBP12^{KO}-Treg (red). (F) Literature parameters on GMP compliant (top 6) and research grade Treg products (n=2) disease application, phenotype thresholds and gene-editing rates in comparison to Zarrinrad et al., and related TregTacRes trial (bottom 2). (G-H) Correlation of viable cell recovery and proliferation Fold Change (FC) with dot size representing FOXP3 MFI and color-code representing CD25 MFI. (I-J) Fold expansion of FKBP12KO-Treg and TregWT under 3 and 10 days of immunsuppressiva (IS) exposure respectively: data is shown for medium, tacrolimus (Tac), cyclosporine A (CsA) and triple immunosuppression (IS) combining tacrolimus, prednisolone and mycophenolic acid. Each donor is shown by a different symbol and paired t-tests and RM One-way Anova were used, n=5. (K) KEGG pathway analysis for differentially methylated positions between FKBP12^{KO}-Treg and Treg^{WT} and their biological context.

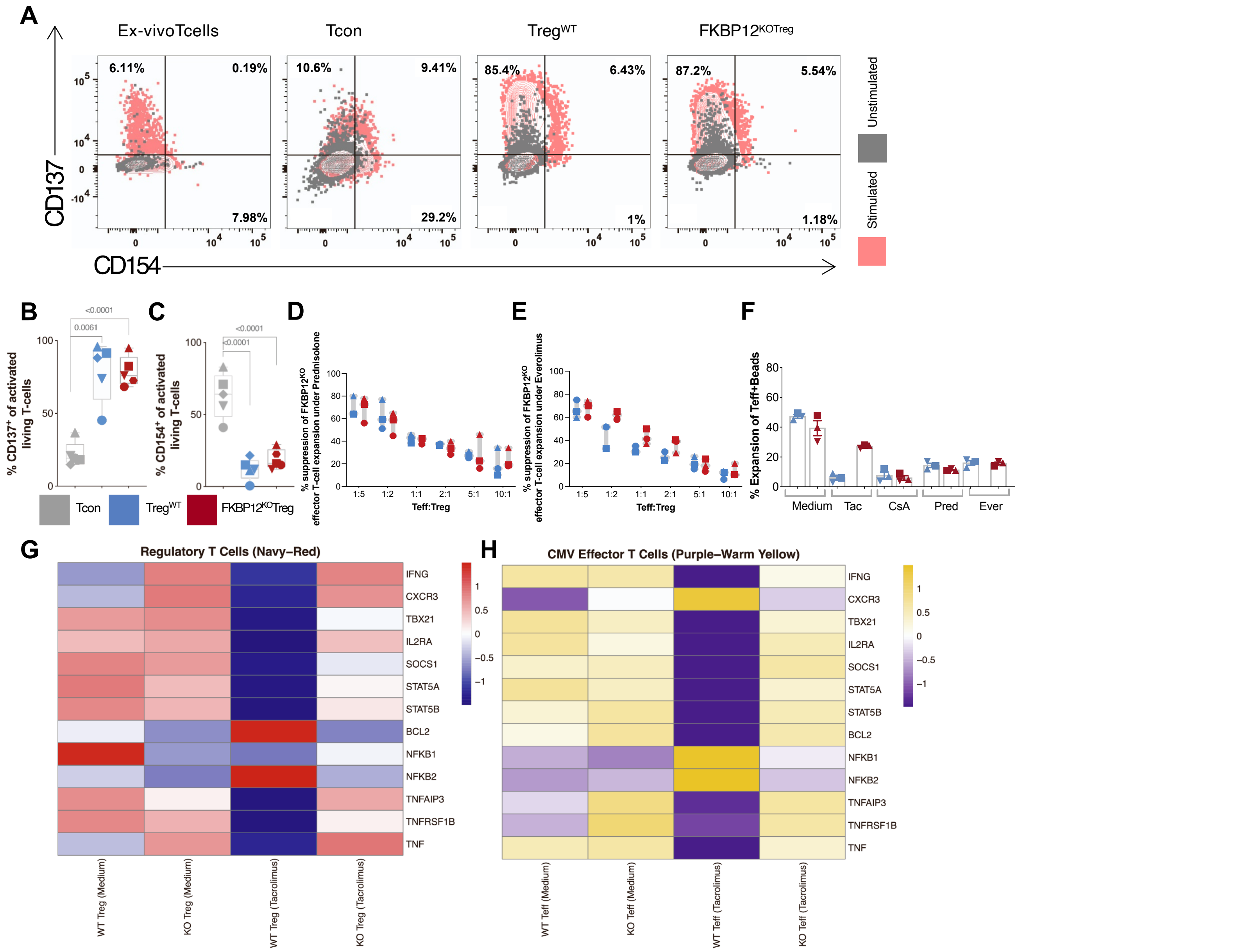

Figure S2: FKBP12^{KO}-Treg activation is comparable with Treg^{WT}

(A) Representative dot plots for CD137 and CD154 expression in ex-vivo T-cells, Tcon, Treg^{WT} and FKBP12^{KO}-Treg on day 21. Unstimulated controls (grey) and stimulated conditions (pink). Remaining figures show individual donors by different symbols. Tcon (grey), Treg^{WT} (blue), FKBP12^{KO}-Treg (red). (B) Percentage of CD137^{+} among the living CD4^{+} T-cells on day 21 in Tcon, Treg^{WT} and FKBP12^{KO}-Treg. (C) Percentage of CD154^{+} among the living T-cells on day 21 in Tcon, Treg^{WT} and FKBP12^{KO}-Treg. For (B) and (C) paired t-test and RM one-way Anova were used, n=5. (D-E) Frequency of suppression of FKBP12^{KO}-Teff in medium control, FKBP12^{KO}-Treg (red), Treg^{WT} (blue) is shown, with n=3 and for different Teff:Treg ratios under (D) prednisolone and (E) everolimus. (F) Proliferation potential of FKBP12^{KO}-Teff (red) and Teff^{WT} (blue) under different immunosuppressants without the influence of Treg. (G-H) Transcriptional profiling of key cytokine signaling cascades across engineered T cell populations. Teff are expanded, cryo-preserved CMV specific T-cells stimulated with CMV peptides IE1 and pp65 for 6h in comparison to Treg that were stimulated with PMA/Iono for 6h. Comparisons can only be made within one heat-map since scaling and experimental conditions differ. Combined heatmap depicting the averaged, scaled single-cell expression of highly curated gene panels associated with Interferon-gamma (IFN-γ), Interleukin-2 (IL-2), and Tumor Necrosis Factor-alpha (TNF-α) signaling pathways. Averaged expression is shown across Wild-Type (WT) and FKBP12-^{KO} Regulatory T Cells (Tregs) and corresponding CMV-Specific Effector T Cells (Teffs) in medium and tacrolimus conditions. Statistical & Visual Analysis: Raw single-cell RNA counts were aggregated and averaged per condition. To highlight relative shifts in pathway activation between treatments without signal washout between the distinct cell lineages, expression values were independently z-scored (scaled) across rows within each cell population prior to merging. The color gradient ranges from navy (downregulated/below mean) to white (mean expression) to firebrick red (upregulated/above mean). Genes exhibiting zero variance across conditions were algorithmically excluded to ensure matrix stability.

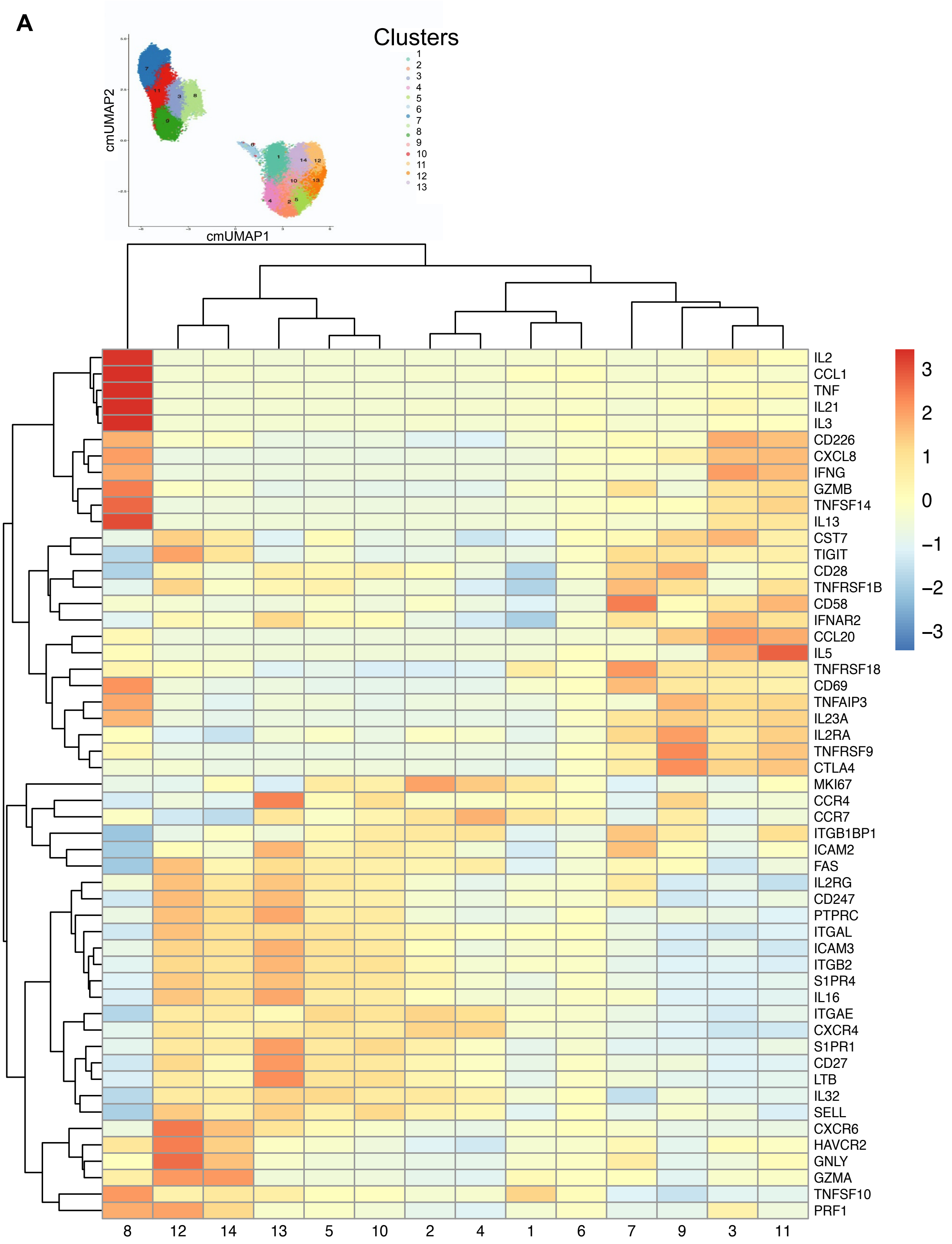

Figure S3: Cellular Indexing of Transcriptomes and Epitope sequencing

Detailed heatmap of RNA expression of T cell-associated genes within different clusters. Cluster distribution is shown for all 14 clusters and differential gene expression within each cluster (1-14) is highlighted within the heatmap.

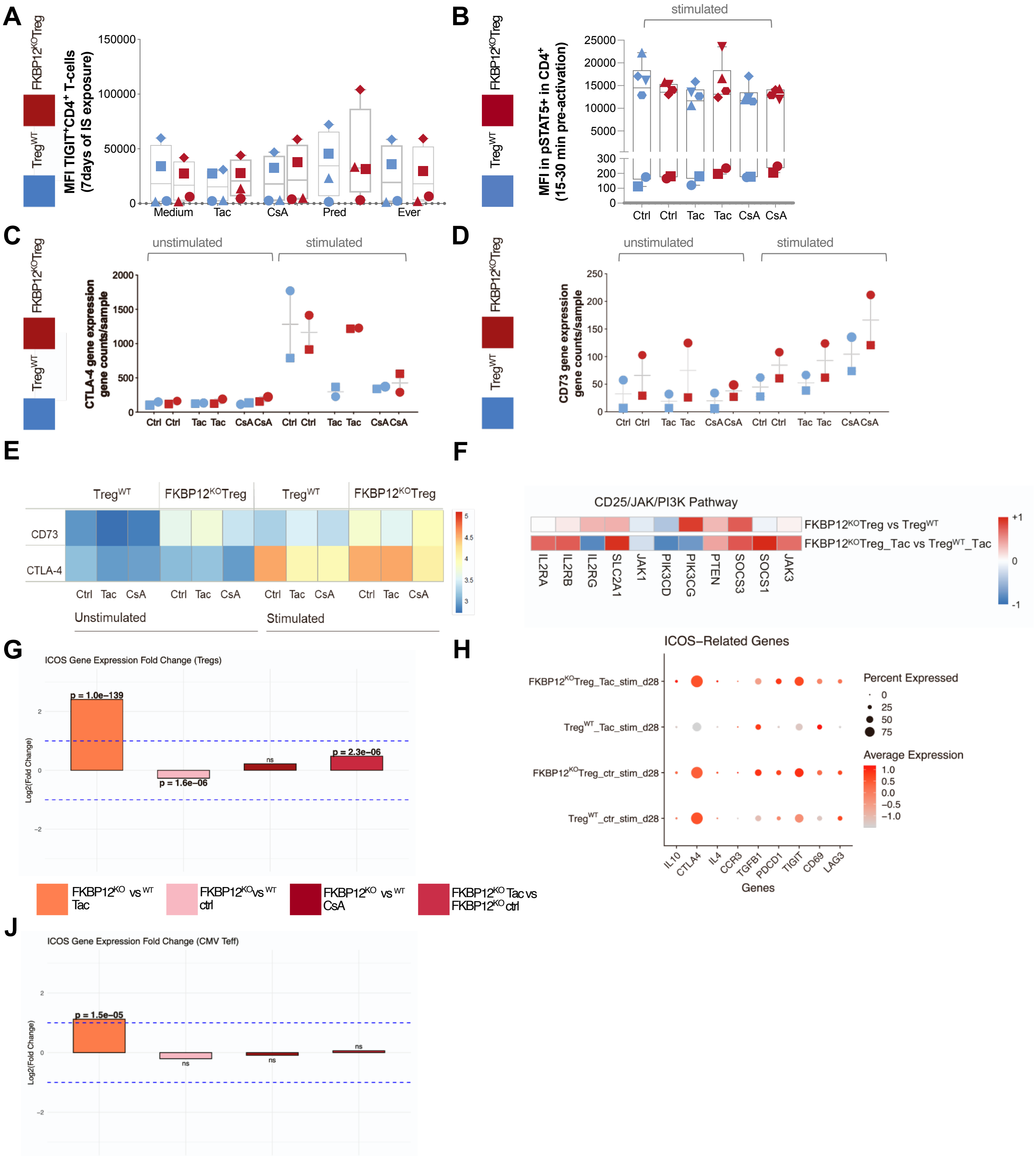

Figure S4: Beneficial functionality of FKBP12^{KO}-Treg in comparison to Treg^{WT}

Each symbol represents data from an individual donor. Tcon (grey), Treg^{WT} (blue), FKBP12^{KO}-Treg (red) n=2-6. For all figures conditions tacrolimus (Tac), cyclosporin A (CsA) and medium control (Ctrl) are shown. (A) Tigit expression in living CD4^{+} T-cells on day 21 in Treg^{WT} and FKBP12^{KO}-Treg under immunosuppressants including prednisolone (Pred) and everolimus (ever). (B) pSTAT5 MFI after 15-30 minutes stimulation by IL2 on day 21 in Treg^{WT} and FKBP12^{KO}-Treg substracted by unstimulated control. (C-D) CTLA-4 and CD73 gene expression levels are shown for Treg^{WT} and FKBP12^{KO}-Treg in unstimulated and stimulated states (6h PMA/Ionomycin activation) on day 28. Mean values of 2 technical replicates are displayed from 2 donors. (E) Heatmap of surface CD73 and CTLA-4 (CITE-seq antibody-derived tags) is shown for Treg^{WT} vs. FKBP12^{KO}-Treg in unstimulated and stimulated states (6h PMA/Ionomycin activation). (F) Heatmap visualizing differential expression for genes of JAK/STAT/PI3K pathways in FKBP12^{KO}-Tregs versus Treg^{WT} under tac and medium. Colour gradient = Log2 Fold Change (G) Bar graph shows Log2 Fold Change of ICOS expression in FKBP12^{KO} Tregs compared to Treg^{WT} under Tac, CsA and medium and a KO-only under Tac and medium. Statistics using non-parametric Wilcoxon rank-sum test. (H) Dot plot profiling an ICOS-related gene panel across FKBP12^{KO} and Treg^{WT} on day 28 for stimulated and unstimulated conditions. Size of dot = percentage of cells expressing target, colour intensity (light grey to red) = scaled average expression level. (I) Corresponding Log2 Fold Change of ICOS expression in FKBP12^{KO} CMV Teffs vs. WT across matching conditions. Counts aggregated into pseudo-bulk profiles and modelled using the edgeR quasi-likelihood (QL) framework with robust dispersion estimation. Horizontal lines indicate a 2-fold change (Log2FC = ±1). Exact adjusted p-values (ns = not significant; A = Bonferroni-adjusted; B = False Discovery Rate [FDR]).

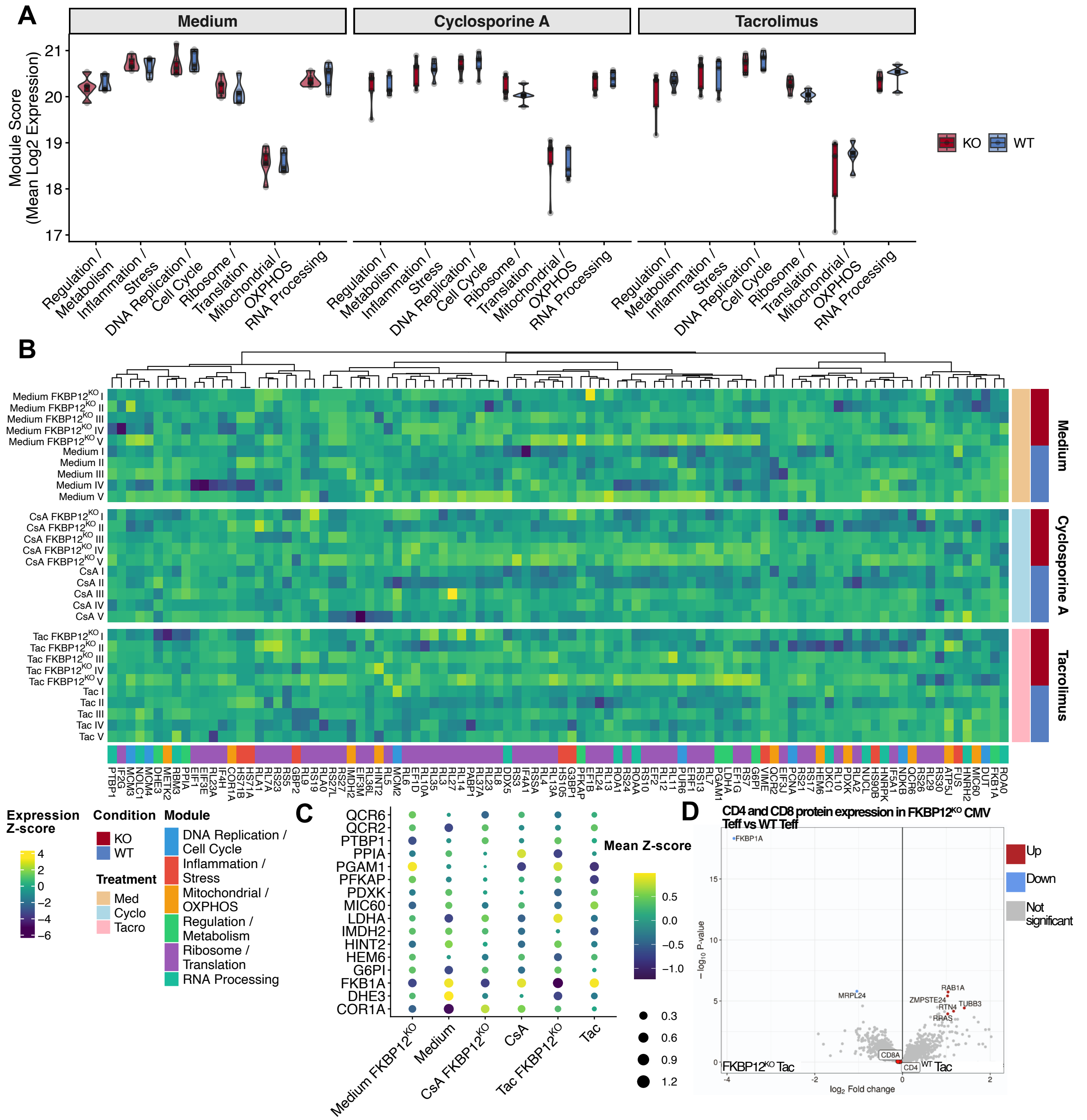

Figure S5: Comprehensive proteomic analysis reveals functional module differences between FKBP12^{KO}-Treg and Treg^{WT} under immunosuppressive treatment

(A) Violin plot showing comprehensive module analysis comparing FKBP12^{KO} (red) versus wild-type (blue) Tregs across three treatment conditions (Medium, cyclosporine A (CsA), and tacrolimus (Tac)). Six functional modules were analyzed: Regulation/Metabolism, Inflammation/Stress, DNA Replication/Cell Cycle, Ribosome/Translation, Mitochondrial/OXPHOS, and RNA Processing. Module scores represent the mean log2 expression values of constituent proteins. Each point = individual biological replicate (n=5 per group). Violin plots show the full distribution of data with overlaid boxplots and individual data points. (B) Heatmap displaying protein-level expression patterns across all experimental conditions. Rows represent individual proteins (n=98 unique proteins across all), coloured by functional module assignment (right annotation bar). Columns are organized by treatment condition Medium, CsA, Tac with wild-type (blue) and FKBP12^{KO} (red) samples grouped within each treatment (top annotation bars). Expression values are Z-score normalized across samples to highlight relative expression patterns. Proteins are hierarchically clustered using Euclidean distance and Ward's linkage method to group co-regulated proteins. The viridis color scale represents expression Z-scores from low (purple) to high (yellow). (C) Dotplot showing the mean expression Z-score of individual proteins within the Regulation/Metabolism module across all six experimental conditions. Each dot represents one protein averaged across n=5 biological replicates. Dot size encodes the absolute mean Z-score (|mean Z|), reflecting the magnitude of deviation from the protein's overall mean expression regardless of direction. Dot colour encodes the mean Z-score value, indicating both the direction and magnitude of expression change (viridis scale: purple = below average, yellow = above average). (D) volcano plot showing protein analysis of CD4 and CD8 protein in CMV Teffs is shown for FKBP12^{KO}-Teff (left) vs Teff^{WT} (right) with target genes labelled in light red, depicting Log2 Fold Change (FC) under tacrolimus treatment.

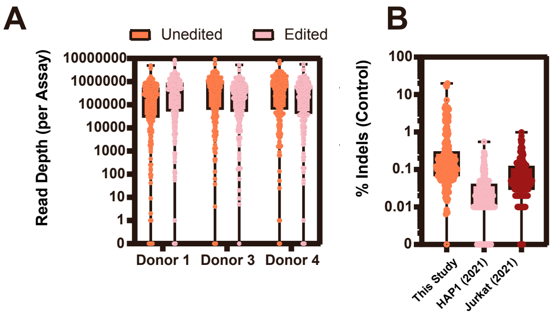

Figure S6: Off-target analysis of FKBP12^{KO}-Treg

Confirmatory sequencing information. (A) Following sequencing, the number of reads per off-target assay is shown for paired treatment and controls for each donor. (B) Indel frequencies of the unedited controls from all donors were quantified and compared to previously published frequencies in Kurgan et al. 2021 using the same bioinformatic analysis workflow.

Table S1: List of general antibodies
Summary of general Treg specific antibodies giving information on antibody, fluorescence label clone and concentration.

**Isolation and culture of Treg**	**Antibody**	**Fluorescence label**	**clone**	**concentration**
	anti-CD4	Vioblue	MT466	1 µg/mL
	anti-CD25	PE	3G10	1 µg/mL
	anti-CD127	APC	MB1518C9	1 µg/mL
**Phenotypic and functional assessment**	**Antibody**	**Fluorescence label**	**clone**	**concentration**
	anti-CD25	PE	MA251	1 µg/mL
	anti-CD127	APC	A019D5	2 µg/mL
	LIVE/DEAD™	UV	-	1:100 (manufacturer recommended)
	anti-CD3	BV650	OKT3	2 µg/mL
	anti-CD8	BV510	RPAT8	1 µg/mL
	anti-CD4	PerCP Cy5.5	SK3	1 µg/mL
	anti-FOXP3	AF488	259D	4 µg/mL
	anti-IL2	PE-Cy7	MQ117H12	4 µg/mL
	anti-TNFa	Alexa 700	MAb11	2 µg/mL
	anti-CD137	PE-Cy5	4B41	2 µg/mL
	anti-CD154	BV711	2431	2 µg/mL
	anti-IFNg	eFluor 450	4S.B3	2.5 µg/mL
	anti-CTLA4	PE-Cy5	BNI3	2 µg/mL
	anti-PD1	PE-Cy7	EH12.2H7	2 µg/mL

Table S2: List of materials and instruments
Summary of materials, supplements and instruments used giving information on material, concentration and company.

**Isolation and culture of Treg**	**Material/ Instrument**	**Company**	**Concentration**	**Additional information**
	EasySep™ Human CD4+ T-cell Enrichment Kit	Stemcell Technologies	-	
	CliniMACS Plus device	Miltenyi Biotec	-	
	anti-CD3/CD28-MACSiBeads	Miltenyi Biotec	-	
	X-Vivo-15	Lonza	-	
	fetal calf serum (FCS)	Biochrom	0,1ml/ml	
	penicillin/streptomycin	Biochrom	0,01ml/ml	
	rapamycin	Sigma Aldrich	1ul/ml	
	recombinant human IL-2	Miltenyi Biotec	1,250 IU/ml	
**FKBP12 knock-out in Treg**	**Material/ Instrument**	**Company**	**Concentration**	
	HiFi Cas9 Nuclease V3	IDT	10ug	
	2O'-methyl-3'phosphothioate-modified sgRNA	Synthego Corporation	15ug	Sequence: 5'-G*G*G*CGCACCUUCCCCAAGCG + mod-scaffold
	Amaxa-P3-primary-cell-4D-Nucleofector-X-Kit-L	ThermoFisher	-	
	Amaxa-Nucleofector-4D	Lonza	-	program CO-115
**Phenotypic and functional assessment**	**Material/ Instrument**	**Company**	**Concentration**	**Additional information**
	PMA		-	
	Ionomycin		-	
	FoxP3/Transcription Factor Staining Buffer kit	eBioscience	-	
	Fortessa	BD	-	
	Cytoflex LC	Beckman Coulter	-	
**Methylation profiling**	**Material/ Instrument**	**Company**	**Concentration**	

	Infinium-Methylation-EPIC-Kit	Illumina-EPIC-8-BeadChip	-	
	EZ-DNA-Methylation-Gold-Kit	Zymo Research	-	
	QIAquick-PCR-Purification-Kit	Qiagen	-	
**Cellular indexing of transcriptomes and epitopes sequencing (CITE-seq)**	**Material/ Instrument**	**Company**	**Concentration**	
	Single-Index-Kit-T-Set-A/Single-Index-Kit-N-Set A	10X Genomics	-	
	High-Sensitivity-DNA-Kit	Agilent	-	
	NextSeq500 device	Illumina	-	

Table S3: List of CITEseq antibodies
Summary of CITEseq antibodies giving information on reagent, clone, source and identifier.

REAGENT or RESOURCE	SOURCE	IDENTIFIER
Antibodies		
TotalSeq™-C0034 anti-human CD3 Antibody (UCHT1)	BioLegend	Cat# 300479
TotalSeq™-C0045 anti-human CD4 Antibody (SK3)	BioLegend	Cat# 344651
TotalSeq™-C0080 anti-human CD8a Antibody (RPA-T8)	BioLegend	Cat# 301071
TotalSeq™-C0185 anti-human CD11a (TS2/4)	BioLegend	Cat# 350617
TotalSeq™-C0085 anti-human CD25 (BC96)	BioLegend	Cat# 302649
TotalSeq™-C0386 anti-human CD28 (CD28.2)	BioLegend	Cat# 302963
TotalSeq™-C0176 anti-human CD39 (A1)	BioLegend	Cat# 328237
TotalSeq™-C0576 anti-human CD49d (9F10)	BioLegend	Cat# 304345
TotalSeq™-C0174 anti-human CD58 (TS2/9)	BioLegend	Cat# 330921
TotalSeq™-C0146 anti-human CD69 (FN50)	BioLegend	Cat# 310951
TotalSeq™-C0577 anti-human CD73 (AD2)	BioLegend	Cat# 344031
TotalSeq™-C0156 anti-human CD95 (DX2)	BioLegend	Cat# 305651
TotalSeq™-C0155 anti-human CD107a (H4A3)	BioLegend	Cat# 328649
TotalSeq™-C0246 anti-human CD122 (TU27)	BioLegend	Cat# 339021
TotalSeq™-C0390 anti-human CD127 (A019D5)	BioLegend	Cat# 351352
TotalSeq™-C0355 anti-human CD137 (4B4-1)	BioLegend	Cat# 309839
TotalSeq™-C0151 anti-human CD152 (BNI3)	BioLegend	Cat# 369621
TotalSeq™-C0032 anti-human CD154 (24-31)	BioLegend	Cat# 310849

TotalSeq™-C0140 anti-human CD183 (G025H7)	BioLegend	Cat# 353747
TotalSeq™-C0144 anti-human CD185 (J252D4)	BioLegend	Cat# 356939
TotalSeq™-C0071 anti-human CD194 (L291H4)	BioLegend	Cat# 359425
TotalSeq™-C0141 anti-human CD195 (J418F1)	BioLegend	Cat# 359137
TotalSeq™-C0143 anti-human CD196 (G034E3)	BioLegend	Cat# 353440
TotalSeq™-C0148 anti-human CD197 (G043H7)	BioLegend	Cat# 353251
TotalSeq™-C0152 anti-human CD223 (11C3C65)	BioLegend	Cat# 369335
TotalSeq™-C0171 anti-human CD278 (C398.4A)	BioLegend	Cat# 313553
TotalSeq™-C0088 anti-human CD279 (EH12.2H7)	BioLegend	Cat# 329963
TotalSeq™-C0169 anti-human CD366 (F38-2E2)	BioLegend	Cat# 345049
TotalSeq™-C0063 anti-human CD45RA (HI100)	BioLegend	Cat# 304163
TotalSeq™-C0087 anti-human CD45RO (UCHL1)	BioLegend	Cat# 304259
TotalSeq™-C0159 anti-human HLA-DR (L243)	BioLegend	Cat# 307663
TotalSeq™-C0089 anti-human TIGIT (A15153G)	BioLegend	Cat# 372729

Table S4: Database comparison of differentially expressed CITEseq genes in FKBP12^{KO}-Treg and Treg^{WT}

Summary of significantly regulated genes pre-selected from volcano plots comparing expression levels in FKBP12^{KO}-Treg and Treg^{WT}. Those genes further underwent DAVID database analysis and resulting gene dependent pathways are displayed.

Table S5: Off-target nomination sites

Summary of off-target nomination sites selected with the following criteria: 1) All sites that had overlap between one or more empirical method (abnoba-seq/guide-seq/UNCOVERseq); 2) All UNCOVERseq sites with Tiers 1 to 3 (our highest priorities); 3) All GUIDE-seq sites; 4) All Abnoba-seq sites; 5) All in silico sites with lev dist < 3 (reference or population >1%); 6) All in silico sites with lev dist <4 + an exonic region annotated (reference or population > 1%).

Table S6: Methods qualification of digital droplet PCR of FKBP12^{KO}-Treg

Qualification results for KO-efficacy read-outs for in total 4 assays indicating operator, date, assay, mean and standard deviation and reference Sanger-sequencing. Results qualification for KO-efficacy read-outs. Assays 1-4.

06.02. - 07.02.2025

Donor (D)																				
Operators	D1	D1	D2	D1																
Date	07.02.25	07.02.25	06.02.25	06.02.25	A1	A1	A1	A2	A2	A2	A3	A3	A3	A4	A4	A4				
Assay	A1	A2	A3	A4	50	75	100	50	75	100	50	75	100	50	75	100				
1	90,45	90,34	91,14	90,55	47,66	73,99	99,03	49,58	72,43	97,67	50,99	75,37	100,00	49,05	74,09	99,88				
2	90,37	90,32	91,54	90,10	47,93	73,09	98,55	47,60	72,34	97,25	50,04	75,16	100,00	48,90	74,22	99,80				
3	90,27	89,64	91,61	90,03																
4	90,02	89,80	90,99	89,77																
5	90,27	90,42	91,53	89,79																
6	90,57	90,05	90,89	90,28																
7	90,41	89,78	91,05	90,14																
8	89,60	90,08	91,34	89,06																
Mean	90,24	90,05	91,26	89,96		73,54			72,39			75,27			74,15					

standard deviation	0,31	0,29	0,28	0,44																
coefficient of variation	0,34	0,33	0,30	0,49																
Reference Sanger-seq.	93,00	91,00	99,00	92,00																
	Intra-A	Inter-A I	Inter-O I	Ges.	Inter-O II	Inter-A II	A1	A1	A1	A2	A2	A2	A3	A3	A3	A4	A4	A4	A1-A4	Sanger Seq.
	A1, A2	A1, A4	A3, A4	A1, A2, A3, A4	A1, A3	A2, A3	50	75	100	50	75	100	50	75	100	50	75	100	75	
Mean	90,15	90,10	90,61	90,38	90,75	90,66	47,80	73,54	98,79	48,59	72,39	97,46	50,52	75,27	100,00	48,98	74,15	99,84	73,84	93,75
standard deviation	0,31	0,40	0,76	0,62	0,60	0,68														
coefficient of variation	0,34	0,44	0,84	0,68	0,66	0,75														
% of deviation Assay vs. Reference																				-3,59

Supplemental methods

Computational Analysis – CITE seq

Differential Expression and Variance Modeling

To ensure maximum statistical rigor, the differential expression models in this study were dynamically adapted to the biological structure of the data and the sample sizes of the specific comparisons being queried. All frameworks strictly corrected for the multiple testing problem, completely avoiding the use of raw, unadjusted p-values.

1. Global Population Dynamics (edgeR & FDR)

For analyses evaluating broad transcriptional shifts across entire experimental conditions—including the Cytomegalovirus-specific effector T cell (CMV Teff) populations ($N=4$ donors), JAK/STAT pathway mapping, global cytokine downregulation, and overall Treg phenotypic profiling—we utilized a pseudo-bulk edgeR framework. By aggregating counts per biological replicate, this gold-standard approach accurately models true donor-to-donor variance and eliminates single-cell pseudoreplication. For CMV Teffs, counts were aggregated into pseudo-bulk profiles and differential expression was modeled using the edgeR quasi-likelihood (QL) framework with robust dispersion estimation. Significance for these comparisons was defined using the False Discovery Rate (FDR, Benjamini-Hochberg procedure) to robustly identify true biological shifts across the population.

2. Intra-Population Slicing & Subsetting (Wilcoxon & Bonferroni)

For highly granular queries requiring threshold-gated subsetting within a condition (e.g., isolating ‘ICOS-High’ versus ‘ICOS-Negative’ cells), pseudo-bulk aggregation carries a

severe risk of mathematical instability due to ‘cell starvation’ (i.e., generating pseudo-bulk profiles from statistically insufficient cell numbers per donor). Similarly, for the Treg compartment, the limited availability of biological replicates ($N=2$ donors) precluded reliable pseudo-bulk dispersion estimation. To bypass these sparsity limitations and maintain statistical power, we leveraged single-cell resolution using the non-parametric Wilcoxon rank-sum test. To aggressively guard against the inherent pseudoreplication of single-cell tests, we applied the highly conservative Bonferroni correction.

Transcriptional Profiling and Visualization

- ICOS Modulation Analysis: ICOS expression was evaluated as a Log2 Fold Change in FKBP12-KO cells compared to Wild-Type (WT) across distinct culture contexts (Tacrolimus, Cyclosporine A [CsA], and medium).
- Dot Plots: The size of each dot represents the percentage of cells within the condition expressing the target gene, while the color intensity (ranging from light grey to red or deep blue) indicates the scaled average expression level.
- Volcano Plots: Differential expression was depicted with target genes highlighted against a background of all detected transcripts (grey). The y-axis represents the $-Log_{10}$ adjusted p-value. Vertical dashed blue lines denote a 2-fold expression shift ($Log_{2}FC = \pm1$), and the horizontal dashed blue line signifies statistical significance (Adj. $p < 0.05$). Label overlap was algorithmically disabled to ensure comprehensive identification of all target markers.
- Pathway Heatmaps: Raw single-cell RNA counts were aggregated and averaged per condition. To highlight relative shifts in pathway activation (IFN-γ, IL-2, and TNF-α) without signal washout between the distinct cell lineages, expression

values were independently z-scored (scaled) across rows within each cell population prior to merging. Columns were explicitly ordered to visualize the biological progression from untouched controls to KO/Tacrolimus contexts, and genes exhibiting zero variance were algorithmically excluded to ensure matrix stability.

- Bubble Plots: Visualized differential expression for key members of the JAK/STAT and PI3K pathways. The color gradient indicates Log_{2} Fold Change, while bubble size is proportional to statistical certainty ($-Log_{10}$ FDR or Bonferroni, depending on the model).

Defined Gene Panels and Functional Subsetting

ICOS-High Subset: ICOS-positivity was defined mathematically by calculating the median expression threshold of all baseline non-zero expressing cells. This pre-defined ICOS-related gene panel was then profiled across conditions.

Phenotypic Markers: Tregs were profiled using established "Good" markers (e.g., FOXP3, MKI67, CTLA4) and "Bad" markers (e.g., IL7R/CD127, KLRG1) to assess stability. Additionally, cell-cycle and proliferation genes (CCND1, PCNA, MKI67) and terminal exhaustion receptors (TOX, LAG3, PDCD1) were tracked to confirm robust proliferation without inducing terminal exhaustion.

Cytokine Filtering: Cytokine and chemokine transcripts (IL, IFN, TNF, CXCL, CCL families) were strictly pre-filtered for significance and negative fold change ($LogFC < 0$).

Computational Analysis – Confirmation

Analysis of the sequencing data to identify confirmed off-target editing at the nominated sites was performed using CRISPAltRations v1.2.1. For identifying indels, the window for

event quantification was centered on the canonical cut site and events quantified utilizing the default window size for Cas9 (8 bp). To determine whether indels found in the sequencing data could result from bona fide off-target cleavage, indels were grouped by location relative to the cut site (prioritizing minimum distance to cut site) followed by fitting counts of events to a negative binominal model with a Wald test for significance in each location bin per off-target using the DESeq2 package within IDT's OTEasy tool (Schmaljohn et al., Manuscript in Preparation). For classification of indel off-target editing, the tool requires: 1) sufficient read coverage for the site (>1,000x) in all replicates, 2) significant edits to occur at or adjacent to the cut site after optimal alignment, 3) the classified cumulative significant edits to exceed 0.01%, 4) the comparison of treatment/control samples at the site to have a significant adjusted p-value ($p < 0.05$), and 5) an average coverage frequency of at least 5x the ascribed cumulative frequency observed (e.g., for 0.1% editing, at least 5,000x coverage).

Computational Analysis – Translocations

To quantify translocations from editing, Primer Anchored Statistical Translocation Analysis (PASTA) was used (Kurgan et al., Manuscript in Preparation). This analysis is only performed on the amplicon sequencing pools containing the on-target edit (because multiplexed amplification is a requirement for event detection using the method), and reactions not containing the on-target edit are unlikely to have any significant translocation events. To quantify translocations, expected primers were identified in reads using fg-idprimer (https://github.com/fulcrumgenomics/fg-idprimer; -k=6, -K=8, -S=5, --max-mismatch-rate=0.07). Following this, treatment/control pairs had their counts paired and primer count frequencies subjected to a one-tailed hypergeometric test with Benjamini-Hochberg correction (statsmodel v0.15.0; default settings) to calculate an adjusted p-

value (p-adj). Unexpected primer pairs with padj < 0.01 with no flags were classified as a translocation and had the translocation frequency (P) calculated using the following equation S1:

$$P_t = \frac{n_t}{f_{total}} + \frac{n_t}{r_{total}}$$

Where ‘n’ is equal to the count of the unexpected primer pair of interest, ‘t’ is the significant translocation being interpreted, ‘f’ is the total count of the shared forward primer events excluding the count participating in the ‘n’ translocation event, and ‘r’ is the total count of shared reverse primer events excluding the count participating in the ‘n’ translocation event. The translocation frequency is then adjusted by the background level frequency in the control by subtracting any translocation frequency observed in the control sample from the treatment frequency. Total translocation burden (B) was calculated using the following equation S2:

$$B = 1 - \prod_{t}^{t_n}(1 - P_t)$$

Where ‘t’ is equal to a significant translocation, and ‘tn’ is equal to the last significant translocation of all translocations. All translocations for the purposes of this equation are assumed to be occurring independently. Using the method, translocations are quantified if: 1) the estimated frequency exceeds 0.1% of editing, 2) the translocation has a significant p-value ($p < 0.01$), and 3) the translocation is found to meet these criteria in all replicates.
